# Targeted exome sequencing of Korean triple-negative breast cancer reveals homozygous deletions associated with poor prognosis of adjuvant chemotherapy-treated patients

**DOI:** 10.18632/oncotarget.18618

**Published:** 2017-06-27

**Authors:** Hae Min Jeong, Ryong Nam Kim, Mi Jeong Kwon, Ensel Oh, Jinil Han, Se Kyung Lee, Jong-Sun Choi, Sara Park, Seok Jin Nam, Gyung Yup Gong, Jin Wu Nam, Doo Ho Choi, Hannah Lee, Byung-Ho Nam, Yoon-La Choi, Young Kee Shin

**Affiliations:** ^1^ Department of Pharmacy, College of Pharmacy, Seoul National University, Seoul, South Korea; ^2^ Tumor Microenvironment Global Core Research Center, Seoul National University, Seoul, South Korea; ^3^ College of Pharmacy, Kyungpook National University, Daegu, South Korea; ^4^ Research Institute of Pharmaceutical Sciences, College of Pharmacy, Kyungpook National University, Daegu, South Korea; ^5^ Laboratory of Cancer Genomics and Molecular Pathology, Samsung Biomedical Research Institute, Samsung Medical Center, Seoul, South Korea; ^6^ Department of Pathology and Translational Genomics, Samsung Medical Center, Sungkyunkwan University School of Medicine, Seoul, South Korea; ^7^ Gencurix Inc., Seoul, South Korea; ^8^ Department of Surgery, Samsung Medical Center, Sungkyunkwan University School of Medicine, Seoul, South Korea; ^9^ The Center for Anti-Cancer Companion Diagnostics, Bio-MAX/N-Bio, Seoul National University, Seoul, South Korea; ^10^ Department of Pathology, Asan Medical Center, University of Ulsan College of Medicine, Seoul, South Korea; ^11^ Department of Life Science, College of Natural Sciences, Hanyang University, Seoul, South Korea; ^12^ Department of Radiation Oncology, Samsung Medical Center, Sungkyunkwan University School of Medicine, Seoul, South Korea; ^13^ Interdisciplinary Program in Bioinformatics, College of Natural Science, Seoul National University, Seoul, South Korea; ^14^ HERINGS, The Institute of Advanced Clinical & Biomedical Research, Seoul, South Korea; ^15^ Department of Health Sciences and Technology, Samsung Advanced Institute for Health Sciences and Technology, Sungkyunkwan University, Seoul, South Korea; ^16^ The Center for Anti-Cancer Companion Diagnostics, School of Biological Science, Institutes of Entrepreneurial BioConvergence, Seoul National University, Seoul, South Korea; ^17^ Research Institute of Pharmaceutical Sciences, College of Pharmacy, Seoul National University, Seoul, South Korea

**Keywords:** triple-negative breast cancer, targeted exome sequencing, single nucleotide variant, copy number variation, DNA repair pathway

## Abstract

Triple-negative breast cancer is characterized by the absence of estrogen and progesterone receptors and human epidermal growth factor receptor 2, and is associated with a poorer outcome than other subtypes of breast cancer. Moreover, there are no accurate prognostic genes or effective therapeutic targets, thereby necessitating continued intensive investigation. This study analyzed the genetic mutation landscape in 70 patients with triple-negative breast cancer by targeted exome sequencing of tumor and matched normal samples. Sequencing showed that more than 50% of these patients had deleterious mutations and homozygous deletions of DNA repair genes, such as *ATM*, *BRCA1*, *BRCA2*, *WRN*, and *CHEK2*. These findings suggested that a large number of patients with triple-negative breast cancer have impaired DNA repair function and that therefore a poly ADP-ribose polymerase inhibitor may be an effective drug in the treatment of this disease. Notably, homozygous deletion of three genes, *EPHA5*, *MITF*, and *ACSL*3, was significantly associated with an increased risk of recurrence or distant metastasis in adjuvant chemotherapy-treated patients.

## INTRODUCTION

Breast cancer is one of the most prevalent cancers worldwide, with over 1,300,000 newly diagnosed patients and 450,000 deaths each year [1]. Breast cancer is a highly heterogeneous disease with diverse pathophysiological and clinical features that can be caused by distinct genetic, epigenetic, and transcriptomic changes. Based on expression of estrogen receptor (ER), progesterone receptor (PR), and human epidermal growth factor receptor 2 (HER2), breast cancer can be categorized into three subtypes: hormone receptor-positive (ER+ or PR+), HER2-positive (ER-, PR-, and HER2+), and triple-negative breast cancer (TNBC) (ER-, PR-, and HER-) types [2, 3]. TNBC accounts for approximately 10–20% of invasive breast cancers, and the mortality rate of women with TNBC during the 5 years after diagnosis is high [4, 5]. Based on ethnicity, breast cancer incidence rates are higher in Caucasian than in African-American, Hispanic, and Asian women. However, aggressive and advanced-stage breast cancer diagnosed at an early age, in particular TNBC, is more frequent in African-American than in Caucasian women [6].

Although agents targeting hormone receptors and HER2 can be used to treat hormone receptor-positive and HER2-positive types of breast cancer, these agents are ineffective against TNBC because of the absence of the targeted receptors (ER, PR, and HER2) [7, 8]. Despite several pioneering genome-wide studies that aimed to identify diagnostic and therapeutic biomarkers in TNBC, there has been no comprehensive effort to date that has attempted to identify TNBC biomarkers in the Korean population [9–11]. Because there is no conventional therapy targeting TNBC, studies that intensively evaluate genomic alterations are essential to identify novel prognostic biomarkers and/or therapeutic targets for TNBC.

Owing to its greater cost-effectiveness than whole genome or whole exome sequencing, targeted exome sequencing has recently revolutionized human clinical cancer diagnosis, facilitated studies towards understanding cancer-causing mechanisms, and enabled the identification of therapeutic targets [12–15]. In particular, the HaloPlex target enrichment system for targeted exome sequencing has shown high efficiency in capturing targeted regions on the exome and high library complexity [16].

This study was designed to characterize the somatic mutation profiles of 368 cancer-associated genes in 70 Korean patients with TNBC and to identify novel somatic mutations and potential prognostic genes. We found that more than half of the patients in our cohort had deleterious mutations in several DNA repair-related genes, suggesting that poly ADP-ribose polymerase (PARP) inhibitors may be effective in treating patients with TNBC therapy. Moreover, we identified three candidate prognostic genes whose homozygous deletions were significantly associated with the prognosis of patients who had been treated with adjuvant chemotherapy.

## RESULTS

### Analysis of somatic single nucleotide variants and small insertions and deletions

Clinicopathological characteristics of the 70 patients with TNBC included in this study are described in Table 1. Of these patients, 15 (21%) experienced tumor recurrence, including eight with distant metastases. The mean follow-up period was 4.88 years. We determined whether clinicopathological factors, such as age, primary tumor stage (pT), and lymph node metastasis, were associated with patient outcomes, including disease-free survival (DFS) and distant metastasis-free survival (DMFS), finding no evidence of association between these factors and either DFS or DMFS (Supplementary Table 1).

**Table 1:** Clinicopathological features of 70 Korean patients with triple-negative breast cancer

	Parameter	n (%)
Age, yr		
	(mean ± S.D.)	48.0±10.4
	<50	39 (55.7)
	≥50	31 (44.3)
Postmenopause		
	No	41 (58.6)
	Yes	22 (31.4)
	NA	7 (10.0)
pT		
	1	29 (41.4)
	2	38 (54.3)
	3	3 (4.3)
Lymph node metastasis		
	No	32 (45.7)
	Yes	38 (54.3)
Pathologic stage		
	I	14 (20.0)
	II	44 (62.9)
	III	12 (17.1)
Lymphatic invasion		
	No	44 (62.9)
	Yes	26 (37.1)
Recurrence		
	No	55 (78.6)
	Yes	15 (21.4)
Type of surgery		
Conserving surgery		26 (37.1)
Partial mastectomy & sentinel node biopsy		31 (44.3)
Modified radical mastectomy		10 (14.3)
Total mastectomy		3 (4.3)
Adjuvant radiotherapy		
	No	13 (18.6)
	Yes	57 (81.4)
Adjuvant chemotherapy		
	No	3 (4.3)
	Yes	67 (95.7)
Total		
		70 (100.0)
Average F/U		
	(mean ± S.D.)	4.88±1.34

pT, primary tumor stage; F/U, follow-up.

**Figure 1:**
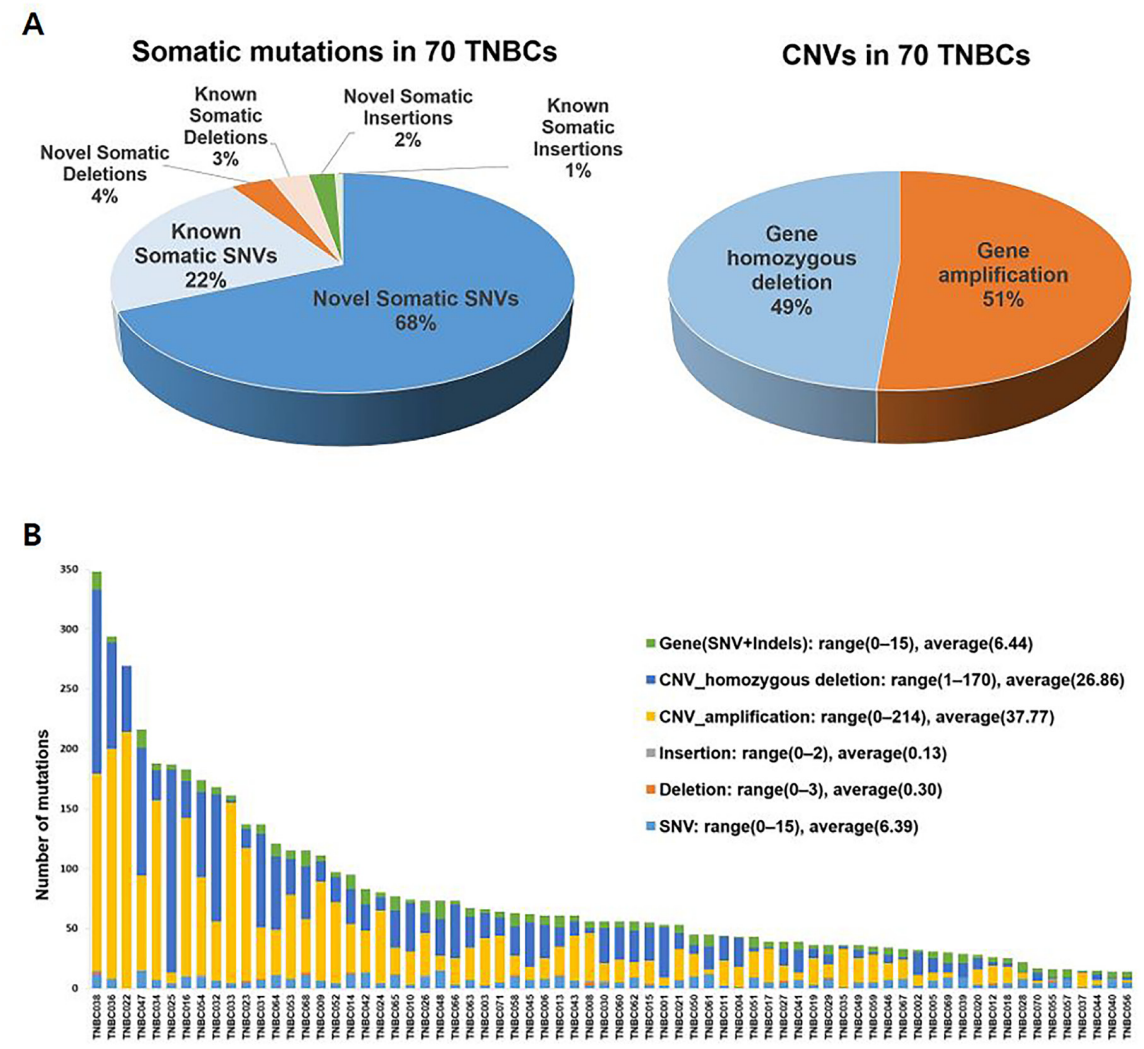
Somatic SNVs and CNVs in genomes of 70 Korean patients with TNBC **(A)** Percentages of types of somatic mutations, showing that a high percentage consisted of novel somatic SNVs. **(B)** Numbers of somatic SNVs and CNVs in individual patients. The numbers of genes with homozygous deletions per patient ranged from 1 to 170, whereas the numbers of amplified genes per patient ranged from 0 to 214. SNVs, single nucleotide variants; CNVs, copy number variations; TNBC, triple-negative breast cancer.

**Figure 2:**
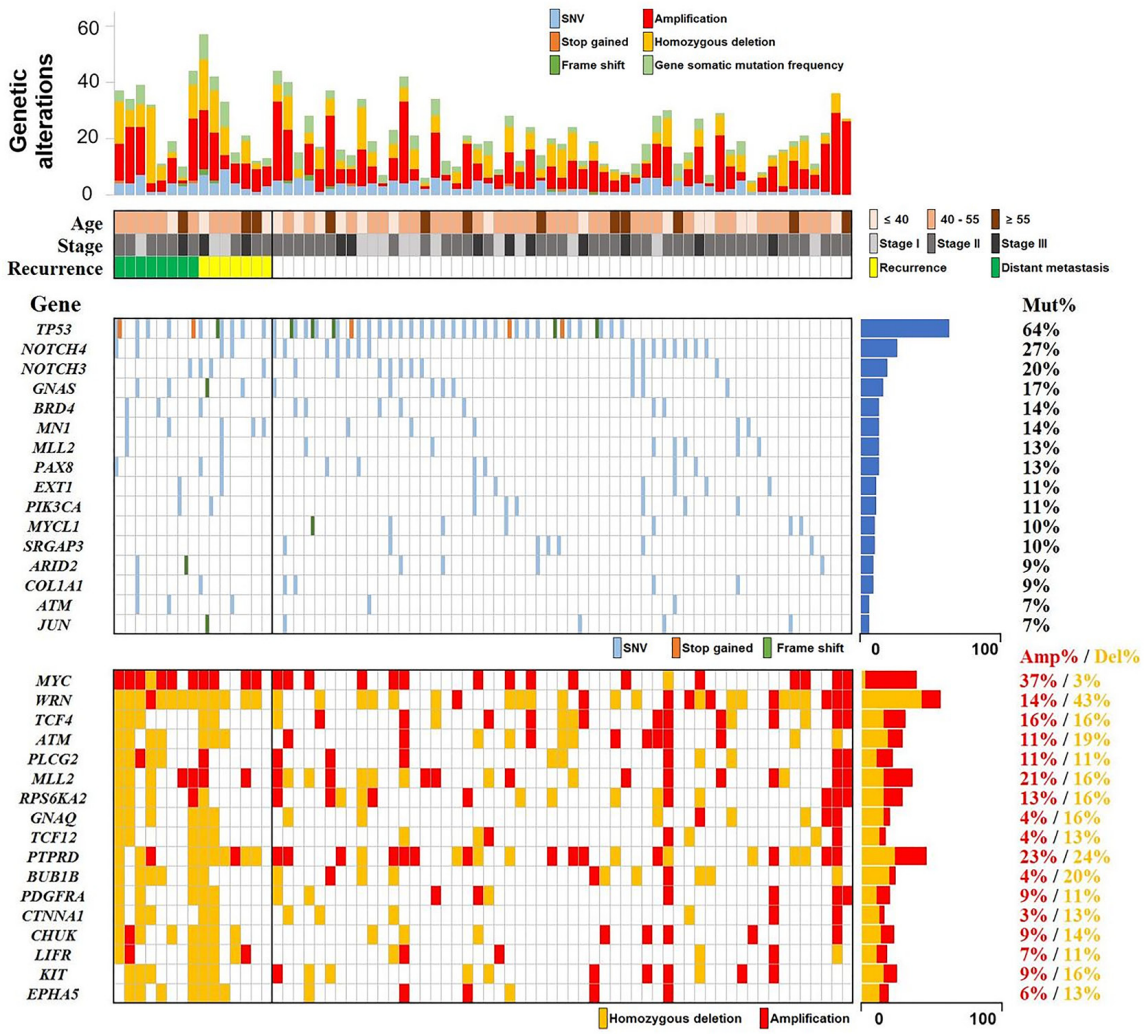
Landscape of the most frequent somatic SNVs and CNVs Summaries ofthe most frequently occurring somatic SNVs and CNVs in the study cohort. TP53 was the most frequently mutated gene with stop-gain and frameshift mutations. SNVs, single nucleotide variants; CNVs, copy number variations.

The average target coverage depths were 130.36× for tumor samples and 139.71× for normal samples, and target regions with read coverage depths >2× and >100× accounted for over 93% and over 40%, respectively, of the entire target region (Supplementary Table 2). Analysis showed 292 somatic single nucleotide variants (SNVs) and 30 somatic small insertions and deletions (INDELs) in 157 genes. Of these variants, 238 mutations were novel SNVs or INDELs that had not been reported previously in either the COSMIC or dbSNP database (Figure 1A, Supplementary Table 3). Supplementary Table 4 lists all somatic SNVs and INDELs, whereas Tables 2 and 3 list frequently mutated genes and somatic SNVs and INDELs, respectively. Of the 70 patients, five (7%) had stop-gain mutations and six (9%) had frameshift mutations in *TP53*. Frameshift mutations were also detected in four other genes, *GNAS*, *ARID2*, *JUN*, and *MYCL1* (Figure 2). Sanger capillary sequencing validated two somatic mutations in *TP53* (c.637C>T and c.578A>G; Supplementary Figure 1).

**Table 2:** List of the most frequently mutated genes in the 70 patients with triple-negative breast cancer

Somatically Mutated Genes		Amplified Genes		Homozygously Deleted Genes	
Gene	Frequency (%)	Gene	Frequency (%)	Gene	Frequency (%)
*TP53*	45 (64)	*NDRG1*	36 (51)	*WRN*	30 (43)
*NOTCH4*	19 (27)	*UBR5*	32 (46)	*IL6ST*	22 (31)
*NOTCH3*	14 (20)	*PTK2*	32 (46)	*APC*	21 (30)
*GNAS*	12 (17)	*RECQL4*	26 (37)	*PTK2B*	20 (29)
*BRD4*	10 (14)	*MYC*	26 (37)	*NF1*	19 (27)
*MN1*	10 (14)	*IKBKE*	25 (36)	*SETD2*	18 (26)
*MLL2*	9 (13)	*EXT1*	25 (36)	*PTPRD*	17 (24)
*PAX8*	9 (13)	*CDK2*	24 (34)	*PBRM1*	17 (24)
*EXT1*	8 (11)	*NTRK1*	24 (34)	*MLL3*	16 (23)
*PIK3CA*	8 (11)	*DDR2*	22 (31)	*PCM1*	16 (23)
*ETV4*	7 (10)	*MCL1*	22 (31)	*PLD2*	15 (21)
*GLI3*	7 (10)	*TPR*	20 (29)	*PIK3R1*	15 (21)
*HOOK3*	7 (10)	*PARP1*	19 (27)	*CDK2*	14 (20)
*MYCL1*	7 (10)	*TPM3*	19 (27)	*CSF1R*	14 (20)
*SRGAP3*	7 (10)	*PRCC*	19 (27)	*BUB1B*	14 (20)
*ARID2*	6 (9)	*RNF213*	19 (27)	*CDK12*	14 (20)
*COL1A1*	6 (9)	*ERC1*	19 (27)	*MTOR*	13 (19)
*MTOR*	6 (9)	*FH*	18 (26)	*CHEK2*	13 (19)
*TRIM62*	6 (9)	*NBN*	18 (26)	*ATM*	13 (19)
*ATM*	5 (7)	*RGL1*	17 (24)	*RB1*	13 (19)
*BAP1*	5 (7)	*PTPRD*	16 (23)	*MAP3K1*	13 (19)
*JUN*	5 (7)	*TIAM1*	16 (23)	*TIAM1*	12 (17)
*KDM5C*	5 (7)	*NOTCH4*	16 (23)	*ERCC2*	12 (17)
*PPP2R1A*	5 (7)	*IGF1R*	16 (23)	*KTN1*	12 (17)
*BRCA2*	4 (6)	*IKBKB*	16 (23)	*BRCA1*	12 (17)
*CDKN2A*	4 (6)	*GATA3*	16 (23)	*TSHR*	12 (17)
*FGFR3*	4 (6)	*PBX1*	16 (23)	*MLL2*	11 (16)
*GRIN2D*	4 (6)	*MLL2*	15 (21)	*PRKDC*	11 (16)
*MAP3K1*	4 (6)	*FLT4*	15 (21)	*TCF4*	11 (16)
*MAPK8IP3*	4 (6)	*EGFR*	15 (21)	*USP6*	11 (16)
*PIK3R1*	4 (6)	*RPTOR*	15 (21)	*RPS6KA2*	11 (16)
*PTCH1*	4 (6)	*RUNX1T1*	15 (21)	*TAF1*	11 (16)
*RPTOR*	4 (6)	*COX6C*	15 (21)	*KIT*	11 (16)
*SFPQ*	4 (6)	*FLNA*	14 (20)	*MAP2K2*	11 (16)
*AKAP9*	3 (4)	*TSC2*	14 (20)	*EML4*	11 (16)
*ATRX*	3 (4)	*ATR*	14 (20)	*RPS6KA3*	11 (16)
*BAX*	3 (4)	*MAML2*	14 (20)	*GNAQ*	11 (16)
*BRD3*	3 (4)	*NTRK3*	14 (20)	*KIAA1549*	10 (14)
*CD74*	3 (4)	*CRTC3*	14 (20)	*PMS1*	10 (14)
*CDKN1A*	3 (4)	*TFEB*	14 (20)	*BRCA2*	10 (14)
*CIC*	3 (4)	*MLL3*	13 (19)	*CHUK*	10 (14)
*EGFR*	3 (4)	*ERCC2*	13 (19)	*ALDH2*	10 (14)
*EPHA5*	3 (4)	*SMARCA4*	13 (19)	*FGFR3*	10 (14)
*FLNA*	3 (4)	*EP300*	13 (19)	*TP53*	10 (14)

**Table 3:** List of the most frequently identified somatic mutations in 70 Korean patients with triple-negative breast cancer

Gene	Nucleotide Change	Amino Acid Change	Frequency (%)	Mutation Type	Reported	Mutation Assessment					
						SIFT score	PolyPhen2		LRT score	Mutation Taster score	Mutation Assessor score
							HDIV pred	HVAR pred			
*NOTCH4*	c.625T>G	p.T209P	9 (13)	Heterozygous	Novel	0.01	D	D	0.1942	0.7871	1.3850
*ETV4*	c.770T>G	p.V257G	7 (10)	Heterozygous	Novel	0.11	P	P	0.0134	0.8814	1.9950
*EXT1*	c.148T>G	p.S50R	7 (10)	Heterozygous	Novel	0.74	B	B	0.0025	0.3789	0.0000
*GNAS*	c.1264T>C	p.S422P	7 (10)	Heterozygous	Novel	0.18	B	B	0.0000	0.0000	1.5250
*NOTCH3*	c.6841C>G	p.A2281P	7 (10)	Heterozygous	Novel	0.86	P	B	NA	0.5542	0.0000
*COL1A1*	c.3746T>C	p.E1249G	6 (9)	Heterozygous	Novel	0.00	P	B	0.0000	0.7868	3.4800
*MLL2*	c.2482G>C	p.P828A	6 (9)	Heterozygous	Novel	0.00	B	B	NA	NA	0.5500
*TP53*	c.1103A>C	p.H368P	6 (9)	Heterozygous	Novel	0.21	B	B	0.4522	0.0857	0.3450
*ARID2*	c.3803A>C	p.N1268T	5 (7)	Heterozygous	Novel	0.00	B	B	0.0000	0.9744	0.9750
*NOTCH4*	c.118T>G	p.T40P	5 (7)	Heterozygous	Novel	0.03	B	B	0.1892	0.9635	2.5850
*BRD4*	c.2470T>G	p.T824P	4 (6)	Heterozygous	Novel	0.12	B	B	0.1482	0.0008	-0.6900
*GLI3*	c.2687T>G	p.D896A	4 (6)	Heterozygous	Novel	0.00	D	D	0.0000	1.0000	2.8350
*HOOK3*	c.62A>C	p.Q21P	4 (6)	Heterozygous	Novel	0.07	D	D	0.0000	0.8988	2.4150
*MN1*	c.2780G>A	p.T927R	4 (6)	Heterozygous	Novel	0.15	D	D	0.0000	0.9374	0.8050
*MTOR*	c.5480T>G	p.N1827T	4 (6)	Heterozygous	Novel	0.46	B	B	0.0234	0.0171	0.3450
*NOTCH4*	c.3064C>G	p.A1022P	4 (6)	Heterozygous	Novel	NA	D	P	0.0106	0.8376	0.5500
*PAX8*	c.695A>C	p.H232P	4 (6)	Heterozygous	Novel	0.02	B	B	0.2301	0.0635	0.2050
*PPP2R1A*	c.584T>G	p.V195G	4 (6)	Heterozygous	Novel	0.07	P	B	0.0000	1.0000	2.9600
*TRIM62*	c.1094T>G	p.I365S	4 (6)	Heterozygous	Novel	0.00	D	D	0.0000	0.9990	2.4750
*ATM*	c.6337A>C	p.T2113P	3 (4)	Heterozygous	Novel	0.28	B	B	0.6501	0.0022	0.0000
*BAP1*	c.626T>G	p.V209G	3 (4)	Heterozygous	Novel	0.00	D	D	0.0000	1.0000	3.5250
*CD74*	c.455T>G	p.L152R	3 (4)	Heterozygous	Novel	1.00	P	P	0.7923	0.0338	1.1000
*CDKN1A*	c.93C>A	p.S31R	3 (4)	Homozygous	dbSNP	0.99	B	B	0.9321	0.0024	-0.1300
*KDM5C*	c.2254A>C	p.T752P	3 (4)	Heterozygous	Novel	0.17	B	B	0.0000	0.7922	1.9150
*MAP3K14*	c.2024A>C	p.H675P	3 (4)	Heterozygous	Novel	0.00	D	B	0.0000	0.0000	0.0000
*MAPK8IP3*	c.763T>C	p.S255P	3 (4)	Heterozygous	Novel	0.01	B	B	0.0002	0.9997	1.8950
*MCL1*	c.116A>G	p.E39G	3 (4)	Heterozygous	Novel	0.54	B	B	0.0000	0.0005	-0.5500
*MN1*	c.2773G>A	p.E925K	3 (4)	Heterozygous	Novel	0.29	D	P	0.0000	0.4251	0.5500
*NOTCH3*	c.6865G>C	p.A2289P	3 (4)	Heterozygous	dbSNP	0.37	B	B	NA	0.5542	0.0000
*PAX8*	c.665A>C	p.H222P	3 (4)	Heterozygous	Novel	0.12	P	B	0.0014	0.6003	1.5450
*PAX8*	c.734T>G	p.Y245S	3 (4)	Heterozygous	Novel	0.03	P	B	0.0168	0.2135	1.8800
*PIK3CA*	c.3140A>G	p.H1047R	3 (4)	Heterozygous	dbSNP	0.16	P	B	0.0000	0.9999	0.0000
*PIK3CA*	c.821G>A	p.R274K	3 (4)	Heterozygous	dbSNP	0.03	D	P	0.0000	0.9997	2.1750
*PIK3R1*	c.367G>C	p.A123P	3 (4)	Heterozygous	Novel	0.21	B	B	0.0006	0.8999	1.3550
*RPTOR*	c.2557A>C	p.T853P	3 (4)	Heterozygous	Novel	0.29	B	B	0.0001	0.4881	1.5900
*SRGAP3*	c.3116T>C	p.F1039S	3 (4)	Heterozygous	Novel	0.26	B	B	0.0000	0.8194	1.7500
*TP53*	c.821G>T	p.R273L	3 (4)	Heterozygous	dbSNP	0.00	D	D	0.0000	1.0000	3.1450
*TP53*	c.746G>A	p.R248Q	3 (4)	Heterozygous	dbSNP	0.01	D	D	0.0000	1.0000	2.9700

B, benign; D, probably damaging; NA, not available; P, possibly damaging.

Because deleterious germline mutations in *BRCA1* and *BRCA2* have been significantly associated with breast cancer [17, 18], we assessed whether germline mutations in these two genes were present in our cohort. We found two deleterious germline mutations in *BRCA1* in three patients, and one stop-gain germline mutation in *BRCA2* in one patient (Supplementary Table 5). *BRCA1* c.922_924delAGCinsT (p.Ser308fs), found in two patients, and *BRCA2* c.8363G>A (p.W2788X), found in another patient, are mutations shown to have highly detrimental clinical impact [19–21], whereas *BRCA1* c.279delA (p.Phe93fs), found in a fourth patient, was identified as a novel germline frameshift mutation.

### Analysis of copy number variations

Copy number variation (CNV) analysis identified an average of 37.77 (range, 0–214) amplified genes and 26.86 (range, 1–170) homozygously deleted genes per patient (Figure 1B). Supplementary Table 6 lists all genes with CNV amplifications and homozygous deletions, whereas Table 2 lists the most frequently altered of these genes. Homozygous deletion of *TP53*, a tumor suppressor gene with the highest mutation frequency in this study, was observed in ten patients with TNBC, indicating that 55 (79%) of the 70 patients in our study cohort had either mutated or deleted *TP53*. Frequent amplification of *NDRG1* and deletion of *WRN* and *ATM* were validated by qPCR (Supplementary Figure 2).

In addition to the deleterious germline mutations described previously, somatic homozygous deletions of *BRCA1* and *BRCA2* were observed in the genomes of 12 and 10 patients, respectively (Table 2, Supplementary Table 5). Some of these homozygous deletions were limited to a single exon, whereas other encompassed several exons (Supplementary Figure 3).

### Association of homozygous deletions with clinical outcomes

**Figure 3:**
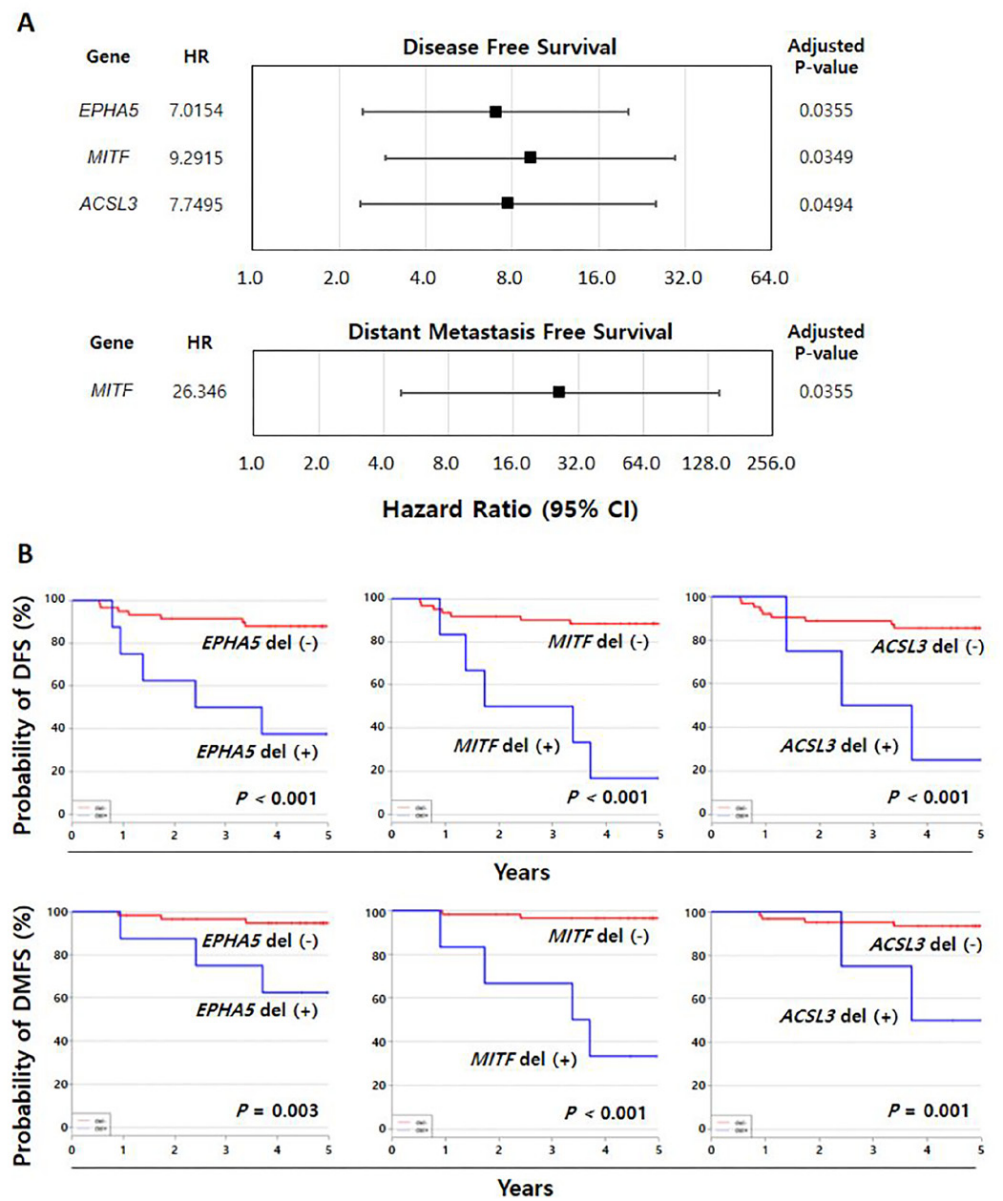
Proportional hazard ratio analysis of the association between prognosis and homozygous deletions **(A)** Homozygous deletions of nine genes were significantly associated with prognosis in the study cohort. **(B)** Kaplan–Meier analyses of DFS and DMFS, showing that homozygous deletions of *EPHA5*, *MITF*, and *ACSL3* were significantly associated with poor patient prognosis. DFS, disease-free survival; DMFS, distant metastasis-free survival; CI, confidence interval; HR, hazard ratio.

Using a Cox proportional-hazards regression model, we determined whether these somatic mutations were associated with the prognosis of the 67 patients who had been treated with adjuvant chemotherapy. We found that homozygous deletion of the three genes identified in our study was associated with an increased risk of recurrence or distant metastasis in patients with TNBC (Supplementary Table 7). Figure 3A shows the hazard ratios (HRs) and 95% confidence intervals (CIs) of each gene for DFS and DMFS. In addition, Kaplan–Meier analysis was performed to confirm the association between homozygous deletions of these three genes and poor prognosis. These analyses showed that homozygous deletions of *EPHA5* (*P* < 0.001 for DFS; *P* = 0.003 for DMFS), *MITF* (*P* < 0.001 for DFS; *P* < 0.001 for DMFS), and *ACSL3* (*P* < 0.001 for DFS; *P* = 0.001 for DMFS) were significantly associated with a negative prognosis in patients with TNBC (Figure 3B).

### The cancer genome atlas data analysis

**Figure 4:**
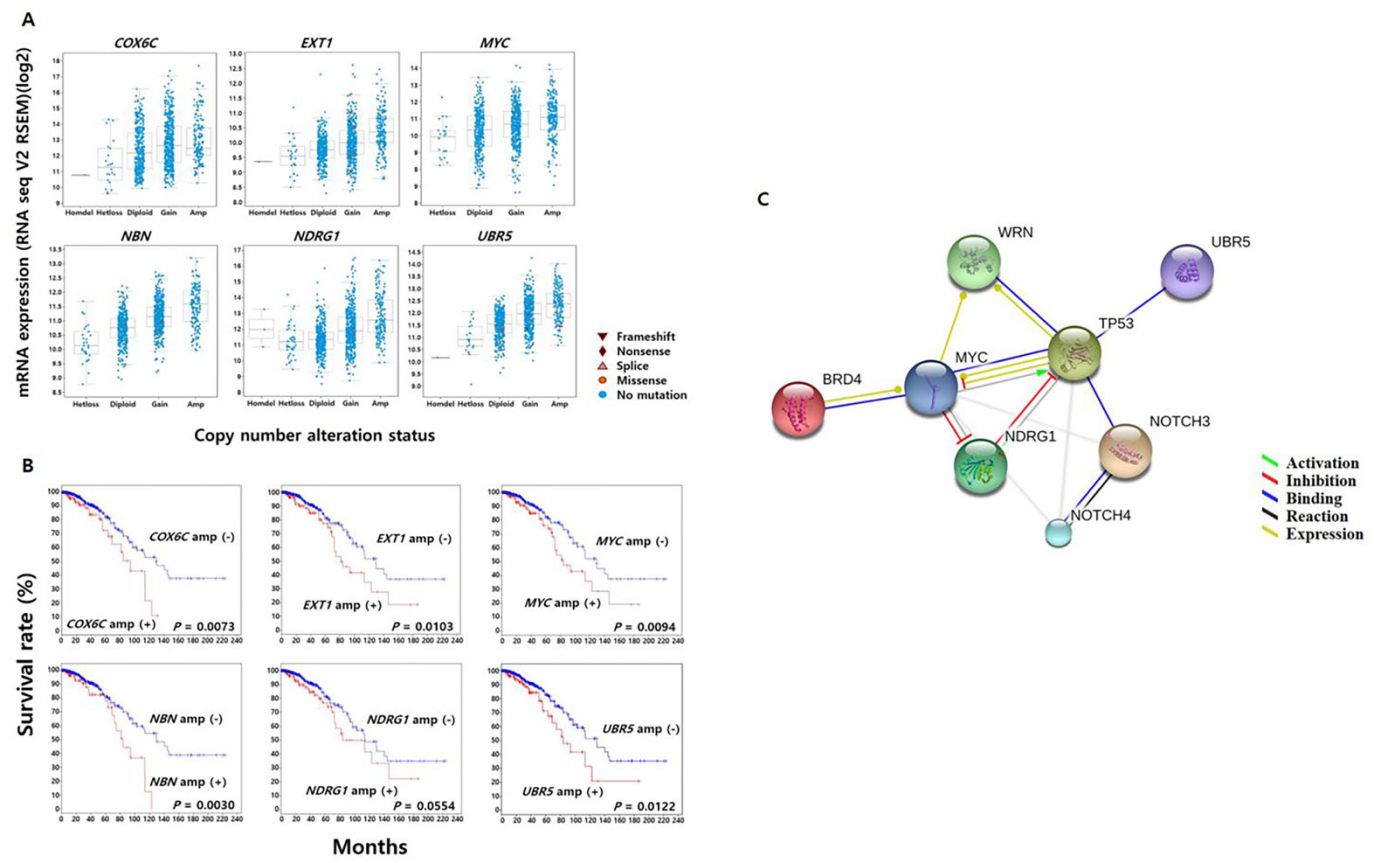
The Cancer Genome Atlas (TCGA) breast cancer data analysis **(A)** Relationships between genomic copy number gain and amplification status of *COX6C*, *EXT1*, *MYC*, *NBN*, *NDRG1*, and *UBR5* in clinical breast cancer samples and their respective levels of mRNA expression. **(B)** Survival analysis showing the decreased survival rate of breast cancer patients with gene amplifications. **(C)** Frequently mutated genes in the study cohort, including *TP53*, *WRN*, *MYC*, and *NDRG1*, involved in the DNA damage response pathway.

Associations between levels of mRNA expression and copy number alteration of genes identified as frequently amplified in our 70 Korean TNBC samples were analyzed using CNV and mRNA expression data from The Cancer Genome Atlas (TCGA) breast cancer database. We found that copy number gain or amplification of six genes (*NDRG1*, *UBR5*, *MYC*, *EXT1*, *NBN*, and *COX6C*) was positively correlated with high mRNA expression (Figure 4A). Kaplan–Meier analysis showed that the overall survival rates were significantly lower in breast cancer patients with than without amplification of one of these genes (log rank test; *NDRG1*, *P* = 0.0554; *UBR5*, *P* = 0.0122; *MYC*, *P* = 0.0094; *EXT1*, *P* = 0.0103; *NBN*, *P* = 0.0030; and *COX6C*, *P* = 0.0073) (Figure 4B).

Next, we used STRING (Search Tool for the Retrieval of Interacting Genes/Proteins) v.10 [22] to perform a network interaction analysis of proteins encoded by these genes with the most frequent genetic alterations (i.e., somatic non-synonymous mutations and CNVs) in our cohort of 70 Korean patients with TNBC. We found that DNA damage response genes, such as *TP53* and *WRN*, were frequently mutated in our TNBC cohort (Figure 4C). Notably, mutual exclusivity analysis using 500 clinical breast cancer samples from the TCGA database indicated a high likelihood of co-occurrence of alterations in the *TP53*, *MYC*, *WRN*, *NDRG1*, *NOTCH3*, *UBR5*, and *BRD4* genes, all of which are involved in the above-mentioned interaction network. This finding supports the reliability and robustness of our analysis (Supplementary Figure 4).

## DISCUSSION

Despite recent attempts to understand the clonal evolution of TNBC and to determine a detailed mutational spectrum in these tumors, little is known about the unique mutational profiles and therapeutic targets in TNBC patients from diverse ethnic populations [9]. This study revealed a comprehensive mutational spectrum specific to Korean patients with TNBC, as well as identifying novel, potentially prognostic genes. Compared with a cohort of Western European-North American patients with TNBC, our cohort of Korean patients possessed unique genetic features, which also included commonly mutated genes, such as *TP53* and *PIK3CA* (Supplementary Table 8). Several recent studies have reported that mutations in *NOTCH3* and *NOTCH4* may cause breast cancer [23–25]. Similarly, we discovered novel recurrent SNVs in the N-terminal cytoplasmic domain of NOTCH3, including c.6841C>G (p.A2281P) in seven patients, and in the EGF-like domain of NOTCH4, including c.625T>G (p.T209P) in nine patients, c.118T>G (p.T40P) in five patients, and c.3064C>G (p.A1022P) in four patients. These mutations may have an important role in inducing oncogenic activity by inhibiting the binding of their ligands to NOTCH3 and NOTCH4. In addition, three patients in our cohort had the *PIK3CA* c.3140A>G (p.H1047R) mutation, which was recently reported as being crucial in inducing multipotency and heterogeneity of breast cancer [26, 27]. These findings reinforce the likelihood that the other novel recurrent mutations identified in our cohort warrant further investigation as molecular pathogenic biomarkers.

We also found that 26 (37%) of the 70 patients in our cohort had mutations in *BRCA1* and *BRCA2*, including 12 and 10 patients with homozygous deletions of *BRCA1* and *BRCA2*, respectively, two and four with deleterious somatic mutations, respectively, and three and one with deleterious germline mutations, respectively. In addition, homozygous deletions of DNA damage repair genes, such as *ATM*, *WRN*, and *CHEK2*, were present in more than half of our study cohort, suggesting that a large proportion of Korean patients with TNBC have an impaired DNA repair system, such as a homologous recombination deficiency. These findings suggest that a PARP inhibitor may have potential for treatment of TNBCs.

Adjuvant chemotherapy has been reported to dramatically increase DFS and overall survival of patients with basal-like breast cancer (BLBC) [28]. Of the 70 patients in our TNBC cohort, 67 had been treated with adjuvant chemotherapy. Nevertheless, we found that three homozygously deleted genes were significantly associated with poor prognosis in patients who had received adjuvant chemotherapy. These findings suggest that homozygous deletion of these genes may contribute to resistance to adjuvant chemotherapy. Moreover, our results may provide clues about the mechanism of TNBC resistance to chemotherapy.

## MATERIALS AND METHODS

### Ethics statement

This study was approved by the Institutional Review Board of the Samsung Medical Center, Seoul (South Korea), and performed in accordance with the principles of the Declaration of Helsinki. Because the study was retrospective in nature, the requirement for informed consent was waived. Patient information was anonymized and de-identified prior to analysis.

### Patients and tissue samples

Seventy TNBC and matched normal tissues were collected from the pathology department at Samsung Medical Center, Seoul, South Korea. Immediately upon removal, the specimens had been frozen immediately in liquid nitrogen or fixed in formalin, with the latter used to produce formalin-fixed and paraffin-embedded (FFPE) blocks. Sections of each FFPE sample were stained with hematoxylin and eosin for sample validation by a pathologist (YLC). The expression of ER, PR, and HER2 was assessed by the same pathologist (YLC), as previously described [28].

### Selection of target genes

Of the 368 selected target genes, 234 had previously been reported to be cancer-associated genes frequently mutated in solid tumors and sarcomas, but not in hematological cancers, and listed in the Cancer Gene Census of the Wellcome Trust Sanger Institute (http://cancer.sanger.ac.uk/census/), and 134 were genes encoding cell growth- and kinase-related factors and transcription factors. These 368 genes included 5,700 regions encoding exons. The total size of the target region was 961,497 bp (Supplementary Table 9).

### Targeted exome sequencing using HaloPlex target enrichment

Genomic DNA was extracted from frozen samples using DNeasy Blood & Tissue kits (Qiagen, Hilden, Germany) according to the manufacturer’s instructions. DNA of sufficient purity was defined spectrophotometrically using a 260 nm/280 nm ratio between 1.8–2.1 and a 260 nm/230 nm ratio ≥ 1.5. After digestion and denaturation, targeted fragment DNA was hybridized with biotinylated probes designed to guide circularization of the target DNA fragments, with incorporation of sequencing motifs. Targeted fragments bound to biotinylated HaloPlex probes (Agilent Technologies, Santa Clara, CA, USA) were retrieved using magnetic streptavidin beads. Circularized molecules were closed by ligation, which ensured that only perfectly hybridized fragments were circularized and that only circular DNA targets were amplified by PCR, thus providing enriched and bar-coded amplified products for sequencing with a HiSeq 2000 (Illumina, San Diego, CA, USA).

### Bioinformatic analysis of SNVs and INDELs

Paired-end sequence raw reads were trimmed and filtered to produce clean reads with good base quality (Phred Q score > 20). Burrows-Wheeler Alignment (BWA 0.5.9), the Genome Analysis Toolkit (GATK), and SAMtools were used to align these paired-end sequencing reads with the human reference genome hg19. Identified SNVs and small INDELs were analyzed using the variant databases dbSNP135, dbNSFP COSMIC, and the 1000 Genomes, and several software programs, such as SNPEff, SIFT, PolyPhen2, LRT, PhyloP, Mutation_Taster, Mutation_Assessor, FATHMM, and GERP_NR. Somatic non-synonymous SNVs and INDELs were selected using the following criteria: a >20% read-allele frequency at the position; ≥15 mapped reads at the position; and zero SNV or INDEL allele reads in the targeted sequence of corresponding normal tissue. Variants were confirmed by visualization in the Interactive Genomic Viewer and NextGENe software v2.3.1 (SoftGenetics, State College, PA, USA), as well as by quantitative PCR (qPCR).

### Bioinformatic analysis of CNVs

Genomic CNVs were assessed using NextGENe v2.3.1 (SoftGenetics), which compares the median read coverage levels between target genomic regions of cancer and matched normal tissues after global normalization of genome-wide read coverage levels. CNVs were calculated as the log2 ratio of read coverage in cancer and matched normal tissues. CNVs with a log2 ratio >1.5 were considered amplified, whereas CNVs with a log2 ratio <-1.2 were considered homozygous loss-of-function mutations.

### Survival analysis

Survival was analyzed by the Cox proportional-hazards regression method [29] using clinical information and somatic mutation data of patients who had been treated with adjuvant chemotherapy. After determining the HR and p-value of each mutation, Benjamini-Hochberg multiple testing correction was applied to address the risk of false positives because of multiple analysis (false discovery rate = 0.05) [30].

### Protein–protein interaction networks and gene expression analysis

STRING, KEGG (Kyoto Encyclopedia of Genes and Genomes), and DAVID (Database for Annotation, Visualization, and Integrated Discovery) were used to analyze oncogenic and tumor-suppression pathways in TNBC samples. In addition, CNV information, RNA expression, and mutation data of our TNBC samples were compared with those of TNBC samples from the TCGA database.

### Validation of genomic alterations

Two SNV regions in *TP53* were selected for experimental validation of somatic mutations. Target regions in genomic DNA from tumor and matched normal tissues of patients TNBC030 and TNBC045 were amplified by PCR, and products were either sequenced directly or cloned into the T vector for Sanger sequencing. Five clones from each sample were selected. Frequent CNVs, such as amplification of *NDRG1* and deletion of *ATM*, *BRCA1*, *BRCA2*, and *WRN*, were selected for validation by qPCR. Genomic DNA from tumor and matched normal tissues of patients TNBC038 and TNBC048 for *ATM*; patients TNBC026, TNBC031, TNBC038, and TNBC066 for *BRCA1*; patients TNBC004, TNBC011, TNBC014, and TNBC068 for *BRCA2*; and patient TNBC030 for *WRN* was analyzed by qPCR. The relative expression of these genes in corresponding samples was calculated according to the ddCt method, using *TERT* as a reference gene [31, 32]. Details regarding mutated and altered genomic regions, and the primers used in the validation experiments are provided in Supplementary Table 10.

## SUPPLEMENTARY MATERIALS FIGURES AND TABLES





## Abbreviations

TNBC: triple-negative breast cancer
FFPE: formalin-fixed and paraffin-embedded
ER: estrogen receptor
PR: progesterone receptor
HER2: human epidermal growth factor receptor 2
CNV: copy number variation
BWA: Burrows-Wheeler alignment
GATK: Genome Analysis Toolkit
SNV: single nucleotide variant
qPCR: quantitative PCR
STRING: Search Tool for the Retrieval of Interacting Genes/Proteins
KEGG: Kyoto Encyclopedia of Genes and Genomes
DAVID: Database for Annotation, Visualization, and Integrated Discovery
TCGA: The Cancer Genome Atlas
pT: primary tumor stage
DFS: disease-free survival
DMFS: distant metastasis-free survival
HR: hazard ratio
CI: confidence interval
INDEL: insertion and deletion
F/U: follow-up
B: benign
D: probably damaging
NA: not available
P: possibly damaging
