# Supplementary material for: Targeted exome sequencing of Korean triple-negative breast cancer reveals homozygous deletions associated with poor prognosis of adjuvant chemotherapy-treated patients
Source: Oncotarget. 2017 Jun 27;8(37):61538–50. doi: 10.18632/oncotarget.18618 (PMC5617443; doi:10.18632/oncotarget.18618)
Supplement: Supplementary file 1 [file oncotarget-08-61538-s001.pdf]

Targeted exome sequencing of Korean triple-negative breast cancer reveals homozygous deletions associated with poor prognosis of adjuvant chemotherapy-treated patients

SUPPLEMENTARY MATERIALS

Sample	Targeted NGS	Sanger sequencing					
		PCR product	Cloned DNA				
			#1	#2	#3	#4	#5
TNBC030 (N)	637C	637C	637C	637C	637C	637C	637C
TNBC030 (T)	637C>T	637C>T	637C	637C>T	637C>T	637C>T	637C>T
TNBC045 (N)	578A	578A	578A	578A	578A	578A	578A
TNBC045 (T)	578A>G	578A>G	578A>G	578A>G	578A>G	578A>G	578A>G

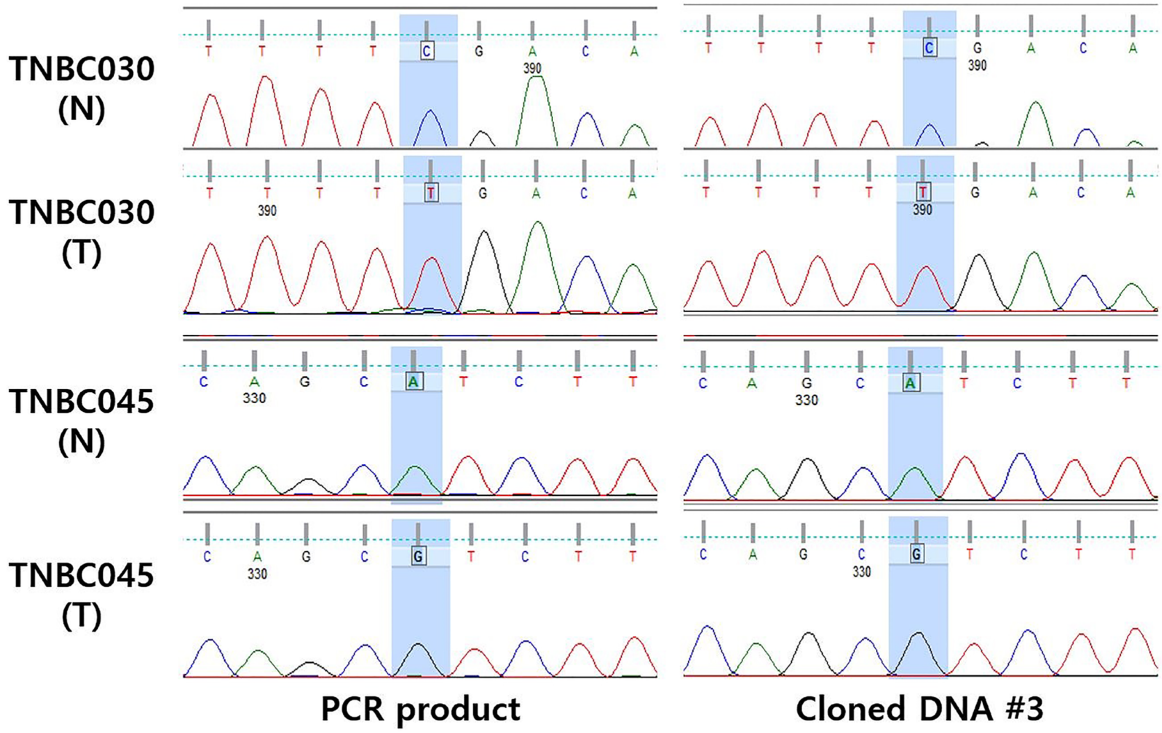

Supplementary Figure 1: Single nucleotide variant (SNV) validation by Sanger sequencing. Experimental validation of targeted exome-sequencing data. Two frequently occurring *TP53* mutations (637C>T and 578A>G) were validated by Sanger sequencing. A single 637C>T clone (clone #1) was identified as wild type, perhaps because of the contamination of the cryopreserved tumor sample.

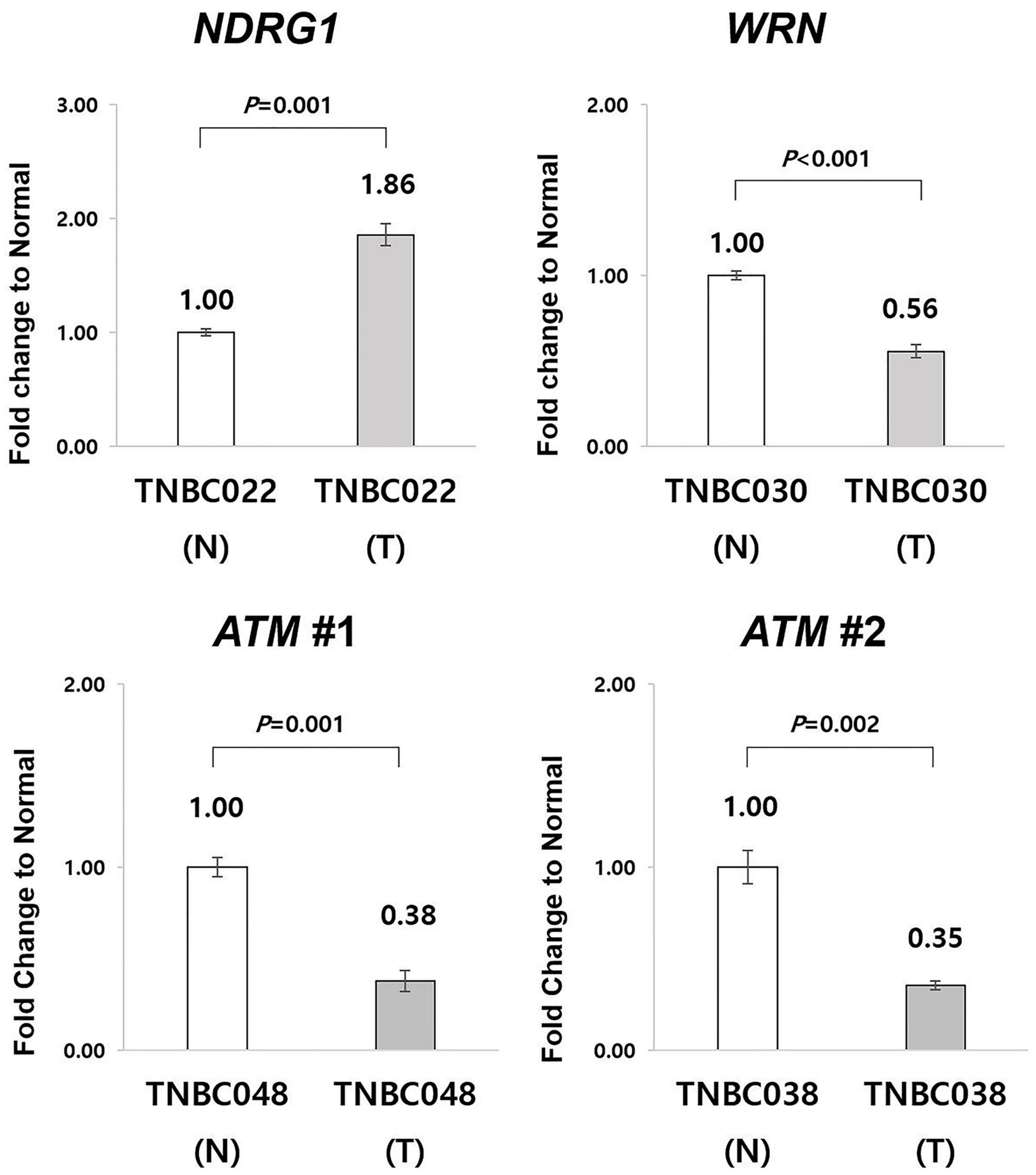

Supplementary Figure 2: Copy number variation (CNV) validation by quantitative PCR (qPCR). Frequently occurring amplifications of *NDRG1* and deletions of *WRN* and *ATM* were validated by qPCR. All experiments were performed in triplicate and demonstrate significant alterations in gene expression in tumor samples.

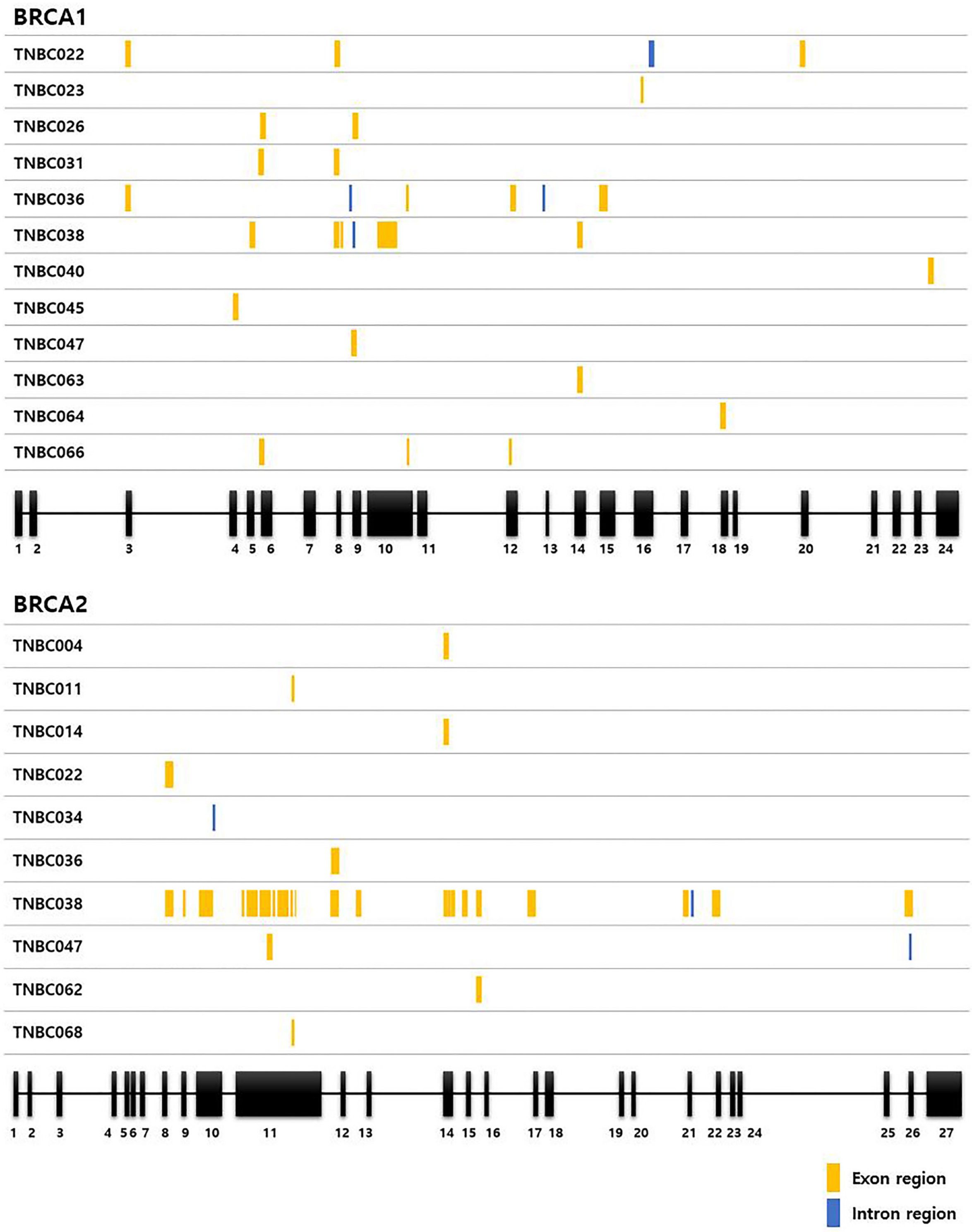

Supplementary Figure 3: Localization of deleted regions of *BRCA1* and *BRCA2* in individual patients. Homozygous deletions of regions of *BRCA1* and *BRCA2* in each corresponding patient are displayed in detail. Deletions in individual patients were observed in a single exon or in multiple exons.

Gene	*TP53*	*UBR5*	*MYC*	*EXT1*	*NDRG1*	*BRD4*	*WRN*	*NOTCH3*	*NOTCH4*
TP53	---	<0.000001	<0.000001	<0.000001	<0.000001	<0.000001	<0.000001	0.000133	0.49133
UBR5		---	<0.000001	<0.000001	<0.000001	0.009468	0.000013	0.039225	0.082807
MYC			---	<0.000001	<0.000001	0.000004	0.016019	0.164945	0.343477
EXT1				---	<0.000001	0.000022	0.051449	0.168537	0.174497
NDRG1					---	0.000016	0.082249	0.199102	0.167127
BRD4						---	0.055443	<0.000001	0.469896
WRN							---	0.432464	0.077401
NOTCH3								---	0.000001
NOTCH4									---

p-values <0.05, as derived via Fisher's Exact test are outlined in red.

p-values are not adjusted for FDR.

Legend
Strong tendency towards mutual exclusivity (0 < Odds Ratio < 0.1)
Some tendency towards mutual exclusivity (0.1 < Odds Ratio <0.5)
No association (0.5 < Odds Ratio < 2)
Tendency toward co-occurrence (2 < Odds Ratio < 10)
Stron tendency towards co-occurrence (Odds Ratio > 10)
No events recorded for one or both genes

Supplementary Figure 4: Mutual exclusivity analysis using The Cancer Genome Atlas (TCGA) breast cancer database. Significantly co-occurring mutations were observed in the DNA damage response-related genes, *TP53*, *MYC*, *WRN*, *NDRG1*, *NOTCH3*, *UBR5*, and *BRD4*.

Supplementary Table 1: Associations between clinicopathological features and disease-free survival (DFS) or distant metastasis-free survival (DMFS)

See Supplementary File: 1

Supplementary Table 2: Targeted exome-sequencing statistics

Target Sequencing Statistics	Tumor Sample	Normal Sample
Target Territory (bp)	2,364,198	2,364,198
Average Target Coverage (X)	130.36	139.71
% of 1x Target Bases	4.25	4.35
% of Target Bases ≥ 2x	93.35	93.12
% of Target Bases ≥ 10x	86.23	86.69
% of Target Bases ≥ 20x	78.47	79.56
% of Target Bases ≥ 30x	71.67	73.13
% of Target Bases ≥ 40x	65.65	67.36
% of Target Bases ≥ 50x	60.33	62.21
% of Target Bases ≥ 100x	40.64	42.57

Target sequencing statistics of 140 samples (70 pairs of tumor and normal samples). The distribution of read coverage depths was similar in tumor and normal samples with average target coverage greater than 130×, which is sufficient for mutation analysis.

Supplementary Table 3: Number of genes with somatic variants or copy number variations (CNVs)

Somatic Variants		Mutation Number	Total	Number of Mutated Genes
Somatic SNVs	Novel	220	292	157
	COSMIC or dbSNP	72		
Somatic Deletions	Novel	11	21	
	COSMIC or dbSNP	10		
Somatic Insertions	Novel	7	9	
	COSMIC or dbSNP	2		
Copy Number Alterations Genes				Number of Altered Genes
Amplification				365
Homozygous deletion				346

Patients with triple-negative breast cancer (TNBC) were found to have 157 mutated genes, 365 amplified genes, and 346 deleted genes. Most of the somatic variants were novel single nucleotide variants (SNVs).

Supplementary Table 4: Complete list of somatic mutations identified in this study, along with their chromosomal positions, frequency, and mutation type

See Supplementary File: 1

Supplementary Table 5: *BRCA1* and *BRCA2* germline mutations

See Supplementary File: 1

Supplementary Table 6: List of all genetically altered genes

See Supplementary File: 1

Supplementary Table 7: Result of Cox proportional hazard ratio analysis

Gene Name	Homozygous Deletion Event	n (%)	Recurrence yes (%)	Recurrence no (%)	DFS HR	DFS low CI	DFS high CI	DFS p-value	DFS Benjamini-Hochberg adjusted p-value
EPHA5	No	59 (88.1)	8 (13.6)	51 (86.4)					
	Yes	8 (11.9)	6 (75.0)	2 (25.0)	7.0154	2.4197	20.340	0.0003	0.0355
MITF	No	61 (91.0)	9 (14.8)	52 (85.2)					
	Yes	6 (8.96)	5 (83.3)	1 (16.7)	9.2915	2.9141	29.625	0.0002	0.0349
ACSL3	No	63 (94.0)	10 (15.9)	53 (84.1)					
	Yes	4 (5.97)	4 (100)	0 (0.00)	7.7495	2.3721	25.317	0.0007	0.0494
Total		67 (100)	14 (20.9)	53 (79.1)					
			**Distant metastasis yes (%)**	**Distant metastasis no (%)**	**DMFS HR**	**DMFS low CI**	**DMFS high CI**	**DMFS p-value**	**DMFS Benjamini-Hochberg adjusted p-value**
MITF	No	61 (91.0)	4 (6.56)	60 (93.4)					
	Yes	6 (8.96)	4 (66.7)	2 (33.3)	26.346	4.796	144.72	0.0002	0.0355
Total		67 (100)	8 (11.9)	59 (88.1)					

Homozygous deletions of three genes were significantly associated with disease-free survival (DFS), and *MITF* was significantly associated with distant metastasis-free survival (DMFS).
CI, confidence interval; DFS, disease-free survival; DMFS, distant metastasis-free survival; HR, hazard ratio.

Supplementary Table 8: Comparisons of frequently altered genes in this cohort of Korean patients with triple-negative breast cancer (TNBC) and Western European-North American (WENA) patients with TNBC

See Supplementary File: 1

Supplementary Table 9: Full list of the 368 target genes analyzed in this study

See Supplementary File: 1

Supplementary Table 10: Validation regions and primer sequences

See Supplementary File: 1
