# Supplementary material for: Targeted exome sequencing of Korean triple-negative breast cancer reveals homozygous deletions associated with poor prognosis of adjuvant chemotherapy-treated patients
Source: Oncotarget. 2017 Jun 27;8(37):61538–50. doi: 10.18632/oncotarget.18618 (PMC5617443; doi:10.18632/oncotarget.18618)
Supplement: Supplementary file 2 [file oncotarget-08-61538-s002.docx]

**Supplementary Table 1: Associations between clinicopathological features and disease-free survival (DFS) or distant metastasis-free survival (DMFS)**

| **Parameter** | **n (%)** | **Recurrence** | | **DFS** | | | | **Distant metastasis** | | **DMFS** | | | | |
| --- | --- | --- | --- | --- | --- | --- | --- | --- | --- | --- | --- | --- | --- | --- |
|  |  | **yes** | **no** | **HR** | **low CI** | **high CI** | **p-value** | **yes** | **no** | **HR** | **low CI** | **high CI** | **p-value** |  |
| Age, yr |  |  |  |  |  |  |  |  |  |  |  |  |  |  |
| (mean ± S.D.) | 48.0±10.4 |  |  |  |  |  |  |  |  |  |  |  |  |  |
| <50 | 39 (55.7) | 9 (23.1) | 30 (76.9) | 1.000 |  |  |  | 5 (12.8) | 4 (87.2) | 1.000 |  |  |  |  |
| ≥50 | 31 (44.3) | 6 (19.4) | 25 (80.6) | 0.845 | 0.301 | 2.375 | 0.750 | 3 (9.7) | 28 (90.3) | 0.788 | 0.188 | 3.300 | 0.744 |  |
| Postmenopause |  |  |  |  |  |  |  |  |  |  |  |  |  |  |
| No | 41 (58.6) | 9 (22.0) | 32 (78.0) | 1.000 |  |  |  | 5 (12.2) | 36 (87.8) | 1.000 |  |  |  |  |
| Yes | 22 (31.4) | 5 (22.7) | 17 (77.3) | 1.144 | 0.380 | 3.443 | 0.811 | 2 (9.1) | 20 (90.9) | 0.908 | 0.174 | 4.744 | 0.909 |  |
| NA | 7 (10.0) | 1 (14.3) | 6 (85.7) |  |  |  |  | 1 (14.3) | 6 (85.7) |  |  |  |  |  |
| pT |  |  |  |  |  |  |  |  |  |  |  |  |  |  |
| 1 | 29 (41.4) | 4 (13.9) | 25 (86.1) | 1.000 |  |  |  | 2 (6.9) | 27 (93.1) | 1.000 |  |  |  |  |
| 2 | 38 (54.3) | 10 (26.3) | 28 (73.7) | 3.025 | 0.828 | 11.05 | 0.094 | 6 (15.8) | 32 (84.2) | 2.819 | 0.563 | 14.11 | 0.207 |  |
| 3 | 3 (4.3) | 1 (33.3) | 2 (66.7) | 6.598 | 0.661 | 65.89 | 0.108 | 0 (0) | 3 (100.0) | NA | NA | NA | NA |  |
| Lymph node metastasis |  |  |  |  |  |  |  |  |  |  |  |  |  |  |
| No | 32 (45.7) | 7 (21.9) | 25 (78.1) | 1.000 |  |  |  | 4 (12.5) | 28 (87.5) | 1.000 |  |  |  |  |
| Yes | 38 (54.3) | 8 (21.1) | 30 (78.9) | 0.822 | 0.298 | 2.271 | 0.706 | 4 (10.6) | 34 (89.4) | 0.979 | 0.243 | 3.935 | 0.976 |  |
| Pathologic stage |  |  |  |  |  |  |  |  |  |  |  |  |  |  |
| I | 14 (20.0) | 3 (21.4) | 11 (78.6) | 1.000 |  |  |  | 1 (7.2) | 13 (92.8) | 1.000 |  |  |  |  |
| II | 44 (62.9) | 9 (20.5) | 35 (79.5) | 1.021 | 0.276 | 3.773 | 0.976 | 6 (13.6) | 38 (86.4) | 2.257 | 0.271 | 18.79 | 0.452 |  |
| III | 12 (17.1) | 3 (25.0) | 9 (75.0) | 1.241 | 0.250 | 6.157 | 0.791 | 1 (8.3) | 11 (91.7) | 1.293 | 0.081 | 20.72 | 0.856 |  |
| Lymphatic invasion |  |  |  |  |  |  |  |  |  |  |  |  |  |  |
| No | 44 (62.9) | 9 (20.5) | 35 (79.5) | 1.000 |  |  |  | 4 (9.1) | 40 (90.9) | 1.000 |  |  |  |  |
| Yes | 26 (37.1) | 6 (23.1) | 20 (76.9) | 1.135 | 0.404 | 3.189 | 0.811 | 4 (15.4) | 22 (84.6) | 1.698 | 0.425 | 6.794 | 0.454 |  |
| Total |  |  |  |  |  |  |  |  |  |  |  |  |  |  |
|  | 70 (100.0) | 15 (21.4) | 55 (78.6) |  |  |  |  | 8 (11.4) | 62 (88.6) |  |  |  |  |  |
| Average F/U |  |  |  |  |  |  |  |  |  |  |  |  |  |  |
| (mean ± S.D.) | 4.88±1.34 |  |  |  |  |  |  |  |  |  |  |  |  |  |

CI, confidence interval; DFS, disease-free survival; DMFS, distant metastasis-free survival; HR, hazard ratio; NA, not available; pT, primary tumor stage.

Advanced primary tumor stage 2 (pT2) was nominally associated with recurrence risk [hazard ratio (HR) = 3.025, *P* = 0.094] and distant metastasis (HR = 2.819, *P* = 0.207), whereas pT3 was nominally associated with risk of recurrence (HR = 6.598, *P* = 0.108).

**Supplementary Table 4: Complete list of somatic mutations identified in this study, along with their chromosomal positions, frequency, and mutation type**

| Chromosomal Position | # | Gene | Chr | Ref | Genotype | Amino Acid Change | Mutation Type | Reported | Mutation Assessment | | | | | | |
| --- | --- | --- | --- | --- | --- | --- | --- | --- | --- | --- | --- | --- | --- | --- | --- |
|  |  |  |  |  |  |  |  |  | SIFT | PolyPhen2 | | LRT | Mutation Taster | Mutation Assessor | |
|  |  |  |  |  |  |  |  |  | score | HDIV pred | HVAR pred | score | score | score |  |
| 32188929 | 9 | *NOTCH4* | 6 | T | TG | 209T>TP | Heterozygous | Novel | 0.010 | D | D | 0.194 | 0.787 | 1.385 |  |
| 41610083 | 7 | *ETV4* | 17 | A | AC | 257V>VG | Heterozygous | Novel | 0.110 | P | P | 0.013 | 0.881 | 1.995 |  |
| 119123138 | 7 | *EXT1* | 8 | T | TG | 50S>SR | Heterozygous | Novel | 0.740 | B | B | 0.003 | 0.379 | 0.000 |  |
| 57429584 | 7 | *GNAS* | 20 | T | TC | 422S>PS | Heterozygous | Novel | 0.180 | B | B | 0.000 | 0.000 | 1.525 |  |
| 15271598 | 7 | *NOTCH3* | 19 | C | CG | 2281A>AP | Heterozygous | Novel | 0.860 | P | B | NA | 0.554 | 0.000 |  |
| 48264069 | 6 | *COL1A1* | 17 | T | TC | 1249E>GE | Heterozygous | Novel | 0.000 | P | B | 0.000 | 0.787 | 3.480 |  |
| 49444984 | 6 | *MLL2* | 12 | G | GC | 828P>AP | Heterozygous | Novel | 0.000 | B | B | NA | NA | 0.550 |  |
| 7573006 | 6 | *TP53* | 17 | T | TG | 368H>HP | Heterozygous | Novel | 0.210 | B | B | 0.452 | 0.086 | 0.345 |  |
| 46245709 | 5 | *ARID2* | 12 | A | AC | 1268N>NT | Heterozygous | Novel | 0.000 | B | B | 0.000 | 0.974 | 0.975 |  |
| 32190819 | 5 | *NOTCH4* | 6 | T | TG | 40T>TP | Heterozygous | Novel | 0.030 | B | B | 0.189 | 0.964 | 2.585 |  |
| 15355153 | 4 | *BRD4* | 19 | T | TG | 824T>TP | Heterozygous | Novel | 0.120 | B | B | 0.148 | 0.001 | -0.690 |  |
| 42005984 | 4 | *GLI3* | 7 | T | TG | 896D>DA | Heterozygous | Novel | 0.000 | D | D | 0.000 | 1.000 | 2.835 |  |
| 42761320 | 4 | *HOOK3* | 8 | A | AC | 21Q>QP | Heterozygous | Novel | 0.070 | D | D | 0.000 | 0.899 | 2.415 |  |
| 28193752 | 4 | *MN1* | 22 | G | AG | 927T>RT | Heterozygous | Novel | 0.150 | D | D | 0.000 | 0.937 | 0.805 |  |
| 11190719 | 4 | *MTOR* | 1 | T | TG | 1827N>NT | Heterozygous | Novel | 0.460 | B | B | 0.023 | 0.017 | 0.345 |  |
| 32171968 | 4 | *NOTCH4* | 6 | C | CG | 1022A>AP | Heterozygous | Novel | NA | D | P | 0.011 | 0.838 | 0.550 |  |
| 113999210 | 4 | *PAX8* | 2 | T | TG | 232H>HP | Heterozygous | Novel | 0.020 | B | B | 0.230 | 0.064 | 0.205 |  |
| 52716019 | 4 | *PPP2R1A* | 19 | T | TG | 195V>VG | Heterozygous | Novel | 0.070 | D | B | 0.000 | 1.000 | 2.960 |  |
| 33613112 | 4 | *TRIM62* | 1 | A | AC | 365I>IS | Heterozygous | Novel | 0.000 | D | D | 0.000 | 0.999 | 2.475 |  |
| 108188238 | 3 | *ATM* | 11 | A | AC | 2113T>TP | Heterozygous | Novel | 0.280 | B | B | 0.650 | 0.002 | 0.000 |  |
| 52440878 | 3 | *BAP1* | 3 | A | AC | 209V>VG | Heterozygous | Novel | 0.000 | D | D | 0.000 | 1.000 | 3.525 |  |
| 149784730 | 3 | *CD74* | 5 | A | AC | 152L>LR | Heterozygous | Novel | 1.000 | P | P | 0.792 | 0.034 | 1.100 |  |
| 36651971 | 3 | *CDKN1A* | 6 | C | AA | 31S>R | Homozygous | dbSNP | 0.990 | B | B | 0.932 | 0.002 | -0.130 |  |
| 53228060 | 3 | *KDM5C* | X | T | TG | 752T>TP | Heterozygous | Novel | 0.170 | B | B | 0.000 | 0.792 | 1.915 |  |
| 43345074 | 3 | *MAP3K14* | 17 | T | TG | 675H>HP | Heterozygous | Novel | 0.000 | D | B | 0.000 | 0.000 | 0.000 |  |
| 1797048 | 3 | *MAPK8IP3* | 16 | T | TC | 255S>PS | Heterozygous | Novel | 0.010 | D | D | 0.000 | 1.000 | 1.895 |  |
| 150551891 | 3 | *MCL1* | 1 | T | TC | 39E>GE | Heterozygous | Novel | 0.540 | B | B | 0.000 | 0.001 | -0.550 |  |
| 28193759 | 3 | *MN1* | 22 | C | CT | 925E>EK | Heterozygous | Novel | 0.290 | D | P | 0.000 | 0.425 | 0.550 |  |
| 15271574 | 3 | *NOTCH3* | 19 | C | GC | 2289A>AP | Heterozygous | dbSNP | 0.370 | B | B | NA | 0.554 | 0.000 |  |
| 113999240 | 3 | *PAX8* | 2 | T | TG | 222H>HP | Heterozygous | Novel | 0.120 | B | B | 0.001 | 0.600 | 1.545 |  |
| 113999171 | 3 | *PAX8* | 2 | T | TG | 245Y>YS | Heterozygous | Novel | 0.030 | B | B | 0.017 | 0.214 | 1.880 |  |
| 178952085 | 3 | *PIK3CA* | 3 | A | AG | 1047H>HR | Heterozygous | dbSNP | 0.160 | P | B | 0.000 | 1.000 | 0.000 |  |
| 178921339 | 3 | *PIK3CA* | 3 | G | GA | 274R>KR | Heterozygous | dbSNP | 0.030 | D | P | 0.000 | 1.000 | 2.175 |  |
| 67569250 | 3 | *PIK3R1* | 5 | G | GC | 123A>PA | Heterozygous | Novel | 0.210 | B | B | 0.001 | 0.900 | 1.355 |  |
| 78896560 | 3 | *RPTOR* | 17 | A | AC | 853T>TP | Heterozygous | Novel | 0.290 | B | B | 0.000 | 0.488 | 1.590 |  |
| 9027387 | 3 | *SRGAP3* | 3 | A | AG | 1039F>FS | Heterozygous | Novel | 0.260 | B | B | 0.000 | 0.819 | 1.750 |  |
| 7577120 | 3 | *TP53* | 17 | C | CT | 273R>LR | Heterozygous | dbSNP | 0.000 | D | D | 0.000 | 1.000 | 3.145 |  |
| 7577538 | 3 | *TP53* | 17 | C | TT | 248R>Q | Homozygous Heterozygous | dbSNP | 0.010 | D | D | 0.000 | 1.000 | 2.970 |  |
| 105246551 | 2 | *AKT1* | 14 | C | CT | 17E>EK | Heterozygous | dbSNP | 0.010 | D | D | 0.000 | 1.000 | 2.175 |  |
| 49464235 | 2 | *BAX* | 19 | T | TC | 180S>PS | Heterozygous | Novel | NA | P | P | NA | 0.003 | 0.000 |  |
| 32937526 | 2 | *BRCA2* | 13 | G | GT | 2729K>NK | Heterozygous | dbSNP | 0.070 | D | D | 0.003 | 0.027 | 1.675 |  |
| 15350304 | 2 | *BRD4* | 19 | T | TC | 1159R>GR | Heterozygous | Novel | 0.040 | P | B | 0.000 | 0.151 | 1.355 |  |
| 21974744 | 2 | *CDKN2A* | 9 | A | AC | 28V>VG | Heterozygous | COSMIC | 0.030 | D | D | NA | 0.238 | 3.785 |  |
| 21974753 | 2 | *CDKN2A* | 9 | A | AC | 25V>VG | Heterozygous | COSMIC | 0.060 | P | B | NA | 0.322 | 2.445 |  |
| 42798092 | 2 | *CIC* | 19 | T | TG | 1349V>VG | Heterozygous | Novel | 0.000 | D | P | NA | 0.856 | 0.550 |  |
| 55266433 | 2 | *EGFR* | 7 | A | AC | 909T>TP | Heterozygous | Novel | 0.000 | D | D | 0.000 | 1.000 | 3.195 |  |
| 66535421 | 2 | *EPHA5* | 4 | G | GC | 14P>AP | Heterozygous | Novel | 0.730 | B | B | 0.274 | 0.001 | 0.000 |  |
| 153588543 | 2 | *FLNA* | X | A | AC | 1207V>VG | Heterozygous | Novel | 0.000 | D | D | 0.000 | 0.972 | 2.640 |  |
| 180057036 | 2 | *FLT4* | 5 | T | TG | 195T>TP | Heterozygous | Novel | 0.170 | D | P | NA | 0.994 | 2.015 |  |
| 138665524 | 2 | *FOXL2* | 3 | G | GC | 14A>GA | Heterozygous | Novel | 0.140 | P | B | 0.120 | 0.858 | 0.000 |  |
| 57429654 | 2 | *GNAS* | 20 | C | CG | 445A>AG | Heterozygous | Novel | 0.320 | P | B | 0.000 | 0.004 | 1.830 |  |
| 48908385 | 2 | *GRIN2D* | 19 | C | CG | 287A>AG | Heterozygous | Novel | 0.030 | B | B | 0.232 | 0.000 | 0.000 |  |
| 59248259 | 2 | *JUN* | 1 | T | TG | 162S>SR | Heterozygous | Novel | 0.200 | B | B | 0.003 | 0.964 | 1.750 |  |
| 40175265 | 2 | *LHFP* | 13 | G | GA | 30P>LP | Heterozygous | Novel | NA | D | D | 0.000 | 1.000 | 2.865 |  |
| 56111729 | 2 | *MAP3K1* | 5 | T | TG | 110V>VG | Heterozygous | Novel | 0.490 | B | B | 0.750 | 0.018 | 0.000 |  |
| 56111749 | 2 | *MAP3K1* | 5 | G | GC | 117A>PA | Heterozygous | Novel | 0.310 | P | B | 0.322 | 0.005 | 0.000 |  |
| 11190717 | 2 | *MTOR* | 1 | C | CG | 1828A>AP | Heterozygous | Novel | 0.150 | B | B | 0.214 | 0.003 | 0.550 |  |
| 40366751 | 2 | *MYCL1* | 1 | C | GC | 149R>RP | Heterozygous | Novel | 0.030 | D | P | 0.300 | 0.689 | 0.460 |  |
| 40366767 | 2 | *MYCL1* | 1 | C | CG | 144A>AP | Heterozygous | Novel | 0.010 | D | P | 0.001 | 0.819 | 1.240 |  |
| 15271583 | 2 | *NOTCH3* | 19 | C | CG | 2286A>AP | Heterozygous | Novel | 0.300 | B | B | NA | 0.554 | 0.000 |  |
| 15271678 | 2 | *NOTCH3* | 19 | T | TG | 2254H>HP | Heterozygous | Novel | 0.260 | B | B | NA | 0.972 | 0.270 |  |
| 164761841 | 2 | *PBX1* | 1 | T | TG | 126S>SA | Heterozygous | Novel | 0.670 | B | B | 0.000 | 0.000 | 0.490 |  |
| 6026864 | 2 | *PMS2* | 7 | G | AA | 511T>M | Homozygous | dbSNP | 0.130 | B | B | 0.125 | 0.001 | 1.040 |  |
| 98231374 | 2 | *PTCH1* | 9 | T | TG | 637T>TP | Heterozygous | Novel | 0.270 | B | B | 0.024 | NA | 0.345 |  |
| 18540099 | 2 | *ROCK1* | 18 | G | GT | 1141Q>KQ | Heterozygous | Novel | 0.460 | B | B | 0.000 | 1.000 | 0.185 |  |
| 117710908 | 2 | *ROS1* | 6 | A | AT | 455L>LX | Heterozygous | Novel | 1.000 | B | B | 0.000 | 1.000 | NA |  |
| 35658179 | 2 | *SFPQ* | 1 | T | TG | 158T>TP | Heterozygous | Novel | 0.240 | B | B | 0.612 | 0.000 | -0.550 |  |
| 9027495 | 2 | *SRGAP3* | 3 | C | CG | 1003G>GA | Heterozygous | Novel | 1.000 | P | B | 0.000 | 0.087 | -0.550 |  |
| 32508304 | 2 | *TIAM1* | 21 | T | TG | 1277N>NT | Heterozygous | Novel | 0.060 | D | P | 0.000 | 0.993 | 2.215 |  |
| 7578190 | 2 | *TP53* | 17 | T | CT | 220Y>CY | Heterozygous | dbSNP | 0.000 | D | D | 0.000 | 1.000 | 3.020 |  |
| 7577100 | 2 | *TP53* | 17 | T | TC | 280R>GR | Heterozygous | COSMIC | 0.000 | D | D | 0.000 | 1.000 | 3.330 |  |
| 7578271 | 2 | *TP53* | 17 | T | CC | 193H>R | Homozygous Heterozygous | COSMIC | 0.010 | D | D | 0.000 | 1.000 | 3.340 |  |
| 31073151 | 2 | *ZNF668* | 16 | A | AC | 389C>CW | Heterozygous | Novel | 0.000 | D | D | 0.000 | 0.979 | 4.715 |  |
| 91643610 | 1 | *AKAP9* | 7 | G | GT | 1194A>SA | Heterozygous | dbSNP | 0.040 | D | D | 0.004 | 0.139 | 2.125 |  |
| 91712512 | 1 | *AKAP9* | 7 | A | AG | 2730Q>QR | Homozygous | dbSNP | 0.320 | B | B | 0.040 | 0.159 | 1.390 |  |
| 91632133 | 1 | *AKAP9* | 7 | C | GC | 968Q>QE | Heterozygous | Novel | 0.070 | B | B | 0.072 | 0.138 | 2.075 |  |
| 29448419 | 1 | *ALK* | 2 | G | GC | 1027P>RP | Heterozygous | dbSNP | NA | B | B | 0.074 | 0.903 | 1.320 |  |
| 56436935 | 1 | *AMFR* | 16 | G | GC | 312H>QH | Heterozygous | Novel | 0.190 | D | P | 0.000 | 1.000 | 2.475 |  |
| 112177629 | 1 | *APC* | 5 | G | GT | 2113S>IS | Heterozygous | Novel | NA | D | P | 0.000 | 0.747 | 1.150 |  |
| 47430289 | 1 | *ARAF* | X | G | delGTGGGCC | FS | Heterozygous | Novel | NA | NA | NA | NA | NA | NA |  |
| 27056196 | 1 | *ARID1A* | 1 | A | AG | 398T>TA | Heterozygous | Novel | 0.520 | B | B | 0.007 | 0.118 | 0.000 |  |
| 46245833 | 1 | *ARID2* | 12 | A | delAG | FS | Heterozygous | Novel | NA | NA | NA | NA | NA | NA |  |
| 108198448 | 1 | *ATM* | 11 | A | AG | 2351E>EG | Heterozygous | Novel | 0.010 | D | D | 0.000 | 0.382 | 2.280 |  |
| 108175523 | 1 | *ATM* | 11 | G | TG | 1873C>FC | Heterozygous | Novel | 0.850 | B | B | 0.000 | 0.851 | 1.430 |  |
| 76909625 | 1 | *ATRX* | X | C | CT | 1427R>RH | Heterozygous | COSMIC | 0.050 | D | D | 0.000 | 0.974 | 1.745 |  |
| 76938193 | 1 | *ATRX* | X | T | TC | 852D>GD | Heterozygous | Novel | 0.010 | D | D | 0.003 | 0.119 | 1.735 |  |
| 76813091 | 1 | *ATRX* | X | T | TA | 2177D>VD | Heterozygous | Novel | 0.000 | D | D | 0.000 | 0.998 | 1.810 |  |
| 63533694 | 1 | *AXIN2* | 17 | A | AC | 487L>LR | Heterozygous | Novel | 0.110 | B | B | 0.747 | 0.997 | 0.000 |  |
| 52438551 | 1 | *BAP1* | 3 | G | TT | 390P>T | Homozygous | COSMIC | 0.010 | B | B | 0.000 | 0.871 | 1.445 |  |
| 52441219 | 1 | *BAP1* | 3 | T | TC | 184D>GD | Heterozygous | Novel | 0.000 | D | D | 0.000 | 1.000 | 3.940 |  |
| 49464220 | 1 | *BAX* | 19 | G | GC | 175A>PA | Heterozygous | Novel | NA | B | B | NA | 0.006 | 0.000 |  |
| 49464203 | 1 | *BAX* | 19 | A | CC | 169H>P | Homozygous | Novel | NA | P | B | NA | 0.000 | 0.000 |  |
| 152771382 | 1 | *BGN* | X | G | GT | 138R>LR | Heterozygous | Novel | 0.100 | B | B | 0.000 | 0.960 | 0.660 |  |
| 91346814 | 1 | *BLM* | 15 | A | AG | 1141N>NS | Heterozygous | Novel | 0.040 | B | B | 0.000 | 0.956 | 2.400 |  |
| 140482939 | 1 | *BRAF* | 7 | G | GC | 399S>CS | Heterozygous | Novel | 0.020 | D | P | 0.000 | NA | 2.080 |  |
| 41245115 | 1 | *BRCA1* | 17 | G | delG | FS | Heterozygous | dbSNP | 0.000 | NA | NA | 0.000 | 0.000 | 0.000 |  |
| 41258531 | 1 | *BRCA1* | 17 | G | GA | 52L>FL | Heterozygous | dbSNP | 0.000 | D | D | 0.000 | 0.903 | 1.785 |  |
| 32968969 | 1 | *BRCA2* | 13 | G | delG | FS | Heterozygous | Novel | 0.000 | NA | NA | 0.000 | 0.000 | 0.000 |  |
| 32914598 | 1 | *BRCA2* | 13 | C | CA | 2036P>TP | Heterozygous | Novel | 0.520 | B | B | 0.972 | 0.017 | 1.320 |  |
| 136913543 | 1 | *BRD3* | 9 | T | TG | 250T>TP | Heterozygous | Novel | 0.070 | D | D | 0.000 | 1.000 | 3.165 |  |
| 136918593 | 1 | *BRD3* | 9 | T | TG | 3T>TP | Heterozygous | Novel | 0.020 | P | P | 0.172 | 0.968 | 2.095 |  |
| 136905231 | 1 | *BRD3* | 9 | A | AC | 523V>VG | Heterozygous | Novel | 0.380 | B | B | 0.674 | 0.161 | 0.000 |  |
| 15376331 | 1 | *BRD4* | 19 | A | AG | 228V>VA | Heterozygous | Novel | 0.540 | B | B | 0.000 | 0.224 | 1.700 |  |
| 15376292 | 1 | *BRD4* | 19 | A | AC | 241V>VG | Heterozygous | Novel | 0.210 | B | B | 0.024 | 0.025 | 1.445 |  |
| 15376313 | 1 | *BRD4* | 19 | A | AG | 234V>VA | Heterozygous | Novel | 0.890 | B | B | 0.010 | 0.001 | 0.460 |  |
| 15376346 | 1 | *BRD4* | 19 | T | TG | 223H>HP | Heterozygous | Novel | 0.380 | B | B | 0.000 | 0.086 | 1.060 |  |
| 40505582 | 1 | *BUB1B* | 15 | T | delTTAT | FS | Heterozygous | Novel | 0.000 | NA | NA | 0.000 | 0.000 | 0.000 |  |
| 61666047 | 1 | *CCDC6* | 10 | A | AC | 46S>SA | Heterozygous | Novel | 0.730 | B | B | 0.229 | 0.218 | -1.100 |  |
| 137621460 | 1 | *CDC25C* | 5 | C | CT | 448R>RQ | Heterozygous | COSMIC | 0.000 | P | B | 0.774 | 0.990 | 2.555 |  |
| 137622920 | 1 | *CDC25C* | 5 | T | TC | 322I>VI | Heterozygous | Novel | 0.500 | B | B | 0.001 | 0.129 | 0.735 |  |
| 12871199 | 1 | *CDKN1B* | 12 | G | insG | FS | Heterozygous | dbSNP | 0.000 | NA | NA | 0.000 | 0.000 | 0.000 |  |
| 29090097 | 1 | *CHEK2* | 22 | G | GT | 505L>IL | Heterozygous | Novel | 0.010 | D | D | 0.000 | 1.000 | 1.425 |  |
| 101989208 | 1 | *CHUK* | 10 | T | TC | 28N>DN | Heterozygous | Novel | 0.040 | P | B | 0.000 | 0.991 | 0.880 |  |
| 42799210 | 1 | *CIC* | 19 | A | AC | 1565D>DA | Heterozygous | Novel | 0.000 | P | B | NA | 0.141 | 0.000 |  |
| 46299533 | 1 | *CREB3L1* | 11 | C | insC | UTR_5_PRIME | Heterozygous | Novel | 0.000 | NA | NA | 0.000 | 0.000 | 0.000 |  |
| 18887992 | 1 | *CRTC1* | 19 | T | TC | 585S>PS | Heterozygous | Novel | 0.010 | D | P | 0.030 | 0.999 | 1.830 |  |
| 91184393 | 1 | *CRTC3* | 15 | A | GA | 538Y>YC | Heterozygous | Novel | 0.240 | D | D | 0.000 | 1.000 | 2.135 |  |
| 91181774 | 1 | *CRTC3* | 15 | A | AC | 455T>TP | Heterozygous | Novel | 0.580 | B | B | 0.915 | 0.002 | -1.320 |  |
| 101892270 | 1 | *CUX1* | 7 | A | AC | 1500H>HP | Heterozygous | Novel | 0.000 | B | B | 0.000 | 0.975 | 1.935 |  |
| 101843359 | 1 | *CUX1* | 7 | A | AC | 668T>TP | Heterozygous | Novel | 0.080 | D | D | 0.000 | 0.986 | 2.390 |  |
| 50828171 | 1 | *CYLD* | 16 | C | TC | 840L>LF | Heterozygous | Novel | 0.010 | D | P | 0.000 | 0.901 | 0.895 |  |
| 33287881 | 1 | *DAXX* | 6 | C | delCCT | In-Frame | Heterozygous | Novel | 0.000 | NA | NA | 0.000 | 0.000 | 0.000 |  |
| 158223442 | 1 | *EBF1* | 5 | T | TA | 274T>ST | Heterozygous | Novel | 0.000 | D | D | 0.000 | 0.991 | 2.420 |  |
| 55211050 | 1 | *EGFR* | 7 | G | GA | 98R>QR | Heterozygous | dbSNP | 0.310 | B | B | 0.192 | 0.006 | 0.600 |  |
| 205592925 | 1 | *ELK4* | 1 | T | TC | 29N>SN | Heterozygous | dbSNP | 0.200 | B | B | 0.001 | 0.996 | 0.865 |  |
| 41572906 | 1 | *EP300* | 22 | C | GC | 1731R>RG | Heterozygous | Novel | 0.050 | D | P | 0.000 | 0.547 | 2.965 |  |
| 66361231 | 1 | *EPHA5* | 4 | G | CG | 314P>RP | Heterozygous | Novel | 0.540 | B | B | 0.002 | 0.629 | 1.830 |  |
| 134967330 | 1 | *EPHB1* | 3 | C | AC | 890T>NT | Heterozygous | Novel | 0.060 | P | B | 0.000 | 1.000 | 1.700 |  |
| 142567977 | 1 | *EPHB6* | 7 | C | CA | 873A>EA | Heterozygous | dbSNP | 0.000 | D | D | 0.000 | 1.000 | 0.875 |  |
| 37868208 | 1 | *ERBB2* | 17 | C | AC | 310S>YS | Heterozygous | COSMIC | 0.000 | D | D | NA | 0.711 | 2.150 |  |
| 37879588 | 1 | *ERBB2* | 17 | A | GG | 655I>V | Homozygous | dbSNP | 0.390 | B | B | NA | 0.684 | 0.750 |  |
| 45858008 | 1 | *ERCC2* | 19 | C | CT | 549V>VM | Heterozygous | Novel | 0.000 | D | P | 0.000 | 1.000 | 2.215 |  |
| 128360431 | 1 | *ETS1* | 11 | G | delG | FS | Heterozygous | Novel | 0.000 | NA | NA | 0.000 | 0.000 | 0.000 |  |
| 41606516 | 1 | *ETV4* | 17 | C | CA | 406G>VG | Heterozygous | dbSNP | 0.050 | D | D | 0.000 | 1.000 | 2.490 |  |
| 41610080 | 1 | *ETV4* | 17 | A | AC | 258V>VG | Heterozygous | Novel | 0.360 | B | B | 0.003 | 0.881 | 1.175 |  |
| 185775016 | 1 | *ETV5* | 3 | G | GC | 353P>AP | Heterozygous | Novel | 0.790 | B | B | 0.000 | NA | 0.550 |  |
| 119122565 | 1 | *EXT1* | 8 | G | GA | 241H>YH | Heterozygous | Novel | 1.000 | P | P | 0.000 | 0.998 | 1.845 |  |
| 119123136 | 1 | *EXT1* | 8 | A | AC | 50S>SR | Heterozygous | Novel | 0.740 | B | B | 0.003 | 0.362 | 0.000 |  |
| 63412697 | 1 | *FAM123B* | X | T | TC | 157E>GE | Heterozygous | Novel | 0.100 | P | P | 0.008 | 0.420 | 1.700 |  |
| 153268219 | 1 | *FBXW7* | 4 | T | TG | 197T>TP | Heterozygous | Novel | 0.340 | D | D | 0.000 | 0.995 | 0.000 |  |
| 123274774 | 1 | *FGFR2* | 10 | A | GG | 383C>R | Homozygous | dbSNP | 0.010 | P | P | 0.000 | 0.999 | 2.475 |  |
| 1803138 | 1 | *FGFR3* | 4 | C | GC | 164L>LV | Heterozygous | Novel | 0.980 | B | B | 0.002 | 0.983 | -0.320 |  |
| 1808870 | 1 | *FGFR3* | 4 | G | GA | 770E>KE | Heterozygous | Novel | 0.030 | D | P | 0.319 | 0.000 | 1.915 |  |
| 1808660 | 1 | *FGFR3* | 4 | A | AC | 760D>DA | Heterozygous | Novel | 0.030 | B | B | NA | 1.000 | 0.080 |  |
| 1808897 | 1 | *FGFR3* | 4 | A | AC | 779T>TP | Heterozygous | Novel | 0.040 | D | P | 0.006 | 0.000 | 1.295 |  |
| 176522551 | 1 | *FGFR4* | 5 | G | GA | 550V>MV | Heterozygous | COSMIC | 0.000 | D | D | 0.000 | NA | -0.795 |  |
| 176524321 | 1 | *FGFR4* | 5 | G | GT | 728A>SA | Heterozygous | Novel | 0.090 | D | D | 0.000 | 0.293 | 0.685 |  |
| 153587625 | 1 | *FLNA* | X | T | delTGGCCACCATAGG | FS | Heterozygous | Novel | 0.000 | NA | NA | 0.000 | 0.000 | 0.000 |  |
| 153596264 | 1 | *FLNA* | X | G | GA | 190R>WR | Heterozygous | Novel | 0.000 | P | B | 0.000 | 0.977 | 1.650 |  |
| 180056358 | 1 | *FLT4* | 5 | T | TG | 296T>TP | Heterozygous | Novel | 0.010 | B | B | NA | 1.000 | 2.515 |  |
| 75746824 | 1 | *FOS* | 14 | T | TG | 129V>VG | Heterozygous | Novel | 0.390 | P | B | 0.455 | 0.544 | 1.725 |  |
| 138665498 | 1 | *FOXL2* | 3 | T | TG | 23T>TP | Heterozygous | Novel | 0.200 | P | B | 0.817 | 0.011 | 0.000 |  |
| 42088125 | 1 | *GLI3* | 7 | A | AG | 215M>MT | Heterozygous | Novel | 0.000 | B | B | 0.000 | 0.958 | 2.110 |  |
| 42007218 | 1 | *GLI3* | 7 | C | CA | 803A>SA | Heterozygous | Novel | 0.600 | B | B | 0.001 | 0.541 | 1.610 |  |
| 42005976 | 1 | *GLI3* | 7 | A | AG | 899S>SP | Heterozygous | Novel | 0.000 | D | D | 0.000 | 1.000 | 2.815 |  |
| 42005973 | 1 | *GLI3* | 7 | T | TG | 900T>TP | Heterozygous | Novel | 0.270 | D | D | 0.000 | 1.000 | 2.015 |  |
| 3119213 | 1 | *GNA11* | 19 | G | GT | 249E>XE | Heterozygous | Novel | 0.040 | P | P | 0.000 | 1.000 | NA |  |
| 57429657 | 1 | *GNAS* | 20 | C | insC | FS | Heterozygous | Novel | 0.000 | NA | NA | 0.000 | 0.000 | 0.000 |  |
| 57429247 | 1 | *GNAS* | 20 | C | CA | 309F>LF | Heterozygous | Novel | 0.720 | B | B | 0.000 | 0.289 | -0.895 |  |
| 57429152 | 1 | *GNAS* | 20 | C | TC | 278P>PS | Heterozygous | Novel | NA | B | B | 0.012 | 0.000 | 0.550 |  |
| 57429683 | 1 | *GNAS* | 20 | T | TC | 455S>PS | Heterozygous | Novel | 0.170 | B | B | 0.070 | 0.282 | 1.040 |  |
| 48946023 | 1 | *GRIN2D* | 19 | A | AG | 947K>KR | Heterozygous | Novel | 0.680 | B | B | 0.138 | 0.001 | 0.000 |  |
| 48945052 | 1 | *GRIN2D* | 19 | C | TT | 760A>V | Homozygous | Novel | 0.000 | D | D | 0.000 | 0.639 | 2.390 |  |
| 146755324 | 1 | *GRM1* | 6 | T | CC | 993S>P | Homozygous | dbSNP | 1.000 | B | B | 0.146 | 0.000 | -0.695 |  |
| 106579343 | 1 | *GUCY1A2* | 11 | A | AC | 629V>VG | Heterozygous | Novel | 0.370 | D | 0.953 | 0.001 | 0.999 | 1.590 |  |
| 240158342 | 1 | *HDAC4* | 2 | T | TG | 14D>DA | Heterozygous | Novel | NA | D | D | 0.005 | 0.707 | 1.100 |  |
| 121437361 | 1 | *HNF1A* | 12 | G | GA | 567V>IV | Heterozygous | Novel | 0.650 | B | B | 0.090 | 0.001 | -0.550 |  |
| 42837779 | 1 | *HOOK3* | 8 | C | CT | 444P>PL | Heterozygous | dbSNP | 0.350 | B | B | 0.000 | 0.993 | -0.080 |  |
| 42761337 | 1 | *HOOK3* | 8 | G | GT | 27A>SA | Heterozygous | Novel | 0.610 | D | P | 0.000 | 0.697 | 1.395 |  |
| 42805539 | 1 | *HOOK3* | 8 | C | CA | 137Q>KQ | Heterozygous | Novel | 0.550 | P | B | 0.000 | 0.508 | 1.720 |  |
| 534288 | 1 | *HRAS* | 11 | C | CT | 12G>GD | Heterozygous | dbSNP | 0.000 | P | B | 0.000 | 1.000 | 3.305 |  |
| 99467198 | 1 | *IGF1R* | 15 | G | CG | 860G>AG | Heterozygous | Novel | 0.010 | D | P | 0.000 | 1.000 | 2.880 |  |
| 107976103 | 1 | *IRS4* | X | C | TC | 1158A>AT | Heterozygous | dbSNP | 0.090 | P | B | 0.031 | 0.019 | 0.805 |  |
| 27934987 | 1 | *JAZF1* | 7 | C | CG | 79K>KN | Heterozygous | Novel | 0.520 | D | D | 0.000 | 0.978 | 1.010 |  |
| 59247751 | 1 | *JUN* | 1 | A | delAATGTTTG | FS | Heterozygous | Novel | 0.000 | NA | NA | 0.000 | 0.000 | 0.000 |  |
| 59248258 | 1 | *JUN* | 1 | C | CT | 162S>SN | Heterozygous | Novel | 0.710 | B | B | 0.003 | 0.683 | 0.365 |  |
| 59248247 | 1 | *JUN* | 1 | C | CG | 166E>EQ | Heterozygous | Novel | 0.060 | D | D | 0.001 | 0.823 | 2.045 |  |
| 53225133 | 1 | *KDM5C* | X | C | CG | 1029A>AP | Heterozygous | Novel | 0.000 | D | D | 0.000 | 0.746 | 2.755 |  |
| 53225102 | 1 | *KDM5C* | X | A | AG | 1039I>IT | Heterozygous | Novel | 0.000 | D | D | 0.000 | 0.189 | 2.620 |  |
| 55964865 | 1 | *KDR* | 4 | C | TC | 791R>RQ | Heterozygous | COSMIC | 0.080 | D | B | 0.000 | 0.997 | 0.825 |  |
| 55973911 | 1 | *KDR* | 4 | C | CG | 469E>EQ | Heterozygous | COSMIC | 0.660 | B | B | 0.006 | 0.000 | 0.205 |  |
| 138602636 | 1 | *KIAA1549* | 7 | G | GA | 579P>LP | Heterozygous | Novel | 0.070 | P | B | 0.614 | NA | 0.805 |  |
| 55570043 | 1 | *KIT* | 4 | A | GA | 304T>TA | Heterozygous | Novel | 0.410 | P | B | 0.000 | 0.754 | 2.540 |  |
| 56086030 | 1 | *KTN1* | 14 | G | CG | 321K>NK | Heterozygous | Novel | 0.300 | P | P | 0.001 | NA | 2.125 |  |
| 47970673 | 1 | *LOC100509575* | X | C | CA | 72R>SR | Heterozygous | Novel | 0.620 | B | B | 0.009 | 0.000 | NA |  |
| 4117466 | 1 | *MAP2K2* | 19 | A | AC | 85V>VG | Heterozygous | Novel | 0.010 | D | D | 0.000 | 1.000 | 2.140 |  |
| 4117419 | 1 | *MAP2K2* | 19 | T | TC | 101K>EK | Heterozygous | Novel | 0.000 | D | P | 0.000 | 1.000 | 3.745 |  |
| 12032537 | 1 | *MAP2K4* | 17 | C | CT | 325P>PS | Heterozygous | Novel | 0.350 | D | P | 0.000 | 1.000 | 0.785 |  |
| 7975919 | 1 | *MAP2K7* | 19 | G | AG | 244V>IV | Heterozygous | Novel | 0.190 | D | P | 0.000 | 0.948 | -0.500 |  |
| 1798684 | 1 | *MAPK8IP3* | 16 | G | GC | 392E>DE | Heterozygous | Novel | 0.880 | B | B | 0.000 | 0.992 | -0.020 |  |
| 116339990 | 1 | *MET* | 7 | A | AG | 284I>IM | Heterozygous | Novel | 0.240 | B | B | 0.794 | 0.000 | 0.000 |  |
| 49445374 | 1 | *MLL2* | 12 | T | CT | 698T>AT | Heterozygous | Novel | 0.000 | B | B | NA | NA | 0.000 |  |
| 49435029 | 1 | *MLL2* | 12 | T | GT | 2175D>DA | Heterozygous | Novel | 0.000 | B | B | 0.004 | NA | 0.000 |  |
| 49425971 | 1 | *MLL2* | 12 | G | GT | 4173P>TP | Heterozygous | Novel | 0.000 | B | B | 0.213 | NA | 0.345 |  |
| 49431177 | 1 | *MLL2* | 12 | C | TT | 3321R>Q | Homozygous | Novel | 0.000 | P | B | 0.007 | NA | 0.690 |  |
| 151927359 | 1 | *MLL3* | 7 | A | delAG | FS | Heterozygous | Novel | 0.000 | NA | NA | 0.000 | 0.000 | 0.000 |  |
| 151945334 | 1 | *MLL3* | 7 | T | CT | 729N>DN | Heterozygous | dbSNP | 0.530 | B | B | 0.269 | 0.007 | 0.895 |  |
| 55532246 | 1 | *MMP2* | 16 | A | AC | 552Y>YS | Heterozygous | Novel | 0.000 | D | D | 0.000 | 1.000 | 2.860 |  |
| 28194208 | 1 | *MN1* | 22 | C | CA | 775S>IS | Heterozygous | Novel | 0.190 | B | B | 0.863 | 0.001 | 0.000 |  |
| 28193737 | 1 | *MN1* | 22 | C | CT | 932G>GD | Heterozygous | Novel | 0.020 | D | P | 0.000 | 0.967 | 0.895 |  |
| 28193757 | 1 | *MN1* | 22 | T | TG | 925E>ED | Heterozygous | Novel | 0.440 | B | B | 0.000 | 0.166 | 0.000 |  |
| 28194182 | 1 | *MN1* | 22 | C | CG | 784A>AP | Heterozygous | Novel | 0.300 | P | B | 0.007 | 0.093 | 0.000 |  |
| 45800146 | 1 | *MUTYH* | 1 | C | CT | 25G>GD | Heterozygous | dbSNP | 0.090 | P | B | 0.937 | 0.009 | 1.445 |  |
| 40366752 | 1 | *MYCL1* | 1 | G | insG | FS | Heterozygous | Novel | 0.000 | NA | NA | 0.000 | 0.000 | 0.000 |  |
| 40363495 | 1 | *MYCL1* | 1 | G | GA | 245A>VA | Heterozygous | Novel | 0.000 | D | D | 0.000 | 1.000 | 2.770 |  |
| 40366703 | 1 | *MYCL1* | 1 | A | AG | 165L>LP | Heterozygous | Novel | 0.130 | B | B | 0.004 | 0.710 | 0.345 |  |
| 16082596 | 1 | *MYCN* | 2 | G | GC | 137G>AG | Heterozygous | Novel | 1.000 | B | B | 0.304 | 0.914 | 1.230 |  |
| 16082590 | 1 | *MYCN* | 2 | A | AG | 135Q>QR | Heterozygous | Novel | 0.780 | D | P | 0.003 | 0.075 | 1.430 |  |
| 24930168 | 1 | *NCOA1* | 2 | C | CG | 610S>SX | Heterozygous | Novel | 0.220 | NA | NA | 0.003 | 1.000 | NA |  |
| 51585072 | 1 | *NCOA4* | 10 | G | GT | 407V>FV | Heterozygous | Novel | NA | P | P | 0.139 | 0.006 | 1.870 |  |
| 51579248 | 1 | *NCOA4* | 10 | G | AG | 52R>QR | Heterozygous | Novel | 0.250 | B | B | 0.003 | 0.072 | 0.345 |  |
| 51586349 | 1 | *NCOA4* | 10 | C | GC | 609L>LV | Heterozygous | Novel | 0.720 | D | D | 0.000 | NA | 2.085 |  |
| 29527568 | 1 | *NF1* | 17 | C | delCT | FS | Heterozygous | Novel | 0.000 | NA | NA | 0.000 | 0.000 | 0.000 |  |
| 29653162 | 1 | *NF1* | 17 | G | TG | 1720E>DE | Heterozygous | Novel | 0.700 | B | B | 0.000 | 0.997 | -0.060 |  |
| 29546110 | 1 | *NF1* | 17 | C | GC | 539P>PA | Heterozygous | Novel | 0.130 | B | B | 0.001 | 0.190 | 0.625 |  |
| 14179771 | 1 | *NFIB* | 9 | G | GT | 191Q>KQ | Heterozygous | Novel | 0.220 | B | B | 0.000 | 0.597 | 1.665 |  |
| 103488163 | 1 | *NFKB1* | 4 | C | CA | 93P>QP | Heterozygous | Novel | 0.130 | P | B | 0.000 | 0.986 | 0.935 |  |
| 36988323 | 1 | *NKX2-1* | 14 | G | GT | 110H>QH | Heterozygous | Novel | 0.290 | D | D | 0.000 | 1.000 | 2.870 |  |
| 70511751 | 1 | *NONO* | X | C | AC | 93L>IL | Heterozygous | Novel | 0.010 | P | P | 0.000 | 1.000 | 1.090 |  |
| 15290911 | 1 | *NOTCH3* | 19 | C | TT | 1100R>H | Homozygous | dbSNP | 0.170 | B | B | 0.645 | 0.999 | 0.565 |  |
| 15295189 | 1 | *NOTCH3* | 19 | T | TG | 828N>NT | Heterozygous | Novel | 0.000 | D | D | 0.003 | 0.999 | 2.940 |  |
| 15271589 | 1 | *NOTCH3* | 19 | T | TG | 2284T>TP | Heterozygous | Novel | 0.280 | B | B | NA | 0.005 | 0.000 |  |
| 15288402 | 1 | *NOTCH3* | 19 | C | CG | 1446C>CS | Heterozygous | Novel | 0.000 | P | P | NA | 1.000 | 2.725 |  |
| 32163395 | 1 | *NOTCH4* | 6 | A | AC | 1944V>VG | Heterozygous | Novel | 0.280 | B | B | 0.442 | 0.000 | 0.000 |  |
| 32188926 | 1 | *NOTCH4* | 6 | A | AG | 210S>SP | Heterozygous | Novel | 0.120 | D | P | 0.019 | 0.695 | 1.765 |  |
| 32188566 | 1 | *NOTCH4* | 6 | T | TG | 297T>TP | Heterozygous | Novel | 0.250 | P | P | 0.069 | 0.787 | 1.525 |  |
| 32183028 | 1 | *NOTCH4* | 6 | T | TG | 666T>TP | Heterozygous | Novel | 0.140 | B | B | 0.010 | 0.161 | -2.735 |  |
| 87339241 | 1 | *NTRK2* | 9 | G | GT | 275D>YD | Heterozygous | Novel | 0.400 | D | D | 0.001 | 0.734 | 1.685 |  |
| 87339142 | 1 | *NTRK2* | 9 | G | GA | 242E>KE | Heterozygous | Novel | 1.000 | B | B | 0.003 | 0.675 | 1.010 |  |
| 31740963 | 1 | *PATZ1* | 22 | T | AT | 209E>VE | Heterozygous | Novel | 0.020 | P | B | 0.000 | 0.973 | 0.550 |  |
| 164761895 | 1 | *PBX1* | 1 | T | TC | 144S>PS | Heterozygous | Novel | 0.010 | P | P | 0.000 | 0.000 | 2.095 |  |
| 41749460 | 1 | *PHOX2B* | 4 | T | AT | 112E>VE | Heterozygous | Novel | 0.000 | D | D | 0.000 | NA | 2.235 |  |
| 178952090 | 1 | *PIK3CA* | 3 | G | GC | 1049G>RG | Heterozygous | dbSNP | 0.150 | B | B | 0.000 | 1.000 | 1.525 |  |
| 178921342 | 1 | *PIK3CA* | 3 | G | GA | 275S>NS | Heterozygous | Novel | 0.630 | B | B | 0.000 | 1.000 | 1.155 |  |
| 67591097 | 1 | *PIK3R1* | 5 | A | AG | 564N>ND | Heterozygous | COSMIC | 0.020 | P | P | 0.000 | 1.000 | 2.720 |  |
| 52714666 | 1 | *PPP2R1A* | 19 | A | AC | 142T>TP | Heterozygous | Novel | 0.020 | B | B | 0.000 | 1.000 | 2.905 |  |
| 48746939 | 1 | *PRKDC* | 8 | T | TC | 2657I>VI | Heterozygous | Novel | 0.050 | B | B | 0.001 | 0.251 | 1.500 |  |
| 48694946 | 1 | *PRKDC* | 8 | T | TG | 3796X>XC | Heterozygous | Novel | 0.000 | D | D | 0.000 | 1.000 | 2.675 |  |
| 98231061 | 1 | *PTCH1* | 9 | G | AG | 741A>VA | Heterozygous | dbSNP | 0.140 | P | P | 0.000 | NA | 2.950 |  |
| 98231100 | 1 | *PTCH1* | 9 | G | GA | 728T>MT | Heterozygous | dbSNP | 0.300 | P | B | 0.000 | 0.101 | 0.550 |  |
| 89720799 | 1 | *PTEN* | 10 | T | delTACT | FS | Heterozygous | COSMIC | 0.000 | NA | NA | 0.000 | 0.000 | 0.000 |  |
| 89692905 | 1 | *PTEN* | 10 | G | AA | 130R>Q | Homozygous | dbSNP | NA | D | D | 0.000 | 1.000 | 3.900 |  |
| 89720653 | 1 | *PTEN* | 10 | C | CA | 268D>ED | Heterozygous | Novel | NA | B | B | 0.000 | 0.998 | -0.630 |  |
| 43610171 | 1 | *RET* | 10 | C | CA | 708A>DA | Heterozygous | Novel | 0.620 | P | B | 0.002 | 0.078 | 0.000 |  |
| 43572740 | 1 | *RET* | 10 | C | CA | 12R>SR | Heterozygous | Novel | 0.590 | B | B | 0.297 | 0.000 | 0.895 |  |
| 183885711 | 1 | *RGL1* | 1 | C | CG | 662S>SC | Heterozygous | Novel | 0.010 | B | B | 0.000 | 1.000 | 1.870 |  |
| 38942435 | 1 | *RICTOR* | 5 | G | GT | 1700P>TP | Heterozygous | Novel | 0.240 | B | B | 0.019 | 0.146 | 0.205 |  |
| 78350342 | 1 | *RNF213* | 17 | C | CT | 4525A>AV | Heterozygous | Novel | 0.480 | B | B | 0.357 | 0.003 | 0.850 |  |
| 20284693 | 1 | *RPS6KA3* | X | C | GG | 20D>H | Homozygous | Novel | 0.100 | B | B | 0.051 | 0.005 | 0.000 |  |
| 64135942 | 1 | *RPS6KA4* | 11 | C | CG | 401D>DE | Heterozygous | Novel | 0.400 | B | B | 0.000 | 0.042 | 1.575 |  |
| 67200279 | 1 | *RPS6KB2* | 11 | A | AT | 196K>KM | Heterozygous | dbSNP | 0.000 | D | D | 0.000 | 0.992 | 4.405 |  |
| 78831617 | 1 | *RPTOR* | 17 | G | AG | 476V>MV | Heterozygous | Novel | 0.070 | D | D | 0.000 | 1.000 | 2.455 |  |
| 17355174 | 1 | *SDHB* | 1 | C | CT | 115R>RQ | Heterozygous | Novel | 0.280 | B | B | 0.035 | 0.176 | 0.205 |  |
| 47125635 | 1 | *SETD2* | 3 | G | GT | 1879R>SR | Heterozygous | Novel | 0.010 | D | D | 0.000 | NA | 1.590 |  |
| 35658265 | 1 | *SFPQ* | 1 | C | CG | 129S>ST | Heterozygous | Novel | 0.130 | B | B | 0.023 | 0.000 | 0.000 |  |
| 35658272 | 1 | *SFPQ* | 1 | C | CG | 127A>AP | Heterozygous | Novel | 0.140 | B | B | 0.475 | 0.033 | 0.550 |  |
| 44198587 | 1 | *SLC29A1* | 6 | C | CT | 243P>PS | Heterozygous | Novel | 0.490 | P | P | 0.071 | 0.924 | 0.930 |  |
| 67473602 | 1 | *SMAD3* | 15 | G | GA | 228E>KE | Heterozygous | Novel | 0.000 | D | D | 0.000 | 1.000 | 2.495 |  |
| 48603046 | 1 | *SMAD4* | 18 | G | delGCAGGCGGCTACT | FS | Heterozygous | COSMIC | 0.000 | NA | NA | 0.000 | 0.000 | 0.000 |  |
| 39283992 | 1 | *SOS1* | 2 | T | TC | 121K>EK | Heterozygous | Novel | 0.000 | P | P | 0.000 | 1.000 | 2.360 |  |
| 39250071 | 1 | *SOS1* | 2 | G | GC | 500Q>EQ | Heterozygous | Novel | 0.900 | B | B | 0.000 | 1.000 | 0.345 |  |
| 36024663 | 1 | *SRC* | 20 | A | AC | 218T>TP | Heterozygous | Novel | 0.000 | D | D | 0.000 | 1.000 | 2.360 |  |
| 9027413 | 1 | *SRGAP3* | 3 | G | insGCT | In-Frame | Heterozygous | Novel | 0.000 | NA | NA | 0.000 | 0.000 | 0.000 |  |
| 9102031 | 1 | *SRGAP3* | 3 | A | AC | 229Y>YD | Heterozygous | Novel | 0.090 | D | P | 0.000 | 0.997 | 2.850 |  |
| 1221319 | 1 | *STK11* | 19 | C | TT | 281P>L | Homozygous | dbSNP | 0.340 | B | B | 0.000 | 0.824 | 0.125 |  |
| 30264420 | 1 | *SUZ12* | 17 | A | AC | 52Y>YS | Heterozygous | Novel | 0.410 | D | D | 0.001 | 0.923 | 0.345 |  |
| 70614084 | 1 | *TAF1* | X | G | GA | 1152R>QR | Heterozygous | COSMIC | 0.030 | P | P | 0.000 | 1.000 | 2.080 |  |
| 57545571 | 1 | *TCF12* | 15 | A | AT | 458S>SC | Heterozygous | Novel | 0.020 | D | D | 0.000 | 1.000 | 2.365 |  |
| 57545590 | 1 | *TCF12* | 15 | A | GG | 464H>R | Homozygous | Novel | 0.090 | D | P | 0.000 | 0.998 | 1.955 |  |
| 48172342 | 1 | *TEC* | 4 | G | GT | 126T>KT | Heterozygous | Novel | 0.830 | B | B | 0.027 | 0.025 | 0.895 |  |
| 1294181 | 1 | *TERT* | 5 | A | AG | 274S>SP | Heterozygous | Novel | 0.340 | B | B | 0.347 | 0.032 | 0.255 |  |
| 48900695 | 1 | *TFE3* | X | G | GC | 20R>GR | Heterozygous | Novel | 0.000 | D | P | 0.002 | 0.029 | 0.000 |  |
| 48900728 | 1 | *TFE3* | X | G | CG | 9R>GR | Heterozygous | Novel | 0.000 | B | B | 0.130 | 0.031 | 0.000 |  |
| 41658758 | 1 | *TFEB* | 6 | A | AG | 65V>VA | Heterozygous | Novel | 0.010 | D | P | 0.000 | 1.000 | 1.500 |  |
| 32508320 | 1 | *TIAM1* | 21 | T | TG | 1272T>TP | Heterozygous | Novel | 0.100 | B | B | 0.000 | 0.462 | 1.735 |  |
| 35698636 | 1 | *TLN1* | 9 | C | CT | 2389W>WX | Heterozygous | Novel | 1.000 | NA | NA | 0.000 | 1.000 | NA |  |
| 120470966 | 1 | *TLR4* | 9 | C | CG | 73S>SR | Heterozygous | dbSNP | 0.340 | D | P | 0.058 | 0.847 | 3.085 |  |
| 120475816 | 1 | *TLR4* | 9 | G | GT | 470L>FL | Heterozygous | dbSNP | 0.010 | D | D | 0.001 | 0.984 | 2.290 |  |
| 7577600 | 1 | *TP53* | 17 | A | insA | FS | Heterozygous | COSMIC | 0.000 | NA | NA | 0.000 | 0.000 | 0.000 |  |
| 7579359 | 1 | *TP53* | 17 | G | insGGAAACCT | FS | Heterozygous | Novel | 0.000 | NA | NA | 0.000 | 0.000 | 0.000 |  |
| 7572988 | 1 | *TP53* | 17 | C | insC | FS | Heterozygous | Novel | 0.000 | NA | NA | 0.000 | 0.000 | 0.000 |  |
| 7578516 | 1 | *TP53* | 17 | G | insG | FS | Heterozygous | Novel | 0.000 | NA | NA | 0.000 | 0.000 | 0.000 |  |
| 7577527 | 1 | *TP53* | 17 | G | delGGATG | FS | Heterozygous | COSMIC | 0.000 | NA | NA | 0.000 | 0.000 | 0.000 |  |
| 7578455 | 1 | *TP53* | 17 | C | delCGCGGA | In-Frame | Heterozygous | COSMIC | 0.000 | NA | NA | 0.000 | 0.000 | 0.000 |  |
| 7577519 | 1 | *TP53* | 17 | G | delGATGGTGAG | In-Frame | Heterozygous | COSMIC | 0.000 | NA | NA | 0.000 | 0.000 | 0.000 |  |
| 7577558 | 1 | *TP53* | 17 | G | delG | FS | Heterozygous | COSMIC | 0.000 | NA | NA | 0.000 | 0.000 | 0.000 |  |
| 7578535 | 1 | *TP53* | 17 | T | delTTG | In-Frame | Heterozygous | COSMIC | 0.000 | NA | NA | 0.000 | 0.000 | 0.000 |  |
| 7574030 | 1 | *TP53* | 17 | G | delG | FS | Heterozygous | COSMIC | 0.000 | NA | NA | 0.000 | 0.000 | 0.000 |  |
| 7577557 | 1 | *TP53* | 17 | A | delAGG | In-Frame | Heterozygous | COSMIC | 0.000 | NA | NA | 0.000 | 0.000 | 0.000 |  |
| 7579329 | 1 | *TP53* | 17 | T | CT | 120K>EK | Heterozygous | dbSNP | 0.000 | D | D | 0.000 | 1.000 | 3.145 |  |
| 7578479 | 1 | *TP53* | 17 | G | GT | 151P>TP | Heterozygous | dbSNP | 0.000 | D | D | 0.000 | 1.000 | 2.855 |  |
| 7577022 | 1 | *TP53* | 17 | G | GA | 306R>XR | Heterozygous | dbSNP | 1.000 | NA | NA | 0.475 | 1.000 | NA |  |
| 7578406 | 1 | *TP53* | 17 | C | CT | 175R>RH | Heterozygous | dbSNP | 0.000 | D | D | 0.000 | 1.000 | 3.345 |  |
| 7577094 | 1 | *TP53* | 17 | G | CG | 282R>GR | Heterozygous | dbSNP | 0.000 | P | P | 0.000 | 1.000 | 3.075 |  |
| 7578395 | 1 | *TP53* | 17 | G | AG | 179H>YH | Heterozygous | COSMIC | 0.000 | D | D | 0.000 | 1.000 | 2.970 |  |
| 7578419 | 1 | *TP53* | 17 | C | CA | 171E>XE | Heterozygous | COSMIC | 0.000 | NA | NA | 0.000 | 1.000 | NA |  |
| 7577141 | 1 | *TP53* | 17 | C | CA | 266G>VG | Heterozygous | COSMIC | 0.000 | D | D | 0.000 | 1.000 | 3.300 |  |
| 7577111 | 1 | *TP53* | 17 | G | TG | 276A>DA | Heterozygous | COSMIC | 0.000 | D | D | 0.000 | 1.000 | 3.055 |  |
| 7577105 | 1 | *TP53* | 17 | G | GC | 278P>RP | Heterozygous | COSMIC | 0.000 | D | D | 0.000 | 1.000 | 3.310 |  |
| 7578403 | 1 | *TP53* | 17 | C | AC | 176C>FC | Heterozygous | COSMIC | 0.000 | D | D | 0.000 | 1.000 | 3.360 |  |
| 7577568 | 1 | *TP53* | 17 | C | CT | 238C>CY | Heterozygous | COSMIC | 0.000 | D | D | 0.000 | 1.000 | 3.365 |  |
| 7578263 | 1 | *TP53* | 17 | G | GA | 196R>XR | Heterozygous | COSMIC | 1.000 | NA | NA | 0.000 | 1.000 | NA |  |
| 7578530 | 1 | *TP53* | 17 | A | GA | 134F>FL | Heterozygous | COSMIC | 0.000 | D | D | 0.000 | 1.000 | 2.645 |  |
| 7578268 | 1 | *TP53* | 17 | A | CA | 194L>LR | Heterozygous | COSMIC | 0.000 | D | D | 0.000 | 1.000 | 3.115 |  |
| 7577517 | 1 | *TP53* | 17 | A | AC | 255I>IS | Heterozygous | COSMIC | 0.000 | D | D | 0.000 | 1.000 | 2.250 |  |
| 7578262 | 1 | *TP53* | 17 | C | CG | 196R>RP | Heterozygous | COSMIC | 0.000 | D | D | 0.000 | 1.000 | 3.250 |  |
| 7574003 | 1 | *TP53* | 17 | G | AG | 342R>XR | Heterozygous | COSMIC | 0.730 | NA | NA | 0.002 | 1.000 | NA |  |
| 7577056 | 1 | *TP53* | 17 | C | CA | 294E>DE | Heterozygous | COSMIC | 0.430 | B | B | 0.389 | 0.452 | 2.330 |  |
| 7577130 | 1 | *TP53* | 17 | A | GG | 270F>L | Homozygous | COSMIC | 0.000 | D | D | 0.000 | 1.000 | 1.550 |  |
| 7578212 | 1 | *TP53* | 17 | G | AA | 213R>X | Homozygous | COSMIC | 1.000 | NA | NA | 0.000 | 1.000 | NA |  |
| 7572991 | 1 | *TP53* | 17 | T | TC | 373K>RK | Heterozygous | Novel | 0.220 | B | 0.037 | 0.026 | 0.915 | 1.350 |  |
| 186324603 | 1 | *TPR* | 1 | C | CG | 704V>VL | Heterozygous | Novel | 0.550 | B | B | 0.001 | 0.812 | 0.720 |  |
| 28891116 | 1 | *TRIM27* | 6 | C | CG | 98K>KN | Heterozygous | Novel | 0.080 | P | B | 0.000 | 0.154 | 2.000 |  |
| 115053250 | 1 | *TRIM33* | 1 | A | delAGA | In-Frame | Heterozygous | Novel | 0.000 | NA | NA | 0.000 | 0.000 | 0.000 |  |
| 114940565 | 1 | *TRIM33* | 1 | C | CG | 1056L>LF | Heterozygous | Novel | 0.020 | D | P | 0.004 | NA | 0.345 |  |
| 33646759 | 1 | *TRIM62* | 1 | G | AG | 92P>LP | Heterozygous | Novel | 0.680 | P | P | 0.000 | 0.982 | -1.005 |  |
| 33613115 | 1 | *TRIM62* | 1 | A | AC | 364V>VG | Heterozygous | Novel | 0.430 | P | B | 0.000 | 0.880 | 0.615 |  |
| 2121885 | 1 | *TSC2* | 16 | T | TG | 683S>SA | Heterozygous | Novel | 0.510 | B | B | 0.556 | 0.000 | 0.000 |  |
| 5036274 | 1 | *USP6* | 17 | C | CA | 89H>NH | Heterozygous | dbSNP | 0.430 | B | B | 0.000 | 0.000 | -1.850 |  |
| 5071261 | 1 | *USP6* | 17 | G | AA | 1024R>Q | Homozygous | Novel | 0.510 | D | D | 0.000 | 0.990 | 1.230 |  |
| 30938704 | 1 | *WRN* | 8 | G | AG | 387M>IM | Heterozygous | dbSNP | 0.360 | B | B | 0.055 | 0.001 | 1.150 |  |
| 14199844 | 1 | *XPC* | 3 | C | CG | 513M>MI | Heterozygous | dbSNP | 0.400 | B | B | 0.009 | 0.001 | -1.575 |  |

#, number of samples; Chr, chromosome; Ref, reference nucleotide; B, benign; P, possibly damaging; D, probably damaging; NA, not available.

**Supplementary Table 5: *BRCA1* and *BRCA2* germline mutations**

| Chromosomal Position | # | Gene | Chr | Ref | Genotype | Amino Acid Change | Mutation Type | Reported | Mutation Assessment | | | | | |
| --- | --- | --- | --- | --- | --- | --- | --- | --- | --- | --- | --- | --- | --- | --- |
|  |  |  |  |  |  |  |  |  | SIFT | PolyPhen2 | | LRT | Mutation Taster | Mutation Assessor |
|  |  |  |  |  |  |  |  |  | score | HDIV pred | HVAR pred | score | score | score |
| 41244000 | 40 | *BRCA1* | 17 | T | CC;TC | 1183K>RK | Homozygous Heterozygous | dbSNP | 1.000 | B | B | 0.152 | 0.000 | -1.085 |
| 41244936 | 40 | *BRCA1* | 17 | G | AA;GA | 871P>LP | Homozygous Heterozygous | dbSNP | 1.000 | B | B | 0.001 | 0.000 | -3.395 |
| 41246626 | 2 | *BRCA1* | 17 | T | TA | 308S>CS | Heterozygous | dbSNP | 0.020 | D | D | 0.000 | NA | 3.935 |
| 41246624 | 2 | *BRCA1* | 17 | G | delGC | FS | Homozygous | Novel | 0.000 | NA | NA | 0.000 | 0.000 | 0.000 |
| 41201205 | 1 | *BRCA1* | 17 | A | GA | 1801L>LP | Heterozygous | dbSNP | 0.000 | D | D | 0.002 | 0.804 | 2.175 |
| 41244982 | 1 | *BRCA1* | 17 | A | AG | 856Y>YH | Heterozygous | dbSNP | 0.010 | P | P | 0.095 | 0.338 | 2.755 |
| 41256907 | 1 | *BRCA1* | 17 | A | delAAAinsGG | FS | Homozygous | Novel | 0.000 | NA | NA | 0.000 | 0.000 | 0.000 |
| 41219680 | 1 | *BRCA1* | 17 | G | delGTG | In-Frame | Homozygous | dbSNP | 0.000 | NA | NA | 0.000 | 0.000 | 0.000 |
| 32906729 | 31 | *BRCA2* | 13 | A | AC;CC | 372N>NH | Homozygous Heterozygous | dbSNP | 0.110 | B | B | 0.539 | 0.005 | NA |
| 32906480 | 15 | *BRCA2* | 13 | A | CA | 289N>NH | Heterozygous | dbSNP | 0.060 | B | B | 0.395 | 0.084 | 1.445 |
| 32911463 | 12 | *BRCA2* | 13 | A | GA;GG | 991N>ND | Homozygous Heterozygous | dbSNP | 1.000 | B | B | 0.082 | 0.000 | -1.150 |
| 32972884 | 4 | *BRCA2* | 13 | A | AG | 3412I>IV | Heterozygous | dbSNP | 0.600 | B | B | 0.039 | 0.000 | 0.000 |
| 32930598 | 1 | *BRCA2* | 13 | T | TC | 2490I>TI | Heterozygous | dbSNP | 0.260 | B | B | 0.327 | 0.005 | 0.550 |
| 32944570 | 1 | *BRCA2* | 13 | G | AG | 2788W>XW | Heterozygous | dbSNP | 1.000 | NA | NA | 0.000 | 1.000 | NA |
| 32906558 | 1 | *BRCA2* | 13 | T | TA | 315C>SC | Heterozygous | dbSNP | 0.480 | B | B | 0.988 | 0.001 | 1.040 |
| 32906769 | 1 | *BRCA2* | 13 | A | AG | 385K>KR | Heterozygous | Novel | 0.310 | B | B | 0.303 | 0.002 | 1.040 |
| 32907359 | 1 | *BRCA2* | 13 | A | AC | 582T>TP | Heterozygous | dbSNP | 0.310 | D | P | 0.310 | 0.026 | 1.905 |

#, number of samples; Chr, chromosome; Ref, reference nucleotide; B, benign; P, possibly damaging; D, probably damaging; NA, not available.

Evaluation of patients with triple-negative breast cancer (TNBC) identified two novel germline mutations in *BRCA1* and one previously reported germline mutation in *BRCA2.*

**Supplementary Table 6: List of all genetically altered genes**

| **Somatically Mutated Genes** | | | **Amplified Genes** | | | **Homozygously Deleted Genes** | | |
| --- | --- | --- | --- | --- | --- | --- | --- | --- |
| **Gene** | **Frequency** | **(%)** | **Gene** | **Frequency** | **(%)** | **Gene** | **Frequency** | **(%)** |
| *TP53* | 45 | 64 | *NDRG1* | 36 | 51 | *WRN* | 30 | 43 |
| *NOTCH4* | 19 | 27 | *UBR5* | 32 | 46 | *IL6ST* | 22 | 31 |
| *NOTCH3* | 14 | 20 | *PTK2* | 32 | 46 | *APC* | 21 | 30 |
| *GNAS* | 12 | 17 | *RECQL4* | 26 | 37 | *PTK2B* | 20 | 29 |
| *BRD4* | 10 | 14 | *MYC* | 26 | 37 | *NF1* | 19 | 27 |
| *MN1* | 10 | 14 | *IKBKE* | 25 | 36 | *SETD2* | 18 | 26 |
| *MLL2* | 9 | 13 | *EXT1* | 25 | 36 | *PTPRD* | 17 | 24 |
| *PAX8* | 9 | 13 | *CDK2* | 24 | 34 | *PBRM1* | 17 | 24 |
| *EXT1* | 8 | 11 | *NTRK1* | 24 | 34 | *MLL3* | 16 | 23 |
| *PIK3CA* | 8 | 11 | *DDR2* | 22 | 31 | *PCM1* | 16 | 23 |
| *ETV4* | 7 | 10 | *MCL1* | 22 | 31 | *PLD2* | 15 | 21 |
| *GLI3* | 7 | 10 | *TPR* | 20 | 29 | *PIK3R1* | 15 | 21 |
| *HOOK3* | 7 | 10 | *PARP1* | 19 | 27 | *CDK2* | 14 | 20 |
| *MYCL1* | 7 | 10 | *TPM3* | 19 | 27 | *CSF1R* | 14 | 20 |
| *SRGAP3* | 7 | 10 | *PRCC* | 19 | 27 | *BUB1B* | 14 | 20 |
| *ARID2* | 6 | 9 | *RNF213* | 19 | 27 | *CDK12* | 14 | 20 |
| *COL1A1* | 6 | 9 | *ERC1* | 19 | 27 | *MTOR* | 13 | 19 |
| *MTOR* | 6 | 9 | *FH* | 18 | 26 | *CHEK2* | 13 | 19 |
| *TRIM62* | 6 | 9 | *NBN* | 18 | 26 | *ATM* | 13 | 19 |
| *ATM* | 5 | 7 | *RGL1* | 17 | 24 | *RB1* | 13 | 19 |
| *BAP1* | 5 | 7 | *PTPRD* | 16 | 23 | *MAP3K1* | 13 | 19 |
| *JUN* | 5 | 7 | *TIAM1* | 16 | 23 | *TIAM1* | 12 | 17 |
| *KDM5C* | 5 | 7 | *NOTCH4* | 16 | 23 | *ERCC2* | 12 | 17 |
| *PPP2R1A* | 5 | 7 | *IGF1R* | 16 | 23 | *KTN1* | 12 | 17 |
| *BRCA2* | 4 | 6 | *IKBKB* | 16 | 23 | *BRCA1* | 12 | 17 |
| *CDKN2A* | 4 | 6 | *GATA3* | 16 | 23 | *TSHR* | 12 | 17 |
| *FGFR3* | 4 | 6 | *PBX1* | 16 | 23 | *MLL2* | 11 | 16 |
| *GRIN2D* | 4 | 6 | *MLL2* | 15 | 21 | *PRKDC* | 11 | 16 |
| *MAP3K1* | 4 | 6 | *FLT4* | 15 | 21 | *TCF4* | 11 | 16 |
| *MAPK8IP3* | 4 | 6 | *EGFR* | 15 | 21 | *USP6* | 11 | 16 |
| *PIK3R1* | 4 | 6 | *RPTOR* | 15 | 21 | *RPS6KA2* | 11 | 16 |
| *PTCH1* | 4 | 6 | *RUNX1T1* | 15 | 21 | *TAF1* | 11 | 16 |
| *RPTOR* | 4 | 6 | *COX6C* | 15 | 21 | *KIT* | 11 | 16 |
| *SFPQ* | 4 | 6 | *FLNA* | 14 | 20 | *MAP2K2* | 11 | 16 |
| *AKAP9* | 3 | 4 | *TSC2* | 14 | 20 | *EML4* | 11 | 16 |
| *ATRX* | 3 | 4 | *ATR* | 14 | 20 | *RPS6KA3* | 11 | 16 |
| *BAX* | 3 | 4 | *MAML2* | 14 | 20 | *GNAQ* | 11 | 16 |
| *BRD3* | 3 | 4 | *NTRK3* | 14 | 20 | *KIAA1549* | 10 | 14 |
| *CD74* | 3 | 4 | *CRTC3* | 14 | 20 | *PMS1* | 10 | 14 |
| *CDKN1A* | 3 | 4 | *TFEB* | 14 | 20 | *BRCA2* | 10 | 14 |
| *CIC* | 3 | 4 | *MLL3* | 13 | 19 | *CHUK* | 10 | 14 |
| *EGFR* | 3 | 4 | *ERCC2* | 13 | 19 | *ALDH2* | 10 | 14 |
| *EPHA5* | 3 | 4 | *SMARCA4* | 13 | 19 | *FGFR3* | 10 | 14 |
| *FLNA* | 3 | 4 | *EP300* | 13 | 19 | *TP53* | 10 | 14 |
| *FLT4* | 3 | 4 | *CUX1* | 13 | 19 | *CDC25C* | 10 | 14 |
| *FOXL2* | 3 | 4 | *PRKDC* | 12 | 17 | *PLK2* | 10 | 14 |
| *MAP3K14* | 3 | 4 | *KIAA1549* | 12 | 17 | *FLT4* | 9 | 13 |
| *MCL1* | 3 | 4 | *BRD4* | 12 | 17 | *EGFR* | 9 | 13 |
| *NF1* | 3 | 4 | *RELA* | 12 | 17 | *SMARCA4* | 9 | 13 |
| *PBX1* | 3 | 4 | *ALK* | 12 | 17 | *EP300* | 9 | 13 |
| *PTEN* | 3 | 4 | *RET* | 12 | 17 | *HDAC4* | 9 | 13 |
| *TIAM1* | 3 | 4 | *MAP4K1* | 12 | 17 | *GLI1* | 9 | 13 |
| *AKT1* | 2 | 3 | *GNAS* | 12 | 17 | *MLH1* | 9 | 13 |
| *BRCA1* | 2 | 3 | *TERT* | 12 | 17 | *CDKN2A* | 9 | 13 |
| *CRTC3* | 2 | 3 | *CRTC1* | 12 | 17 | *EPHA5* | 9 | 13 |
| *CUX1* | 2 | 3 | *AKT2* | 12 | 17 | *KDM6A* | 9 | 13 |
| *ERBB2* | 2 | 3 | *AXIN2* | 12 | 17 | *TCF12* | 9 | 13 |
| *FGFR4* | 2 | 3 | *SLC45A3* | 12 | 17 | *PRKD1* | 9 | 13 |
| *KDR* | 2 | 3 | *PRKCA* | 12 | 17 | *CTNNA1* | 9 | 13 |
| *LHFP* | 2 | 3 | *ETV6* | 12 | 17 | *SMAD4* | 9 | 13 |
| *MAP2K2* | 2 | 3 | *TCF4* | 11 | 16 | *FLNA* | 8 | 11 |
| *MLL3* | 2 | 3 | *SMARCB1* | 11 | 16 | *TSC2* | 8 | 11 |
| *MYCN* | 2 | 3 | *KDM5C* | 11 | 16 | *PAX7* | 8 | 11 |
| *NCOA4* | 2 | 3 | *EPHB6* | 11 | 16 | *PLCG2* | 8 | 11 |
| *NTRK2* | 2 | 3 | *EPHA3* | 11 | 16 | *ERBB4* | 8 | 11 |
| *PMS2* | 2 | 3 | *EEF2K* | 11 | 16 | *MAP3K6* | 8 | 11 |
| *PRKDC* | 2 | 3 | *STAT1* | 11 | 16 | *SOS1* | 8 | 11 |
| *RET* | 2 | 3 | *SMO* | 11 | 16 | *CDH1* | 8 | 11 |
| *ROCK1* | 2 | 3 | *SDHC* | 11 | 16 | *PDGFRA* | 8 | 11 |
| *ROS1* | 2 | 3 | *WRN* | 10 | 14 | *TCF7L2* | 8 | 11 |
| *SOS1* | 2 | 3 | *PTK2B* | 10 | 14 | *LIFR* | 8 | 11 |
| *TCF12* | 2 | 3 | *MTOR* | 10 | 14 | *ATRX* | 8 | 11 |
| *TLR4* | 2 | 3 | *CHEK2* | 10 | 14 | *CSNK1A1* | 8 | 11 |
| *TRIM33* | 2 | 3 | *PMS1* | 10 | 14 | *HSP90AA1* | 8 | 11 |
| *USP6* | 2 | 3 | *PAX7* | 10 | 14 | *CHEK1* | 8 | 11 |
| *ZNF668* | 2 | 3 | *MSH6* | 10 | 14 | *GAB1* | 8 | 11 |
| *ALK* | 1 | 1 | *FLT1* | 10 | 14 | *ARID2* | 8 | 11 |
| *AMFR* | 1 | 1 | *NOTCH3* | 10 | 14 | *RPTOR* | 7 | 10 |
| *APC* | 1 | 1 | *NFKB1* | 10 | 14 | *CUX1* | 7 | 10 |
| *ARAF* | 1 | 1 | *HOOK3* | 10 | 14 | *BRD4* | 7 | 10 |
| *ARID1A* | 1 | 1 | *POU5F1* | 10 | 14 | *RELA* | 7 | 10 |
| *AXIN2* | 1 | 1 | *FUBP1* | 10 | 14 | *MSH6* | 7 | 10 |
| *BGN* | 1 | 1 | *MAP2K7* | 10 | 14 | *PTCH1* | 7 | 10 |
| *BLM* | 1 | 1 | *SRGAP3* | 10 | 14 | *MSH2* | 7 | 10 |
| *BRAF* | 1 | 1 | *NF2* | 10 | 14 | *CDKN1B* | 7 | 10 |
| *BUB1B* | 1 | 1 | *CSNK1D* | 10 | 14 | *STK11* | 7 | 10 |
| *CCDC6* | 1 | 1 | *RPS6KB2* | 10 | 14 | *TBX22* | 7 | 10 |
| *CDC25C* | 1 | 1 | *DDB2* | 10 | 14 | *FGFR4* | 7 | 10 |
| *CDKN1B* | 1 | 1 | *CREB3L1* | 10 | 14 | *BRD3* | 7 | 10 |
| *CHEK2* | 1 | 1 | *MDM4* | 10 | 14 | *EBF1* | 7 | 10 |
| *CHUK* | 1 | 1 | *SLC29A1* | 10 | 14 | *ETS1* | 7 | 10 |
| *CREB3L1* | 1 | 1 | *USP6* | 9 | 13 | *FLCN* | 7 | 10 |
| *CRTC1* | 1 | 1 | *RPS6KA2* | 9 | 13 | *ROS1* | 7 | 10 |
| *CYLD* | 1 | 1 | *PTCH1* | 9 | 13 | *SDHB* | 7 | 10 |
| *DAXX* | 1 | 1 | *RICTOR* | 9 | 13 | *FBXW7* | 7 | 10 |
| *EBF1* | 1 | 1 | *MTUS2* | 9 | 13 | *NR4A3* | 7 | 10 |
| *ELK4* | 1 | 1 | *AKAP12* | 9 | 13 | *DICER1* | 7 | 10 |
| *EP300* | 1 | 1 | *NFIB* | 9 | 13 | *RNF213* | 6 | 9 |
| *EPHB1* | 1 | 1 | *AKAP9* | 9 | 13 | *NOTCH4* | 6 | 9 |
| *EPHB6* | 1 | 1 | *ASPSCR1* | 9 | 13 | *IGF1R* | 6 | 9 |
| *ERCC2* | 1 | 1 | *PAX8* | 9 | 13 | *ATR* | 6 | 9 |
| *ETS1* | 1 | 1 | *BGN* | 9 | 13 | *ALK* | 6 | 9 |
| *ETV5* | 1 | 1 | *TMPRSS2* | 9 | 13 | *RET* | 6 | 9 |
| *FAM123B* | 1 | 1 | *MAPK8IP3* | 9 | 13 | *MAP4K1* | 6 | 9 |
| *FBXW7* | 1 | 1 | *ERCC4* | 9 | 13 | *GNAS* | 6 | 9 |
| *FGFR2* | 1 | 1 | *CDC73* | 9 | 13 | *SMARCB1* | 6 | 9 |
| *FOS* | 1 | 1 | *PAX3* | 9 | 13 | *KDM5C* | 6 | 9 |
| *GNA11* | 1 | 1 | *RAF1* | 9 | 13 | *FLT1* | 6 | 9 |
| *GRM1* | 1 | 1 | *THOC5* | 9 | 13 | *NOTCH3* | 6 | 9 |
| *GUCY1A2* | 1 | 1 | *PIK3CA* | 9 | 13 | *NFKB1* | 6 | 9 |
| *HDAC4* | 1 | 1 | *TRIM27* | 9 | 13 | *RICTOR* | 6 | 9 |
| *HNF1A* | 1 | 1 | *MAPK13* | 9 | 13 | *MTUS2* | 6 | 9 |
| *HRAS* | 1 | 1 | *NCOA1* | 9 | 13 | *AKAP12* | 6 | 9 |
| *IGF1R* | 1 | 1 | *AKT3* | 9 | 13 | *NFIB* | 6 | 9 |
| *IRS4* | 1 | 1 | *ETV5* | 9 | 13 | *KDR* | 6 | 9 |
| *JAZF1* | 1 | 1 | *ATM* | 8 | 11 | *COL1A1* | 6 | 9 |
| *KIAA1549* | 1 | 1 | *HDAC4* | 8 | 11 | *HNF1A* | 6 | 9 |
| *KIT* | 1 | 1 | *PLCG2* | 8 | 11 | *MITF* | 6 | 9 |
| *KTN1* | 1 | 1 | *ERBB4* | 8 | 11 | *TRIM33* | 6 | 9 |
| *LOC100509575* | 1 | 1 | *MSH2* | 8 | 11 | *BAX* | 6 | 9 |
| *MAP2K4* | 1 | 1 | *CDKN1B* | 8 | 11 | *BIRC7* | 6 | 9 |
| *MAP2K7* | 1 | 1 | *GLI3* | 8 | 11 | *SUFU* | 6 | 9 |
| *MET* | 1 | 1 | *GRB10* | 8 | 11 | *PRUNE2* | 6 | 9 |
| *MMP2* | 1 | 1 | *GRIN2D* | 8 | 11 | *BAP1* | 6 | 9 |
| *MUTYH* | 1 | 1 | *ARAF* | 8 | 11 | *CD74* | 6 | 9 |
| *NCOA1* | 1 | 1 | *ERBB2* | 8 | 11 | *CTNNB1* | 6 | 9 |
| *NFIB* | 1 | 1 | *MYB* | 8 | 11 | *BRIP1* | 6 | 9 |
| *NFKB1* | 1 | 1 | *C11orf10* | 8 | 11 | *DPYD* | 6 | 9 |
| *NKX2-1* | 1 | 1 | *ETV1* | 8 | 11 | *PMS2* | 6 | 9 |
| *NONO* | 1 | 1 | *IDH2* | 8 | 11 | *BIVM-ERCC5* | 6 | 9 |
| *PATZ1* | 1 | 1 | *EPHB1* | 8 | 11 | *WIF1* | 6 | 9 |
| *PHOX2B* | 1 | 1 | *CANT1* | 8 | 11 | *TSC1* | 6 | 9 |
| *RGL1* | 1 | 1 | *CSF1R* | 7 | 10 | *MRE11A* | 6 | 9 |
| *RICTOR* | 1 | 1 | *BRCA2* | 7 | 10 | *FLI1* | 6 | 9 |
| *RNF213* | 1 | 1 | *GLI1* | 7 | 10 | *TAF15* | 6 | 9 |
| *RPS6KA3* | 1 | 1 | *MAP3K6* | 7 | 10 | *MAP2K4* | 6 | 9 |
| *RPS6KA4* | 1 | 1 | *STK11* | 7 | 10 | *ERC1* | 5 | 7 |
| *RPS6KB2* | 1 | 1 | *TBX22* | 7 | 10 | *MAML2* | 5 | 7 |
| *SDHB* | 1 | 1 | *FGFR4* | 7 | 10 | *TERT* | 5 | 7 |
| *SETD2* | 1 | 1 | *BRD3* | 7 | 10 | *EPHB6* | 5 | 7 |
| *SLC29A1* | 1 | 1 | *KDR* | 7 | 10 | *HOOK3* | 5 | 7 |
| *SMAD3* | 1 | 1 | *COL1A1* | 7 | 10 | *POU5F1* | 5 | 7 |
| *SMAD4* | 1 | 1 | *HNF1A* | 7 | 10 | *FUBP1* | 5 | 7 |
| *SRC* | 1 | 1 | *TLN1* | 7 | 10 | *MAP2K7* | 5 | 7 |
| *STK11* | 1 | 1 | *ERCC3* | 7 | 10 | *AKAP9* | 5 | 7 |
| *SUZ12* | 1 | 1 | *PRKCB* | 7 | 10 | *ASPSCR1* | 5 | 7 |
| *TAF1* | 1 | 1 | *MMP2* | 7 | 10 | *PAX8* | 5 | 7 |
| *TEC* | 1 | 1 | *SRC* | 7 | 10 | *GLI3* | 5 | 7 |
| *TERT* | 1 | 1 | *MAPK11* | 7 | 10 | *GRB10* | 5 | 7 |
| *TFE3* | 1 | 1 | *ARID1A* | 7 | 10 | *GRIN2D* | 5 | 7 |
| *TFEB* | 1 | 1 | *EWSR1* | 7 | 10 | *TLN1* | 5 | 7 |
| *TLN1* | 1 | 1 | *FGFR2* | 7 | 10 | *ERCC3* | 5 | 7 |
| *TPR* | 1 | 1 | *ERG* | 7 | 10 | *PRKCB* | 5 | 7 |
| *TRIM27* | 1 | 1 | *CIC* | 7 | 10 | *MMP2* | 5 | 7 |
| *TSC2* | 1 | 1 | *KEAP1* | 7 | 10 | *SRC* | 5 | 7 |
| *WRN* | 1 | 1 | *PPARG* | 7 | 10 | *MAPK11* | 5 | 7 |
| *XPC* | 1 | 1 | *XPC* | 7 | 10 | *MED12* | 5 | 7 |
|  |  |  | *MEN1* | 7 | 10 | *MAP3K14* | 5 | 7 |
|  |  |  | *PPP2R1A* | 7 | 10 | *ETV4* | 5 | 7 |
|  |  |  | *TFG* | 7 | 10 | *CCNE1* | 5 | 7 |
|  |  |  | *KLF6* | 7 | 10 | *NTRK2* | 5 | 7 |
|  |  |  | *PATZ1* | 7 | 10 | *PHOX2B* | 5 | 7 |
|  |  |  | *NF1* | 6 | 9 | *AMFR* | 5 | 7 |
|  |  |  | *TAF1* | 6 | 9 | *TFE3* | 5 | 7 |
|  |  |  | *KIT* | 6 | 9 | *GNA11* | 5 | 7 |
|  |  |  | *CHUK* | 6 | 9 | *BLM* | 5 | 7 |
|  |  |  | *SOS1* | 6 | 9 | *BMPR1A* | 5 | 7 |
|  |  |  | *CDH1* | 6 | 9 | *MAP3K2* | 5 | 7 |
|  |  |  | *PDGFRA* | 6 | 9 | *SFPQ* | 5 | 7 |
|  |  |  | *EBF1* | 6 | 9 | *GSK3B* | 5 | 7 |
|  |  |  | *MITF* | 6 | 9 | *SSX6* | 5 | 7 |
|  |  |  | *TRIM33* | 6 | 9 | *CDK8* | 5 | 7 |
|  |  |  | *BAX* | 6 | 9 | *PTEN* | 5 | 7 |
|  |  |  | *BIRC7* | 6 | 9 | *MDM2* | 5 | 7 |
|  |  |  | *MED12* | 6 | 9 | *CRKL* | 5 | 7 |
|  |  |  | *MAP3K14* | 6 | 9 | *GRM1* | 5 | 7 |
|  |  |  | *ETV4* | 6 | 9 | *TEC* | 5 | 7 |
|  |  |  | *CCNE1* | 6 | 9 | *RAD51B* | 5 | 7 |
|  |  |  | *AKT1* | 6 | 9 | *RECQL4* | 4 | 6 |
|  |  |  | *ACSL3* | 6 | 9 | *IKBKE* | 4 | 6 |
|  |  |  | *SS18L1* | 6 | 9 | *EXT1* | 4 | 6 |
|  |  |  | *BRAF* | 6 | 9 | *NTRK3* | 4 | 6 |
|  |  |  | *WT1* | 6 | 9 | *CRTC3* | 4 | 6 |
|  |  |  | *MUTYH* | 6 | 9 | *CRTC1* | 4 | 6 |
|  |  |  | *MN1* | 6 | 9 | *EPHA3* | 4 | 6 |
|  |  |  | *IRS4* | 6 | 9 | *EEF2K* | 4 | 6 |
|  |  |  | *MOS* | 6 | 9 | *SRGAP3* | 4 | 6 |
|  |  |  | *MAPK14* | 6 | 9 | *NF2* | 4 | 6 |
|  |  |  | *LPP* | 6 | 9 | *CSNK1D* | 4 | 6 |
|  |  |  | *MAP3K12* | 6 | 9 | *BGN* | 4 | 6 |
|  |  |  | *CXCR7* | 6 | 9 | *TMPRSS2* | 4 | 6 |
|  |  |  | *DAXX* | 6 | 9 | *ARAF* | 4 | 6 |
|  |  |  | *CCND1* | 6 | 9 | *ERBB2* | 4 | 6 |
|  |  |  | *IL6ST* | 5 | 7 | *ARID1A* | 4 | 6 |
|  |  |  | *APC* | 5 | 7 | *AKT1* | 4 | 6 |
|  |  |  | *PBRM1* | 5 | 7 | *ACSL3* | 4 | 6 |
|  |  |  | *PCM1* | 5 | 7 | *SS18L1* | 4 | 6 |
|  |  |  | *PLD2* | 5 | 7 | *STK36* | 4 | 6 |
|  |  |  | *RB1* | 5 | 7 | *PIK3CG* | 4 | 6 |
|  |  |  | *KTN1* | 5 | 7 | *ROCK1* | 4 | 6 |
|  |  |  | *MAP2K2* | 5 | 7 | *CCDC6* | 4 | 6 |
|  |  |  | *EML4* | 5 | 7 | *ERBB3* | 4 | 6 |
|  |  |  | *ALDH2* | 5 | 7 | *NONO* | 4 | 6 |
|  |  |  | *FGFR3* | 5 | 7 | *HRAS* | 4 | 6 |
|  |  |  | *MLH1* | 5 | 7 | *SMAD2* | 4 | 6 |
|  |  |  | *CDKN2A* | 5 | 7 | *CYLD* | 4 | 6 |
|  |  |  | *TCF7L2* | 5 | 7 | *MAPK9* | 4 | 6 |
|  |  |  | *LIFR* | 5 | 7 | *GPC3* | 4 | 6 |
|  |  |  | *ETS1* | 5 | 7 | *SSX4* | 4 | 6 |
|  |  |  | *FLCN* | 5 | 7 | *IGF1* | 4 | 6 |
|  |  |  | *SUFU* | 5 | 7 | *MAPK8* | 4 | 6 |
|  |  |  | *PRUNE2* | 5 | 7 | *SDHD* | 4 | 6 |
|  |  |  | *BAP1* | 5 | 7 | *HMGA2* | 4 | 6 |
|  |  |  | *NTRK2* | 5 | 7 | *ATF1* | 4 | 6 |
|  |  |  | *PHOX2B* | 5 | 7 | *GOLGA5* | 4 | 6 |
|  |  |  | *AMFR* | 5 | 7 | *PAFAH1B2* | 4 | 6 |
|  |  |  | *TFE3* | 5 | 7 | *TPR* | 3 | 4 |
|  |  |  | *GNA11* | 5 | 7 | *IKBKB* | 3 | 4 |
|  |  |  | *STK36* | 5 | 7 | *GATA3* | 3 | 4 |
|  |  |  | *PIK3CG* | 5 | 7 | *RUNX1T1* | 3 | 4 |
|  |  |  | *ROCK1* | 5 | 7 | *STAT1* | 3 | 4 |
|  |  |  | *FAS* | 5 | 7 | *SMO* | 3 | 4 |
|  |  |  | *FKBP9* | 5 | 7 | *RPS6KB2* | 3 | 4 |
|  |  |  | *IDH1* | 5 | 7 | *DDB2* | 3 | 4 |
|  |  |  | *TGFBR2* | 5 | 7 | *MAPK8IP3* | 3 | 4 |
|  |  |  | *PALB2* | 5 | 7 | *ERCC4* | 3 | 4 |
|  |  |  | *FRS2* | 5 | 7 | *CDC73* | 3 | 4 |
|  |  |  | *MET* | 5 | 7 | *MYB* | 3 | 4 |
|  |  |  | *CREB3L2* | 5 | 7 | *C11orf10* | 3 | 4 |
|  |  |  | *THRAP3* | 5 | 7 | *EWSR1* | 3 | 4 |
|  |  |  | *CHN1* | 5 | 7 | *FGFR2* | 3 | 4 |
|  |  |  | *CDKN1A* | 5 | 7 | *ERG* | 3 | 4 |
|  |  |  | *HMGA1* | 5 | 7 | *CIC* | 3 | 4 |
|  |  |  | *KLK2* | 5 | 7 | *KEAP1* | 3 | 4 |
|  |  |  | *INSRR* | 5 | 7 | *BRAF* | 3 | 4 |
|  |  |  | *SH2D2A* | 5 | 7 | *WT1* | 3 | 4 |
|  |  |  | *PDGFA* | 5 | 7 | *MUTYH* | 3 | 4 |
|  |  |  | *RPS6KA3* | 4 | 6 | *MN1* | 3 | 4 |
|  |  |  | *EPHA5* | 4 | 6 | *FAS* | 3 | 4 |
|  |  |  | *ATRX* | 4 | 6 | *FKBP9* | 3 | 4 |
|  |  |  | *CSNK1A1* | 4 | 6 | *IDH1* | 3 | 4 |
|  |  |  | *HSP90AA1* | 4 | 6 | *TGFBR2* | 3 | 4 |
|  |  |  | *ROS1* | 4 | 6 | *PALB2* | 3 | 4 |
|  |  |  | *CD74* | 4 | 6 | *FRS2* | 3 | 4 |
|  |  |  | *CTNNB1* | 4 | 6 | *MAP2K1* | 3 | 4 |
|  |  |  | *BRIP1* | 4 | 6 | *SSX1* | 3 | 4 |
|  |  |  | *DPYD* | 4 | 6 | *GUCY1A2* | 3 | 4 |
|  |  |  | *BLM* | 4 | 6 | *FAM123B* | 3 | 4 |
|  |  |  | *BMPR1A* | 4 | 6 | *JAZF1* | 3 | 4 |
|  |  |  | *MAP3K2* | 4 | 6 | *CREB1* | 3 | 4 |
|  |  |  | *CCDC6* | 4 | 6 | *FHIT* | 3 | 4 |
|  |  |  | *ERBB3* | 4 | 6 | *OMD* | 3 | 4 |
|  |  |  | *MAP2K1* | 4 | 6 | *SUZ12* | 3 | 4 |
|  |  |  | *SSX1* | 4 | 6 | *C15orf55* | 3 | 4 |
|  |  |  | *GUCY1A2* | 4 | 6 | *LHFP* | 3 | 4 |
|  |  |  | *FAM123B* | 4 | 6 | *SP1* | 3 | 4 |
|  |  |  | *JAZF1* | 4 | 6 | *ACTA2* | 3 | 4 |
|  |  |  | *PRKAR1A* | 4 | 6 | *NKX2-1* | 3 | 4 |
|  |  |  | *RPS6KA4* | 4 | 6 | *VTI1A* | 3 | 4 |
|  |  |  | *MYCN* | 4 | 6 | *NDRG1* | 2 | 3 |
|  |  |  | *SOX2* | 4 | 6 | *UBR5* | 2 | 3 |
|  |  |  | *SMAD3* | 4 | 6 | *MYC* | 2 | 3 |
|  |  |  | *VHL* | 4 | 6 | *DDR2* | 2 | 3 |
|  |  |  | *TLR4* | 4 | 6 | *MCL1* | 2 | 3 |
|  |  |  | *FOS* | 4 | 6 | *PARP1* | 2 | 3 |
|  |  |  | *IGFBP3* | 4 | 6 | *FH* | 2 | 3 |
|  |  |  | *NRAS* | 4 | 6 | *NBN* | 2 | 3 |
|  |  |  | *RIPK1* | 4 | 6 | *RGL1* | 2 | 3 |
|  |  |  | *MYCL1* | 4 | 6 | *PBX1* | 2 | 3 |
|  |  |  | *TCEA1* | 4 | 6 | *TFEB* | 2 | 3 |
|  |  |  | *SS18* | 4 | 6 | *AKT2* | 2 | 3 |
|  |  |  | *GOPC* | 4 | 6 | *AXIN2* | 2 | 3 |
|  |  |  | *MKRN3* | 4 | 6 | *SLC45A3* | 2 | 3 |
|  |  |  | *FEV* | 4 | 6 | *PRKCA* | 2 | 3 |
|  |  |  | *TRIM62* | 4 | 6 | *CREB3L1* | 2 | 3 |
|  |  |  | *EXT2* | 4 | 6 | *PAX3* | 2 | 3 |
|  |  |  | *CHCHD7* | 4 | 6 | *RAF1* | 2 | 3 |
|  |  |  | *ELK4* | 4 | 6 | *THOC5* | 2 | 3 |
|  |  |  | *BUB1B* | 3 | 4 | *PIK3CA* | 2 | 3 |
|  |  |  | *GNAQ* | 3 | 4 | *TRIM27* | 2 | 3 |
|  |  |  | *TP53* | 3 | 4 | *MAPK13* | 2 | 3 |
|  |  |  | *KDM6A* | 3 | 4 | *ETV1* | 2 | 3 |
|  |  |  | *TCF12* | 3 | 4 | *IDH2* | 2 | 3 |
|  |  |  | *PRKD1* | 3 | 4 | *PPARG* | 2 | 3 |
|  |  |  | *SDHB* | 3 | 4 | *XPC* | 2 | 3 |
|  |  |  | *FBXW7* | 3 | 4 | *IRS4* | 2 | 3 |
|  |  |  | *PMS2* | 3 | 4 | *MOS* | 2 | 3 |
|  |  |  | *BIVM-ERCC5* | 3 | 4 | *MET* | 2 | 3 |
|  |  |  | *SFPQ* | 3 | 4 | *CREB3L2* | 2 | 3 |
|  |  |  | *GSK3B* | 3 | 4 | *PRKAR1A* | 2 | 3 |
|  |  |  | *SSX6* | 3 | 4 | *RPS6KA4* | 2 | 3 |
|  |  |  | *CDK8* | 3 | 4 | *MYCN* | 2 | 3 |
|  |  |  | *PTEN* | 3 | 4 | *SOX2* | 2 | 3 |
|  |  |  | *NONO* | 3 | 4 | *SMAD3* | 2 | 3 |
|  |  |  | *HRAS* | 3 | 4 | *VHL* | 2 | 3 |
|  |  |  | *SMAD2* | 3 | 4 | *TLR4* | 2 | 3 |
|  |  |  | *CREB1* | 3 | 4 | *FOS* | 2 | 3 |
|  |  |  | *FHIT* | 3 | 4 | *XPA* | 2 | 3 |
|  |  |  | *OMD* | 3 | 4 | *FUS* | 2 | 3 |
|  |  |  | *SUZ12* | 3 | 4 | *ZNF331* | 2 | 3 |
|  |  |  | *XPA* | 3 | 4 | *JUN* | 2 | 3 |
|  |  |  | *FUS* | 3 | 4 | *CDKN2C* | 2 | 3 |
|  |  |  | *ZNF331* | 3 | 4 | *HNRNPA2B1* | 2 | 3 |
|  |  |  | *JUN* | 3 | 4 | *ERCC5* | 2 | 3 |
|  |  |  | *CDKN2C* | 3 | 4 | *TGFBR1* | 2 | 3 |
|  |  |  | *CDH11* | 3 | 4 | *NCOA4* | 2 | 3 |
|  |  |  | *ZNF668* | 3 | 4 | *LOC100509575* | 2 | 3 |
|  |  |  | *AURKA* | 3 | 4 | *PTK2* | 1 | 1 |
|  |  |  | *TGFB3* | 3 | 4 | *NTRK1* | 1 | 1 |
|  |  |  | *CNBP* | 3 | 4 | *TPM3* | 1 | 1 |
|  |  |  | *MAPK3* | 3 | 4 | *PRCC* | 1 | 1 |
|  |  |  | *SETD2* | 2 | 3 | *ETV6* | 1 | 1 |
|  |  |  | *PIK3R1* | 2 | 3 | *NCOA1* | 1 | 1 |
|  |  |  | *CDK12* | 2 | 3 | *AKT3* | 1 | 1 |
|  |  |  | *MAP3K1* | 2 | 3 | *EPHB1* | 1 | 1 |
|  |  |  | *BRCA1* | 2 | 3 | *CANT1* | 1 | 1 |
|  |  |  | *CTNNA1* | 2 | 3 | *MEN1* | 1 | 1 |
|  |  |  | *SMAD4* | 2 | 3 | *PPP2R1A* | 1 | 1 |
|  |  |  | *CHEK1* | 2 | 3 | *TFG* | 1 | 1 |
|  |  |  | *GAB1* | 2 | 3 | *KLF6* | 1 | 1 |
|  |  |  | *ARID2* | 2 | 3 | *PATZ1* | 1 | 1 |
|  |  |  | *NR4A3* | 2 | 3 | *MAPK14* | 1 | 1 |
|  |  |  | *WIF1* | 2 | 3 | *LPP* | 1 | 1 |
|  |  |  | *TSC1* | 2 | 3 | *MAP3K12* | 1 | 1 |
|  |  |  | *MRE11A* | 2 | 3 | *THRAP3* | 1 | 1 |
|  |  |  | *FLI1* | 2 | 3 | *CHN1* | 1 | 1 |
|  |  |  | *MDM2* | 2 | 3 | *CDKN1A* | 1 | 1 |
|  |  |  | *CRKL* | 2 | 3 | *HMGA1* | 1 | 1 |
|  |  |  | *GRM1* | 2 | 3 | *IGFBP3* | 1 | 1 |
|  |  |  | *CYLD* | 2 | 3 | *NRAS* | 1 | 1 |
|  |  |  | *MAPK9* | 2 | 3 | *RIPK1* | 1 | 1 |
|  |  |  | *GPC3* | 2 | 3 | *MYCL1* | 1 | 1 |
|  |  |  | *SSX4* | 2 | 3 | *TCEA1* | 1 | 1 |
|  |  |  | *IGF1* | 2 | 3 | *SS18* | 1 | 1 |
|  |  |  | *C15orf55* | 2 | 3 | *GOPC* | 1 | 1 |
|  |  |  | *LHFP* | 2 | 3 | *MKRN3* | 1 | 1 |
|  |  |  | *SP1* | 2 | 3 | *FEV* | 1 | 1 |
|  |  |  | *ACTA2* | 2 | 3 | *TRIM62* | 1 | 1 |
|  |  |  | *HNRNPA2B1* | 2 | 3 | *CDH11* | 1 | 1 |
|  |  |  | *ERCC5* | 2 | 3 | *ZNF668* | 1 | 1 |
|  |  |  | *HERPUD1* | 2 | 3 | *HERPUD1* | 1 | 1 |
|  |  |  | *KRAS* | 2 | 3 | *KRAS* | 1 | 1 |
|  |  |  | *CDK4* | 2 | 3 | *PDPK1* | 1 | 1 |
|  |  |  | *SDCCAG8* | 2 | 3 | *DDX5* | 1 | 1 |
|  |  |  | *TSHR* | 1 | 1 | *HIST1H1B* | 1 | 1 |
|  |  |  | *CDC25C* | 1 | 1 | *GNAS-AS1* | 1 | 1 |
|  |  |  | *PLK2* | 1 | 1 |  |  |  |
|  |  |  | *DICER1* | 1 | 1 |  |  |  |
|  |  |  | *TAF15* | 1 | 1 |  |  |  |
|  |  |  | *TEC* | 1 | 1 |  |  |  |
|  |  |  | *RAD51B* | 1 | 1 |  |  |  |
|  |  |  | *MAPK8* | 1 | 1 |  |  |  |
|  |  |  | *SDHD* | 1 | 1 |  |  |  |
|  |  |  | *HMGA2* | 1 | 1 |  |  |  |
|  |  |  | *ATF1* | 1 | 1 |  |  |  |
|  |  |  | *NKX2-1* | 1 | 1 |  |  |  |
|  |  |  | *TGFBR1* | 1 | 1 |  |  |  |
|  |  |  | *PDPK1* | 1 | 1 |  |  |  |
|  |  |  | *DDX5* | 1 | 1 |  |  |  |
|  |  |  | *HIST1H1B* | 1 | 1 |  |  |  |
|  |  |  | *PLAG1* | 1 | 1 |  |  |  |
|  |  |  | *NFE2L2* | 1 | 1 |  |  |  |
|  |  |  | *DDIT3* | 1 | 1 |  |  |  |
|  |  |  | *FOXL2* | 1 | 1 |  |  |  |
|  |  |  | *MCM4* | 1 | 1 |  |  |  |

The list shows 157 genes harboring somatic mutations, 365 amplified genes, and 346 deleted genes, including their observed frequencies, in Korean patients with triple-negative breast cancer (TNBC).

**Supplementary Table 8: Comparisons of frequently altered genes in this cohort of Korean patients with triple-negative breast cancer (TNBC) and Western European-North American (WENA) patients with TNBC**

| **70 Korean TNBCs in this study** | | **65 WENA TNBCs in TCGA database** | |
| --- | --- | --- | --- |
| **Gene** | **Frequency (%)** | **Gene** | **Frequency (%)** |
| **Highly Frequently Mutated Genes** | | | |
| *TP53* | 45 (64%) | *TP53* | 35 (54%) |
| *NOTCH4* | 19 | *TNS3* | 14 |
| *NOTCH3* | 14 | *PIK3CA* | 7 (11%) |
| *GNAS* | 12 | *ARHGAP5* | 7 |
| *BRD4* | 10 | *USH2A* | 7 |
| *MN1* | 10 | *MYO3A* | 6 |
| *MLL2* | 9 | *PTEN* | 6 |
| *PAX8* | 9 | *PCDHA10* | 5 |
| *EXT1* | 8 | *PPFIBP2* | 5 |
| *PIK3CA* | 8 (11%) | *ATR* | 5 |
| *ETV4* | 7 | *RB1* | 4 |
| *GLI3* | 7 | *ZNF142* | 4 |
| *HOOK3* | 7 | *UBAP2L* | 4 |
| *MYCL1* | 7 | *GPR112* | 4 |
| *SRGAP3* | 7 | *HECW1* | 4 |
| *ARID2* | 6 | *UBR5* | 4 |
| *COL1A1* | 6 | *LRP2* | 4 |
| *MTOR* | 6 | *MDN1* | 4 |
| *TRIM62* | 6 | *COL6A3* | 4 |
| *ATM* | 5 | *SYNE1* | 4 |
| *BAP1* | 5 | *TTN* | 4 |
| **Highly Frequently Amplified Genes** | | | |
| *NDRG1* | 36 | *EGFR* | 5 |
| *UBR5* | 32 | *SEC61G* | 4 |
| *PTK2* | 32 | *NOTCH2* | 3 |
| *RECQL4* | 26 | *HMGCS2* | 2 |
| *MYC* | 26 | *PSPH* | 2 |
| *IKBKE* | 25 | *RGMA* | 2 |
| *EXT1* | 25 | *CCT6A* | 2 |
| *CDK2* | 24 | *PHKG1* | 2 |
| *NTRK1* | 24 | *HUWE1* | 1 |
| *DDR2* | 22 | *HEXA* | 1 |
| *MCL1* | 22 | *ADCY9* | 1 |
| *TPR* | 20 | *LRP6* | 1 |
| *PARP1* | 19 | *PORCN* | 1 |
| *TPM3* | 19 | *IQSEC2* | 1 |
| *PRCC* | 19 | *GRIN2A* | 1 |
| *RNF213* | 19 | *MYO9A* | 1 |
| *ERC1* | 19 | *ARHGDIB* | 1 |
| *FH* | 18 | *MYH11* | 1 |
| *NBN* | 18 | *GUCY2C* | 1 |
| *RGL1* | 17 | *SLC2A1* | 1 |
| *PTPRD* | 16 | *AKAP8* | 1 |
| *TIAM1* | 16 | *GAB2* | 1 |
| *NOTCH4* | 16 | *MAGOHB* | 1 |
| *IGF1R* | 16 | *SEC23B* | 1 |
| *IKBKB* | 16 | *PEX5* | 1 |
| *GATA3* | 16 | *HDAC6* | 1 |
| *PBX1* | 16 | *SUV39H1* | 1 |
| *MLL2* | 15 | *OTUD5* | 1 |
| *FLT4* | 15 | *ADRB3* | 1 |
| *EGFR* | 15 | *RBMX* | 1 |
| *RPTOR* | 15 | *EIF4EBP1* | 1 |
| **Highly Frequently Homozygously Deleted Genes** | | | |
| *WRN* | 30 | *PARK2* | 6 |
| *IL6ST* | 22 | *RB1* | 5 (8%) |
| *APC* | 21 | *OR4N4* | 5 |
| *PTK2B* | 20 | *PTEN* | 3 |
| *NF1* | 19 | *TLR7* | 3 |
| *SETD2* | 18 | *PRPS2* | 3 |
| *PTPRD* | 17 | *PAPSS2* | 3 |
| *PBRM1* | 17 | *MAP3K1* | 3 (5%) |
| MLL3 | 16 | *ARHGAP6* | 3 |
| *PCM1* | 16 | *OR4K2* | 3 |
| *PLD2* | 15 | *ATXN3L* | 3 |
| *PIK3R1* | 15 | *OFD1* | 3 |
| *CDK2* | 14 | *TLR3* | 3 |
| *CSF1R* | 14 | *TMSB4X* | 3 |
| *BUB1B* | 14 | *PORCN* | 2 |
| *CDK12* | 14 | *HDAC6* | 2 |
| *MTOR* | 13 | *SUV39H1* | 2 |
| *CHEK2* | 13 | *OTUD5* | 2 |
| *ATM* | 13 | *HIST1H4A* | 2 |
| *RB1* | 13 (19%) | *PRKG1* | 2 |
| *MAP3K1* | 13 (19%) | *FAF1* | 2 |
| *TIAM1* | 12 | *ROBO2* | 2 |
| *ERCC2* | 12 | *LIPC* | 2 |
| *KTN1* | 12 | *ITGA2* | 2 |
| *BRCA1* | 12 | *MBTPS1* | 2 |
| *TSHR* | 12 | *PRKX* | 2 |
| *MLL2* | 11 | *HRH1* | 2 |
| *PRKDC* | 11 | *NDUFS4* | 2 |
| *TCF4* | 11 | *ACSL1* | 2 |
| *USP6* | 11 | *CASP3* | 2 |
| *RPS6KA2* | 11 | *PIGA* | 2 |

Few mutations were found in both patient sets, except for somatic mutations in *TP53* and *PIK3CA*, and homozygous deletions of *RB1* and *MAP3K1*.

**Supplementary Table 9: Full list of the 368 target genes analyzed in this study. HaloPlex probes were designed to capture whole exon regions of these genes**

| No. | Gene | Coverage | Source |
| --- | --- | --- | --- |
| 1 | *ACSL3* | 91.50% | CCDS2455.1 |
| 2 | *AKAP12* | 96.10% | CCDS5229.1, CCDS5230.1 |
| 3 | *AKAP9* | 98.10% | CCDS5622.1 |
| 4 | *AKT1* | 98.30% | CCDS9994.1 |
| 5 | *AKT2* | 97.90% | CCDS12552.1 |
| 6 | *AKT3* | 99.70% | CCDS31076.1, CCDS31077.1 |
| 7 | *ALDH2* | 91.40% | CCDS9155.1 |
| 8 | *ALK* | 99.40% | CCDS33172.1 |
| 9 | *AMFR* | 98.80% | CCDS10758.1 |
| 10 | *APC* | 98.60% | CCDS4107.1 |
| 11 | *ARAF* | 98.80% | CCDS35232.1 |
| 12 | *ARID1A* | 92.70% | CCDS285.1, CCDS44091.1 |
| 13 | *ARID2* | 96.60% | CCDS31783.1 |
| 14 | *ASPSCR1* | 89.20% | CCDS11796.1 |
| 15 | *ATF1* | 98.50% | CCDS8803.1 |
| 16 | *ATM* | 98.00% | CCDS31669.1, CCDS31670.1 |
| 17 | *ATR* | 96.20% | CCDS3124.1 |
| 18 | *ATRX* | 96.40% | CCDS14435.1, CCDS14434.1 |
| 19 | *AURKA* | 97.40% | CCDS13451.1 |
| 20 | *AXIN2* | 96.00% | CCDS11662.1 |
| 21 | *BAP1* | 97.70% | CCDS2853.1 |
| 22 | *BAX* | 95.40% | CCDS12743.1, CCDS12744.1, CCDS12742.1 |
| 23 | *BGN* | 90.80% | CCDS14721.1 |
| 24 | *BIRC7* | 100.00% | CCDS13513.1, CCDS13512.1 |
| 25 | *BLM* | 97.70% | CCDS10363.1 |
| 26 | *BMPR1A* | 99.70% | CCDS7378.1 |
| 27 | *BRAF* | 88.70% | CCDS5863.1 |
| 28 | *BRCA1* | 92.40% | CCDS11453.1 |
| 29 | *BRCA2* | 97.10% | CCDS9344.1 |
| 30 | *BRD3* | 93.90% | CCDS6980.1 |
| 31 | *BRD4* | 99.20% | CCDS12328.1, CCDS46004.1 |
| 32 | *BRIP1* | 96.50% | CCDS11631.1 |
| 33 | *BUB1B* | 99.90% | CCDS10053.1 |
| 34 | *C11orf10* | 99.20% | CCDS8009.1 |
| 35 | *C15orf55* | 98.90% | CCDS32190.1 |
| 36 | *CANT1* | 95.20% | CCDS11760.1 |
| 37 | *CCDC6* | 98.90% | CCDS7257.1 |
| 38 | *CCNB1IP1* | 98.20% | CCDS9547.1 |
| 39 | *CCND1* | 89.50% | CCDS8191.1 |
| 40 | *CCNE1* | 98.00% | CCDS12419.1, CCDS46035.1 |
| 41 | *CD74* | 97.40% | CCDS47309.1, CCDS47308.1, CCDS34276.1 |
| 42 | *CDC25C* | 99.30% | CCDS4203.1, CCDS4202.1 |
| 43 | *CDC73* | 97.30% | CCDS1382.1 |
| 44 | *CDH1* | 97.20% | CCDS10869.1 |
| 45 | *CDH11* | 99.20% | CCDS10803.1 |
| 46 | *CDK12* | 99.10% | NM_016507, NM_015083 |
| 47 | *CDK2* | 95.60% | CCDS8899.1, CCDS8898.1 |
| 48 | *CDK4* | 98.90% | CCDS8953.1 |
| 49 | *CDK8* | 98.70% | CCDS9317.1 |
| 50 | *CDKN1A* | 90.10% | CCDS4824.1 |
| 51 | *CDKN1B* | 98.30% | CCDS8653.1 |
| 52 | *CDKN2A* | 91.80% | CCDS6510.1, CCDS6511.1, CCDS34998.1 |
| 53 | *CDKN2C* | 98.00% | CCDS555.1 |
| 54 | *CHCHD7* | 100.00% | CCDS34895.1, CCDS6166.2, CCDS34896.1 |
| 55 | *CHEK1* | 99.40% | CCDS8459.1 |
| 56 | *CHEK2* | 90.00% | CCDS33629.1, CCDS13844.1, CCDS13843.1 |
| 57 | *CHN1* | 98.90% | CCDS46454.1, CCDS46455.1 |
| 58 | *CHUK* | 98.50% | CCDS7488.1 |
| 59 | *CIC* | 98.80% | CCDS12601.1 |
| 60 | *CNBP* | 100.00% | CCDS46906.1, CCDS46908.1, CCDS46907.1, CCDS3056.1 |
| 61 | *COL1A1* | 90.50% | CCDS11561.1 |
| 62 | *COX6C* | 100.00% | CCDS6284.1 |
| 63 | *CREB1* | 99.00% | CCDS2375.1, CCDS2374.1 |
| 64 | *CREB3L1* | 97.90% | NM_052854 |
| 65 | *CREB3L2* | 99.00% | CCDS34760.1 |
| 66 | *CRKL* | 99.90% | CCDS13785.1 |
| 67 | *CRTC1* | 97.30% | CCDS42525.1, CCDS32963.1 |
| 68 | *CRTC3* | 99.50% | CCDS32331.1, CCDS45348.1 |
| 69 | *CSF1R* | 96.00% | CCDS4302.1 |
| 70 | *CSNK1A1* | 95.80% | CCDS47304.1, CCDS47303.1 |
| 71 | *CSNK1D* | 93.80% | CCDS11805.1, CCDS11806.1 |
| 72 | *CTNNA1* | 98.30% | CCDS34243.1 |
| 73 | *CTNNB1* | 98.60% | CCDS2694.1 |
| 74 | *CUX1* | 98.40% | CCDS47672.1, CCDS5720.1, CCDS5721.1 |
| 75 | *CXCR7* | 96.60% | CCDS2516.1 |
| 76 | *CYLD* | 98.10% | CCDS42164.1, CCDS45482.1 |
| 77 | *DAXX* | 98.20% | CCDS4776.1 |
| 78 | *DDB2* | 98.60% | CCDS7927.1 |
| 79 | *DDIT3* | 98.00% | CCDS8943.1 |
| 80 | *DDR2* | 97.20% | CCDS1241.1 |
| 81 | *DDX5* | 99.50% | CCDS11659.1 |
| 82 | *DICER1* | 98.80% | CCDS9931.1 |
| 83 | *DPYD* | 97.40% | CCDS30777.1 |
| 84 | *DUX4* | 13.00% | NM_033178 |
| 85 | *EBF1* | 89.90% | CCDS4343.1 |
| 86 | *EEF2K* | 94.90% | CCDS10604.1 |
| 87 | *EGFR* | 92.60% | CCDS5515.1, CCDS5514.1, CCDS5516.1, CCDS47587.1 |
| 88 | *ELK4* | 91.80% | CCDS1456.1, CCDS1457.1 |
| 89 | *EML4* | 99.50% | CCDS1807.1, CCDS46266.1 |
| 90 | *EP300* | 98.70% | CCDS14010.1 |
| 91 | *EPHA3* | 99.40% | CCDS46875.1, CCDS2922.1 |
| 92 | *EPHA5* | 98.60% | CCDS3514.1, CCDS3513.1 |
| 93 | *EPHB1* | 96.80% | CCDS46921.1 |
| 94 | *EPHB6* | 95.80% | CCDS5873.2 |
| 95 | *ERBB2* | 97.80% | CCDS32642.1, CCDS45667.1 |
| 96 | *ERBB3* | 99.20% | CCDS44918.1, CCDS31833.1 |
| 97 | *ERBB4* | 98.30% | CCDS42811.1, CCDS2394.1 |
| 98 | *ERC1* | 99.80% | CCDS8508.1 |
| 99 | *ERCC2* | 96.70% | CCDS33049.1, CCDS46112.1 |
| 100 | *ERCC3* | 99.10% | CCDS2144.1 |
| 101 | *ERCC4* | 97.20% | CCDS32390.1 |
| 102 | *ERCC5* | 96.00% | CCDS32004.1 |
| 103 | *ERG* | 98.20% | CCDS13658.1, CCDS46648.1, CCDS46649.1, CCDS13657.1 |
| 104 | *ETS1* | 97.00% | CCDS44767.1, CCDS8475.1 |
| 105 | *ETV1* | 86.20% | NM_001163150, NM_001163149, NM_004956, NM_001163147, NM_001163148, NM_001163152, NM_001163151 |
| 106 | *ETV4* | 96.00% | CCDS11465.1 |
| 107 | *ETV5* | 98.50% | CCDS33906.1 |
| 108 | *ETV6* | 99.00% | CCDS8643.1 |
| 109 | *EWSR1* | 88.80% | CCDS13851.1 |
| 110 | *EXT1* | 96.60% | CCDS6324.1 |
| 111 | *EXT2* | 97.70% | CCDS7908.1 |
| 112 | *FAM123B* | 98.20% | CCDS14377.2 |
| 113 | *FAS* | 96.60% | CCDS7398.1, CCDS7394.1, CCDS7393.1, CCDS7395.1 |
| 114 | *FBXW7* | 96.60% | CCDS3778.1, CCDS34078.1, CCDS3777.1 |
| 115 | *FEV* | 85.90% | CCDS2428.1 |
| 116 | *FGFR2* | 93.00% | CCDS7620.2, CCDS44485.1, CCDS31298.1, CCDS44486.1, CCDS44487.1, CCDS44489.1, CCDS44488.1 |
| 117 | *FGFR3* | 95.50% | CCDS3353.1, CCDS3354.1 |
| 118 | *FGFR4* | 97.40% | CCDS4411.1, CCDS4410.1 |
| 119 | *FH* | 99.50% | CCDS1617.1 |
| 120 | *FHIT* | 100.00% | CCDS2894.1 |
| 121 | *FKBP9* | 75.70% | CCDS5439.1 |
| 122 | *FLCN* | 98.70% | CCDS32579.1, CCDS32580.1 |
| 123 | *FLI1* | 96.00% | CCDS44768.1 |
| 124 | *FLNA* | 95.90% | CCDS48194.1, CCDS44021.1 |
| 125 | *FLT1* | 98.40% | CCDS9330.1 |
| 126 | *FLT4* | 91.10% | CCDS4457.1, CCDS43412.1 |
| 127 | *FOS* | 100.00% | CCDS9841.1 |
| 128 | *FOXL2* | 99.80% | CCDS3105.1 |
| 129 | *FOXO1* | 98.70% | CCDS9371.1 |
| 130 | *FRS2* | 100.00% | CCDS41809.1 |
| 131 | *FUBP1* | 99.40% | CCDS683.1 |
| 132 | *FUS* | 81.80% | CCDS10707.1 |
| 133 | *GAB1* | 87.30% | CCDS3760.1, CCDS3759.1 |
| 134 | *GATA3* | 99.00% | CCDS7083.1, CCDS31143.1 |
| 135 | *GLI1* | 95.80% | CCDS8940.1 |
| 136 | *GLI3* | 96.30% | CCDS5465.1 |
| 137 | *GNA11* | 99.90% | CCDS12103.1 |
| 138 | *GNAQ* | 87.70% | CCDS6658.1 |
| 139 | *GNAS* | 92.90% | CCDS13471.1, CCDS46622.1, CCDS42892.1, CCDS13472.1, CCDS46624.1, CCDS46623.1 |
| 140 | *GOLGA5* | 99.50% | CCDS9905.1 |
| 141 | *GOPC* | 97.20% | CCDS34523.1, CCDS5117.1 |
| 142 | *GPC3* | 99.40% | CCDS14638.1 |
| 143 | *GRB10* | 96.60% | CCDS47586.1, CCDS43583.1, CCDS43582.1 |
| 144 | *GRIN2D* | 92.80% | CCDS12719.1 |
| 145 | *GRM1* | 95.00% | CCDS5209.1, CCDS47497.1 |
| 146 | *GSK3B* | 96.00% | CCDS2996.1 |
| 147 | *GUCY1A2* | 98.50% | CCDS8335.1 |
| 148 | *HDAC4* | 92.70% | CCDS2529.1 |
| 149 | *HERPUD1* | 99.20% | CCDS32457.1, CCDS45492.1, CCDS10771.1 |
| 150 | *HIST1H1B* | 100.00% | CCDS4635.1 |
| 151 | *HMGA1* | 100.00% | CCDS4788.1, CCDS4789.1 |
| 152 | *HMGA2* | 100.00% | CCDS44936.1, CCDS31854.1 |
| 153 | *HNF1A* | 98.20% | CCDS9209.1 |
| 154 | *HNRNPA2B1* | 100.00% | CCDS5397.1, CCDS43557.1 |
| 155 | *HOOK3* | 97.80% | CCDS6139.1 |
| 156 | *HRAS* | 98.70% | CCDS7698.1, CCDS7699.1 |
| 157 | *HSP90AA1* | 95.40% | CCDS9967.1, CCDS32160.1 |
| 158 | *IDH1* | 98.00% | CCDS2381.1 |
| 159 | *IDH2* | 100.00% | CCDS10359.1 |
| 160 | *IGF1* | 100.00% | CCDS44960.1, CCDS9091.1, CCDS44961.1, CCDS44962.1 |
| 161 | *IGF1R* | 94.20% | CCDS10378.1 |
| 162 | *IGFBP3* | 94.70% | CCDS5505.1, CCDS34632.1 |
| 163 | *IKBKB* | 98.50% | CCDS6128.1 |
| 164 | *IKBKE* | 98.10% | CCDS30996.1 |
| 165 | *IL6ST* | 98.10% | CCDS3971.1, CCDS47209.1 |
| 166 | *IRS4* | 97.20% | CCDS14544.1 |
| 167 | *JAZF1* | 96.40% | CCDS5416.1 |
| 168 | *JUN* | 98.00% | CCDS610.1 |
| 169 | *KDM5C* | 98.00% | CCDS14351.1 |
| 170 | *KDM6A* | 98.30% | CCDS14265.1 |
| 171 | *KDR* | 95.00% | CCDS3497.1 |
| 172 | *KEAP1* | 95.40% | CCDS12239.1 |
| 173 | *KIAA1549* | 96.00% | CCDS47723.1 |
| 174 | *KIT* | 97.40% | CCDS3496.1, CCDS47058.1 |
| 175 | *KLF6* | 97.60% | CCDS7060.1 |
| 176 | *KLK2* | 98.10% | CCDS12808.1, CCDS42597.1 |
| 177 | *KRAS* | 100.00% | CCDS8702.1, CCDS8703.1 |
| 178 | *KTN1* | 97.60% | CCDS41957.1, CCDS41959.1, CCDS9725.1, CCDS41958.1 |
| 179 | *LHFP* | 100.00% | CCDS9369.1 |
| 180 | *LIFR* | 96.50% | CCDS3927.1 |
| 181 | *LPP* | 96.80% | CCDS3291.1 |
| 182 | *MAML2* | 94.00% | CCDS44714.1 |
| 183 | *MAP2K1* | 96.90% | CCDS10216.1 |
| 184 | *MAP2K2* | 97.60% | CCDS12120.1 |
| 185 | *MAP2K4* | 100.00% | CCDS11162.1 |
| 186 | *MAP2K7* | 98.50% | CCDS42491.1 |
| 187 | *MAP3K1* | 97.90% | CCDS43318.1 |
| 188 | *MAP3K12* | 98.80% | CCDS8860.1 |
| 189 | *MAP3K14* | 97.70% | NM_003954 |
| 190 | *MAP3K2* | 100.00% | CCDS46404.1 |
| 191 | *MAP3K6* | 92.80% | CCDS299.1 |
| 192 | *MAP4K1* | 98.00% | CCDS42564.1 |
| 193 | *MAPK11* | 94.50% | CCDS14090.1 |
| 194 | *MAPK13* | 99.20% | CCDS4818.1 |
| 195 | *MAPK14* | 94.00% | CCDS4817.1, CCDS4815.1, CCDS4816.1 |
| 196 | *MAPK3* | 99.60% | CCDS10672.1, CCDS42148.1, CCDS42149.1 |
| 197 | *MAPK8* | 100.00% | CCDS7226.1, CCDS7225.1, CCDS7223.1, CCDS7224.1 |
| 198 | *MAPK8IP3* | 94.80% | CCDS45379.1, CCDS10442.2 |
| 199 | *MAPK9* | 98.00% | CCDS4453.1, CCDS4454.1, CCDS43409.1, CCDS43410.1, CCDS47356.1 |
| 200 | *MCL1* | 75.10% | CCDS956.1, CCDS957.1 |
| 201 | *MDM2* | 95.60% | CCDS8986.2, CCDS44939.1, CCDS44941.1 |
| 202 | *MDM4* | 98.90% | CCDS1447.1 |
| 203 | *MED12* | 96.10% | CCDS43970.1 |
| 204 | *MEN1* | 98.90% | CCDS8083.1, CCDS31600.1 |
| 205 | *MET* | 100.00% | CCDS43636.1, CCDS47689.1 |
| 206 | *MITF* | 98.80% | CCDS43106.1, CCDS46864.1, CCDS46865.1, CCDS46866.1, CCDS2913.1, CCDS43107.1 |
| 207 | *MKRN3* | 97.10% | CCDS10013.1 |
| 208 | *MLH1* | 95.90% | CCDS2663.1 |
| 209 | *MLL2* | 95.80% | CCDS44873.1 |
| 210 | *MLL3* | 93.40% | CCDS5931.1 |
| 211 | *MMP2* | 94.10% | CCDS10752.1, CCDS45487.1 |
| 212 | *MN1* | 92.20% | CCDS42998.1 |
| 213 | *MOS* | 99.30% | CCDS6164.1 |
| 214 | *MRE11A* | 94.50% | CCDS8299.1, CCDS8298.1 |
| 215 | *MSH2* | 96.00% | CCDS1834.1 |
| 216 | *MSH6* | 97.60% | CCDS1836.1 |
| 217 | *MTOR* | 97.80% | CCDS127.1 |
| 218 | *MTUS2* | 97.00% | NM_001033602, NM_015233 |
| 219 | *MUTYH* | 99.90% | CCDS41322.1, CCDS44129.1, CCDS41320.1, CCDS41321.1, CCDS520.1 |
| 220 | *MYB* | 98.40% | CCDS5174.1, CCDS47482.1, CCDS47481.1 |
| 221 | *MYC* | 96.00% | CCDS6359.2 |
| 222 | *MYCL1* | 99.00% | CCDS30682.1, CCDS44117.1 |
| 223 | *MYCN* | 86.80% | CCDS1687.1 |
| 224 | *NBN* | 97.80% | CCDS6249.1 |
| 225 | *NCOA1* | 96.00% | CCDS1712.1, CCDS1713.1, CCDS42660.1 |
| 226 | *NCOA4* | 85.30% | CCDS44394.1, CCDS44393.1, CCDS7237.1 |
| 227 | *NDRG1* | 98.00% | CCDS34945.1 |
| 228 | *NF1* | 96.50% | CCDS45645.1, CCDS42292.1, CCDS11264.1 |
| 229 | *NF2* | 97.60% | CCDS13865.1, CCDS13863.1, CCDS13861.1, CCDS13862.1, CCDS13864.1 |
| 230 | *NFE2L2* | 99.90% | CCDS42782.1, CCDS46458.1, CCDS46457.1 |
| 231 | *NFIB* | 98.30% | CCDS6474.1 |
| 232 | *NFKB1* | 96.70% | CCDS3657.1 |
| 233 | *NKX2-1* | 90.80% | CCDS9659.1, CCDS41945.1 |
| 234 | *NONO* | 98.00% | CCDS14410.1 |
| 235 | *NOTCH3* | 90.60% | CCDS12326.1 |
| 236 | *NOTCH4* | 94.60% | CCDS34420.1 |
| 237 | *NR4A3* | 98.30% | CCDS6742.1, CCDS6744.1, CCDS6743.1 |
| 238 | *NRAS* | 96.80% | CCDS877.1 |
| 239 | *NTRK1* | 96.90% | CCDS30890.1, CCDS1161.1, CCDS30891.1 |
| 240 | *NTRK2* | 98.60% | CCDS35051.1, CCDS35050.1, CCDS6671.1, CCDS35053.1, CCDS35052.1 |
| 241 | *NTRK3* | 97.00% | CCDS32322.1, CCDS10340.1, CCDS32323.1 |
| 242 | *NUTM1* | 98.90% | CCDS32190.1 |
| 243 | *OMD* | 96.30% | CCDS6696.1 |
| 244 | *PAFAH1B2* | 82.20% | CCDS8380.1 |
| 245 | *PALB2* | 98.80% | CCDS32406.1 |
| 246 | *PARP1* | 99.00% | CCDS1554.1 |
| 247 | *PATZ1* | 91.70% | CCDS13895.1, CCDS13894.1, CCDS46691.1, CCDS13896.1 |
| 248 | *PAX3* | 98.50% | CCDS2448.1, CCDS2449.1, CCDS2450.1, CCDS46522.1, CCDS42825.1, CCDS42826.1, CCDS2451.1, CCDS46523.1 |
| 249 | *PAX7* | 98.20% | CCDS44074.1, CCDS186.1, CCDS44075.1 |
| 250 | *PAX8* | 98.40% | CCDS46397.1, CCDS46398.1, CCDS42736.1, CCDS42735.1, CCDS46399.1 |
| 251 | *PBRM1* | 96.60% | CCDS2860.1, CCDS2859.1, CCDS43099.1 |
| 252 | *PBX1* | 81.00% | CCDS1246.1 |
| 253 | *PCM1* | 96.00% | CCDS47812.1 |
| 254 | *PDGFA* | 93.80% | CCDS47524.1, CCDS34578.1 |
| 255 | *PDGFRA* | 99.40% | CCDS3495.1 |
| 256 | *PDPK1* | 44.70% | CCDS10472.1, CCDS10473.1 |
| 257 | *PHOX2B* | 95.00% | CCDS3463.1 |
| 258 | *PIK3CA* | 93.40% | CCDS43171.1 |
| 259 | *PIK3CG* | 99.00% | CCDS5739.1 |
| 260 | *PIK3R1* | 99.90% | CCDS3993.1, CCDS3994.1, CCDS3995.1 |
| 261 | *PLAG1* | 98.50% | CCDS6165.1, CCDS47860.1 |
| 262 | *PLCG2* | 98.50% | CCDS42204.1 |
| 263 | *PLD2* | 95.40% | CCDS11057.1 |
| 264 | *PLK2* | 98.60% | CCDS3974.1 |
| 265 | *PMS1* | 96.90% | CCDS46474.1, CCDS46473.1, CCDS2302.1 |
| 266 | *PMS2* | 75.70% | CCDS5343.1 |
| 267 | *POU5F1* | 95.30% | CCDS34391.1, CCDS47398.1 |
| 268 | *PPARG* | 98.10% | CCDS2609.1, CCDS2610.2 |
| 269 | *PPP2R1A* | 97.70% | CCDS12849.1 |
| 270 | *PRCC* | 99.30% | CCDS1158.1, CCDS1157.1 |
| 271 | *PRKAR1A* | 100.00% | CCDS11678.1 |
| 272 | *PRKCA* | 96.70% | CCDS11664.1 |
| 273 | *PRKCB* | 95.10% | CCDS10619.1, CCDS10618.1 |
| 274 | *PRKD1* | 100.00% | CCDS9637.1 |
| 275 | *PRKDC* | 97.50% | NM_006904, NM_001081640 |
| 276 | *PRUNE2* | 97.10% | CCDS47982.1 |
| 277 | *PTCH1* | 92.50% | CCDS43851.1, CCDS6714.1, CCDS47996.1, CCDS47995.1 |
| 278 | *PTEN* | 97.90% | NM_000314 |
| 279 | *PTK2* | 92.50% | CCDS6381.1 |
| 280 | *PTK2B* | 96.20% | CCDS6058.1, CCDS6057.1 |
| 281 | *PTPRD* | 97.50% | CCDS43786.1, CCDS6472.1 |
| 282 | *RAD51L1* | 92.20% | CCDS9789.1 |
| 283 | *RAF1* | 94.00% | CCDS2612.1 |
| 284 | *RB1* | 97.00% | CCDS31973.1 |
| 285 | *RECQL4* | 91.40% | NM_004260 |
| 286 | *RELA* | 95.90% | CCDS31609.1, CCDS44651.1 |
| 287 | *RET* | 97.10% | CCDS7200.1 |
| 288 | *RGL1* | 96.80% | CCDS1359.1 |
| 289 | *RICTOR* | 97.70% | CCDS34148.1 |
| 290 | *RIPK1* | 98.80% | CCDS4482.1 |
| 291 | *RNF213* | 95.80% | CCDS32761.1 |
| 292 | *ROCK1* | 89.40% | CCDS11870.2 |
| 293 | *ROS1* | 97.10% | CCDS5116.1 |
| 294 | *RPS6KA2* | 96.70% | CCDS34570.1, CCDS5294.1 |
| 295 | *RPS6KA3* | 99.60% | CCDS14197.1 |
| 296 | *RPS6KA4* | 88.30% | CCDS8073.1 |
| 297 | *RPS6KB2* | 99.70% | CCDS41677.1 |
| 298 | *RPTOR* | 96.60% | CCDS11773.1 |
| 299 | *RUNX1T1* | 97.60% | CCDS47891.1, CCDS6256.1, CCDS6257.1 |
| 300 | *SDHB* | 100.00% | CCDS176.1 |
| 301 | *SDHC* | 88.30% | CCDS1230.1, CCDS41432.1, CCDS41431.1 |
| 302 | *SDHD* | 87.70% | CCDS31678.1 |
| 303 | *SETD2* | 96.00% | CCDS2749.2 |
| 304 | *SFPQ* | 97.00% | CCDS388.1 |
| 305 | *SLC29A1* | 96.00% | CCDS4908.1 |
| 306 | *SLC45A3* | 98.90% | CCDS1458.1 |
| 307 | *SMAD2* | 94.20% | CCDS11934.1, CCDS45863.1 |
| 308 | *SMAD3* | 100.00% | CCDS10222.1, CCDS45288.1 |
| 309 | *SMAD4* | 99.20% | CCDS11950.1 |
| 310 | *SMARCA4* | 91.60% | CCDS45971.1, CCDS45973.1, CCDS12253.1, CCDS45972.1 |
| 311 | *SMARCB1* | 99.10% | CCDS13817.1, CCDS46671.1 |
| 312 | *SMO* | 96.20% | CCDS5811.1 |
| 313 | *SOS1* | 98.50% | CCDS1802.1 |
| 314 | *SOX2* | 93.60% | CCDS3239.1 |
| 315 | *SP1* | 94.40% | CCDS8857.1, CCDS44898.1 |
| 316 | *SRC* | 95.70% | CCDS13294.1 |
| 317 | *SRGAP3* | 99.40% | CCDS2572.1, CCDS33689.1 |
| 318 | *SS18* | 96.70% | CCDS32807.1 |
| 319 | *SS18L1* | 95.40% | CCDS13491.1 |
| 320 | *SSX1* | 93.00% | CCDS14287.1, CCDS14290.1 |
| 321 | *SSX2* | 8.80% | CCDS14344.1, CCDS14345.1, CCDS48129.1 |
| 322 | *SSX4* | 39.50% | CCDS35240.1, CCDS43934.1 |
| 323 | *STAT1* | 99.50% | CCDS2309.1, CCDS42793.1 |
| 324 | *STK11* | 98.20% | CCDS45896.1 |
| 325 | *STK36* | 99.70% | CCDS2421.1 |
| 326 | *SUFU* | 87.00% | CCDS7537.1 |
| 327 | *SUZ12* | 88.70% | CCDS11270.1 |
| 328 | *TAF1* | 96.30% | CCDS14412.1, CCDS35325.1 |
| 329 | *TAF15* | 65.40% | CCDS32623.1 |
| 330 | *TBX22* | 100.00% | CCDS14445.1, CCDS43975.1 |
| 331 | *TCEA1* | 97.80% | CCDS47858.1, CCDS47857.1 |
| 332 | *TCF12* | 96.90% | CCDS10159.1, CCDS10160.1, CCDS42042.1 |
| 333 | *TCF4* | 94.70% | CCDS11960.1, CCDS42438.1 |
| 334 | *TCF7L2* | 95.20% | CCDS7576.1 |
| 335 | *TEC* | 96.70% | CCDS3481.1 |
| 336 | *TERT* | 92.50% | CCDS3861.2, CCDS47186.1 |
| 337 | *TFE3* | 98.70% | CCDS14315.3 |
| 338 | *TFEB* | 98.50% | CCDS4858.1 |
| 339 | *TFG* | 99.60% | CCDS2939.1 |
| 340 | *TGFB3* | 97.80% | CCDS9846.1 |
| 341 | *TGFBR1* | 99.50% | CCDS47998.1, CCDS6738.1 |
| 342 | *TGFBR2* | 99.40% | CCDS33727.1, CCDS2648.1 |
| 343 | *THOC5* | 93.30% | CCDS13859.1 |
| 344 | *THRAP3* | 96.60% | CCDS405.1 |
| 345 | *TIAM1* | 96.30% | CCDS13609.1 |
| 346 | *TLN1* | 96.10% | CCDS35009.1 |
| 347 | *TLR4* | 97.90% | CCDS6818.1 |
| 348 | *TMPRSS2* | 98.20% | CCDS33564.1 |
| 349 | *TP53* | 86.90% | CCDS11118.1, CCDS45605.1, CCDS45606.1 |
| 350 | *TPM3* | 99.70% | CCDS1060.1, CCDS41400.1, CCDS41401.1, CCDS41402.1, CCDS41403.1 |
| 351 | *TPR* | 98.50% | CCDS41446.1 |
| 352 | *TRIM27* | 95.30% | CCDS4654.1 |
| 353 | *TRIM33* | 95.90% | CCDS873.1, CCDS872.1 |
| 354 | *TRIM62* | 98.10% | CCDS376.1 |
| 355 | *TSC1* | 99.60% | CCDS6956.1 |
| 356 | *TSC2* | 95.80% | CCDS45384.1, CCDS10458.1 |
| 357 | *TSHR* | 96.10% | CCDS9872.1, CCDS32131.1 |
| 358 | *UBR5* | 98.20% | CCDS34933.1 |
| 359 | *USP6* | 86.00% | CCDS11069.2 |
| 360 | *VHL* | 62.60% | CCDS2598.1, CCDS2597.1 |
| 361 | *VTI1A* | 100.00% | CCDS7575.2 |
| 362 | *WIF1* | 92.60% | CCDS8971.1 |
| 363 | *WRN* | 90.90% | CCDS6082.1 |
| 364 | *WT1* | 93.70% | CCDS7877.2, CCDS7878.2, CCDS44562.1,CCDS44561.1 |
| 365 | *XPA* | 98.90% | CCDS6729.1 |
| 366 | *XPC* | 95.90% | CCDS46763.1, CCDS46764.1 |
| 367 | *ZNF331* | 90.80% | CCDS33102.1 |
| 368 | *ZNF668* | 97.90% | CCDS10701.1 |

HaloPlex probes were designed to capture whole exon regions of these genes. The coverage rate of each gene is shown.

**Supplementary Table 10: Validation regions and primer sequences**

| **Somatic Variants and CNVs for Validation** | | | | | | **Primer Sequence (5' -> 3')** |
| --- | --- | --- | --- | --- | --- | --- |
| **Gene** | **NC_#** | **Chromosomal Position** | | **Mutation** | **Sample** |  |
|  |  | **start** | **end** |  |  |  |
| SNVs (Sanger sequencing) | | | | | | |
| *TP53* | NC_000017.10 | 7578212 | 7578212 | c.637C>T | TNBC030 | GTT TCT TTG CTG CCG TCT TC |
|  |  |  |  |  |  | CTT AAC CCC TCC TCC CAG AG |
|  |  | 7578271 | 7578271 | c.578A>G | TNBC045 | GTT TCT TTG CTG CCG TCT TC |
|  |  |  |  |  |  | CTT AAC CCC TCC TCC CAG AG |
| CNVs (Real-time PCR) | | | | | | |
| *NDRG1* | NC_000008.10 | 134276714 | 134277015 | Amplification | TNBC022 | GCT TCC TCA AAA CAC AGT TGG |
|  |  |  |  |  |  | GCT GGT CAT GTG GGG TTC |
|  |  |  |  |  |  | FAM - CTT CAG CC - BHQ1 |
| *WRN* | NC_000008.10 | 30924477 | 30924571 | Homozygous deletion | TNBC030 | CCA GGT CTC TGT GCA TTT CA |
|  |  |  |  |  |  | GGT AAT ACC TGA AAA CAG GAA CTG A |
|  |  |  |  |  |  | FAM - GAA ATG ATG AAA AAG CAA CAC A -BHQ1 |
| *ATM* | NC_000011.09 | 108129654 | 108129761 |  | TNBC048 | GAA TAA TTG TTT TTA TTT CTT TGT TGC |
|  |  |  |  |  |  | TTA ACA ATC GCA GGA AAA AGC |
|  |  |  |  |  |  | FAM - TGT CTT AAT TGC AGA AGA GTC CA - BHQ1 |
|  | NC_000011.09 | 108167887 | 108168120 |  | TNBC038 | AAA CAA AAG TGT TGT CTT CAT GC |
|  |  |  |  |  |  | GAA CTT CTT TTT CAC CAG TGT GG |
|  |  |  |  |  |  | FAM - TGC AGT TAT CCA AGA TGG CA - BHQ1 |
| *BRCA1* | NC_000017.11 | 41256016 | 41256252 |  | TNBC026 TNBC031 TNBC066 | TTC TAC AGA GTG AAC CCG AAA A |
|  |  |  |  |  |  | GGC TAA GGC AGG AGG ACT G |
|  |  |  |  |  |  | FAM - ATG GAG TCT TGC TCT GTG GC - BHQ1 |
|  | NC_000017.11 | 41244444 | 41246036 |  | TNBC038 | CAG CGA TAC TTT CCC AGA GC |
|  |  |  |  |  |  | TTG CAA AAC CCT TTC TCC AC |
|  |  |  |  |  |  | FAM - TGC TGA AGA CCC CAA AGA TC - BHQ1 |
| *BRCA2* | NC_000013.11 | 32915134 | 32915248 |  | TNBC011 TNBC068 | TCC AAA GAT TCA GAA AAC TAC TTT GA |
|  |  |  |  |  |  | GAA TGT GTG GCA TGA CTT GG |
|  |  |  |  |  |  | FAM - TGG AAG ATG ATG AAC TGA CAG A - BHQ1 |
|  | NC_000013.11 | 32929258 | 32929478 |  | TNBC004 TNBC014 | CAT TGA TGG ACA TGG CTC TG |
|  |  |  |  |  |  | TGA AAG GCA AAA ATT CAT CAC A |
|  |  |  |  |  |  | FAM - CAA AAA CAA CTC CAA TCA AGC A - BHQ1 |
| *TERT* | NC_000005.9 | 1253628 | 1253725 | Reference gene |  | GGC CTG AGT GAG TGT TTG G |
|  |  |  |  |  |  | TGG ACA CTC AGC CCT TGG |
|  |  |  |  |  |  | FAM - CTT CAG CC - BHQ1 |

Mutated regions validated by Sanger sequencing or quantitative PCR, and primer sequences used for validation.

# **Supplementary Data Legends**

**Supplementary Table 1.** Associations between clinicopathological features and disease-free survival (DFS) or distant metastasis-free survival (DMFS). Advanced primary tumor stage 2 (pT2) was nominally associated with recurrence risk [hazard ratio (HR) = 3.025, *P* = 0.094] and distant metastasis (HR = 2.819, *P* = 0.207), whereas pT3 was nominally associated with risk of recurrence (HR = 6.598, *P* = 0.108).

**Supplementary Table 2.** Targeted exome-sequencing statistics. Target sequencing statistics of 140 samples (70 pairs of tumor and normal samples). The distribution of read coverage depths was similar in tumor and normal samples with average target coverage greater than 130×, which is sufficient for mutation analysis.

**Supplementary Table 3.** Number of genes with somatic variants or copy number variations (CNVs). Patients with triple-negative breast cancer (TNBC) were found to have 157 mutated genes, 365 amplified genes, and 346 deleted genes. Most of the somatic variants were novel single nucleotide variants (SNVs).

**Supplementary Table 4.** Complete list of somatic mutations identified in this study, along with their chromosomal positions, frequency, and mutation type.

**Supplementary Table 5.** *BRCA1* and *BRCA2* germline mutations. Evaluation of patients with triple-negative breast cancer (TNBC) identified two novel germline mutations in *BRCA1* and one previously reported germline mutation in *BRCA2.*

**Supplementary Table 6.** List of all genetically altered genes. The list shows 157 genes harboring somatic mutations, 365 amplified genes, and 346 deleted genes, including their observed frequencies, in Korean patients with triple-negative breast cancer (TNBC).

**Supplementary Table 7.** Result of Cox proportional hazard ratio analysis. Homozygous deletions of three genes were significantly associated with disease-free survival (DFS), and *MITF* was significantly associated with distant metastasis-free survival (DMFS).

**Supplementary Table 8.** Comparisons of frequently altered genes in this cohort of Korean patients with triple-negative breast cancer (TNBC) and Western European-North American (WENA) patients with TNBC. Few mutations were found in both patient sets, except for somatic mutations in *TP53* and *PIK3CA*, and homozygous deletions of *RB1* and *MAP3K1*.

**Supplementary Table 9.** Full list of the 368 target genes analyzed in this study. HaloPlex probes were designed to capture whole exon regions of these genes. The coverage rate of each gene is shown.

**Supplementary Table 10.** Validation regions and primer sequences. Mutated regions validated by Sanger sequencing or quantitative PCR, and primer sequences used for validation.
